# Efficacy and tolerability of janus kinase inhibition strategies for moderate-to-severe Crohn’s disease: A systematic review and bayesian network meta-analysis

**DOI:** 10.1177/17562848261485778

**Published:** 2026-09-09

**Authors:** Hakim Ullah Wazir, Kartik Mehta, Muhammad Shikaib Shabbir, M. Rafiqul Islam, Muhammad Maaz, Maryam Muzaffar, Nimra Ehsan, Saba Aliha, Anchit Chauhan, Abdul Khurram, George Saffouri

**Affiliations:** 1 261614 Department of Internal Medicine, Lady Reading Hospital, Peshawar, Pakistan; 2 28862 Department of Internal Medicine, Maulana Azad Medical College, New Delhi, India; 3 Department of Gastroenterology and Hepatology, University of California, Riverside, USA; 4 Inland Empire Clinical Trials, University of California, Riverside, Rialto, USA; 5 Department of Gastroenterology and Hepatology, Jerry L. Pettis Memorial Veterans Hospital, Loma Linda; 6 Department of Internal Medicine, Shaheed Suhrawardy Medical College Hospital, Dhaka, Bangladesh; 7 473279 Department of Internal Medicine, Bacha Khan Medical College, Mardan, Pakistan; 8 66909 Department of Internal Medicine, Allama Iqbal Medical College, Lahore, Pakistan; 9 66942 Department of Internal Medicine, Khyber Medical College, Peshawar, Pakistan; 10 Department of Internal Medicine, Multan Medical and Dental College, Multan, Pakistan; 11 Department of Internal Medicine, 7712 University of Connecticut, Connecticut, USA

## Abstract

**Background:**

Crohn’s disease is a chronic inflammatory bowel disease with an expanding therapeutic landscape for moderate-to-severe disease. Janus kinase (JAK) inhibitors have emerged as effective oral treatment options by modulating intracellular inflammatory signalling pathways.

**Objectives:**

This systematic review and Bayesian Network meta-analysis aimed to synthesize randomized trial evidence evaluating JAK inhibition strategies in moderate-to-severe Crohn’s disease. Given the lack of head-to-head trials, indirect comparisons were considered exploratory.

**Design:**

Systematic review and Bayesian network meta-analysis of randomized controlled trials (RCTs).

**Data Sources and Methods:**

Cochrane Central Register of Controlled Trials, MEDLINE (PubMed), Embase, Scopus, and ClinicalTrials.gov were systematically searched from inception to April 2025. A Bayesian NMA was conducted in R. Hazard ratios (HRs) with 95% credible intervals (CrIs) were estimated. Surface under the cumulative ranking curve (SUCRA) values were calculated to rank interventions across efficacy and safety outcomes. The protocol is registered with PROSPERO.

**Results:**

Twelve RCTs comprising 25 treatment arms and 5129 patients were included. The network evaluated upadacitinib across six dosing regimens and filgotinib across two. Upadacitinib 45 mg once daily (OD) showed significant efficacy in achieving clinical remission compared to placebo (HR: 2.47; 95% CrI: 1.51–4.10). For endoscopic remission, upadacitinib 24 mg OD ranked highest, followed by upadacitinib 24 mg BID. For endoscopic response, upadacitinib 24 mg OD ranked highest. No statistically significant differences were observed for clinical response. These estimates were associated with very wide credible intervals indicating statistical imprecision. For safety outcomes, no statistically significant differences were observed among various regimens. For Inflammatory Bowel Disease Questionnaire (IBDQ) ≥ 16, all upadacitinib dosing regimens showed a clinically meaningful response.

**Conclusion:**

In this NMA, upadacitinib-containing regimens showed the most consistent efficacy signal among evaluated JAK inhibition strategies. However, the evidence network was sparse, limiting the clinical applicability. Treatment rankings should therefore be interpreted as exploratory.

## Introduction

Crohn’s disease is a chronic inflammatory disease of the gastrointestinal tract, marked by episodes of relapse and remission. It involves transmural inflammation that may lead to severe complications such as the formation of strictures, fistulas, or abscesses.^1^ The pathogenesis of Crohn’s disease is not fully understood, but may be linked to the patient’s genetic susceptibility, environmental factors, alterations in the gut microbiome, and a dysregulated immune response, which synergistically contribute to persistent inflammation and tissue damage.^2–4^ Several medical therapies, including corticosteroids, immunomodulators, and biologic agents targeting a variety of mechanisms have been used with variable degrees of success, but a significant proportion of patients still experience inadequate response, loss of response, or adverse effects to these treatments.^5–8^ This highlights the need for novel therapeutic strategies that offer improved safety and efficacy in Crohn’s disease management.^8,9^

Janus kinase (JAK) inhibitors, recently approved for Crohn’s disease, target intracellular signaling pathways of multiple cytokines, thereby reducing inflammation and promoting mucosal healing that contribute to the development of Crohn’s disease.^10–14^ A prior network meta-analysis assessed the comparative safety and efficacy of different JAK inhibitors in moderate to severe Crohn’s, but with the accumulation of recently published clinical trials, there is a compelling rationale to update the existing meta-analysis to provide a more comprehensive estimation of the safety and efficacy of JAK inhibitors for the management of Crohn’s disease.^15^

This updated meta-analysis aims to fill this gap by integrating the latest evidence and employing statistical methods to evaluate the relative effects of two approved JAK inhibitors – upadacitinib and filgotinib – in patients with moderate-to-severe Crohn’s disease.

## Methodology

### Material and methods

**Figure 1.**
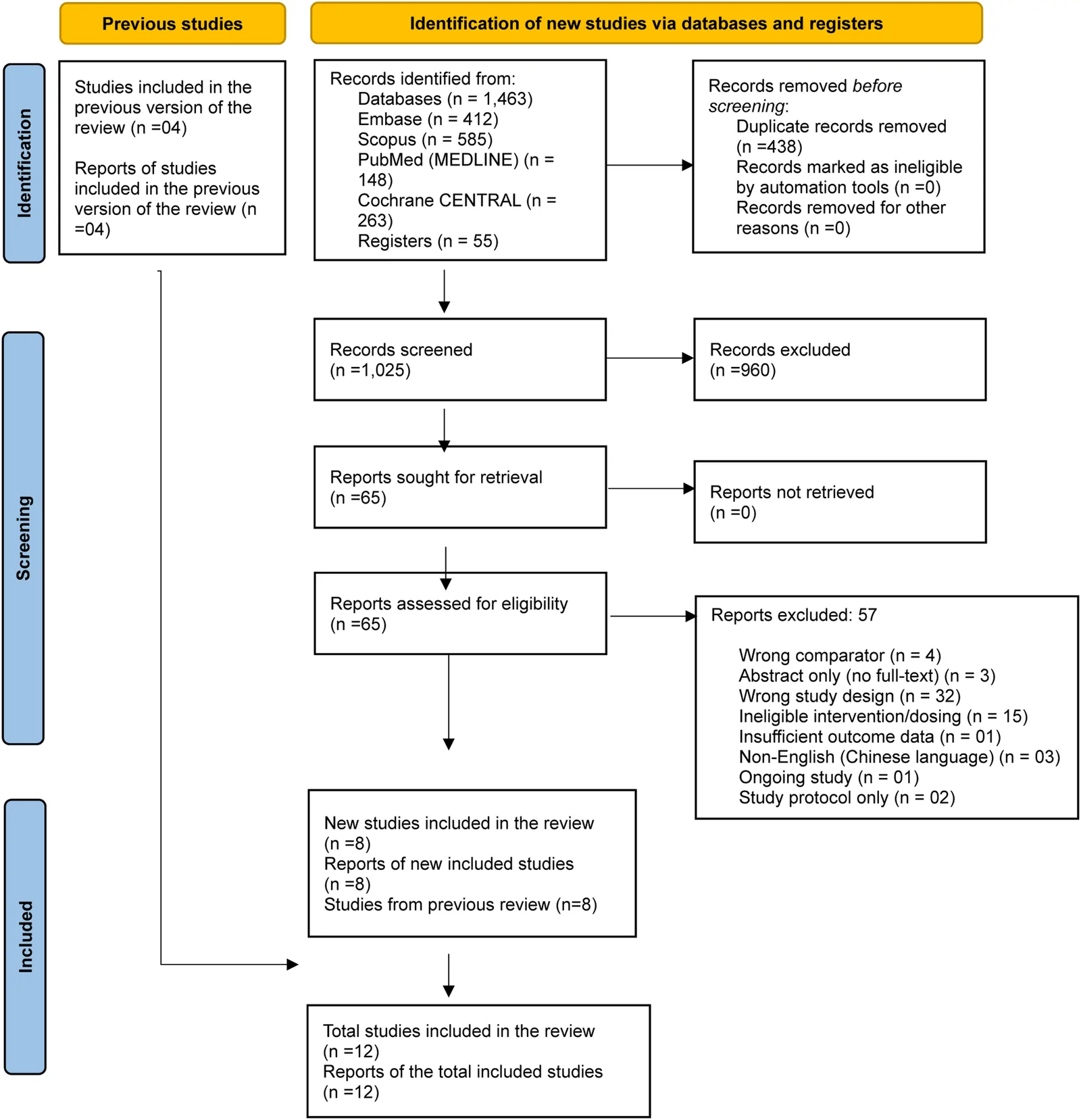
PRISMA Flow Diagram of the Study Selection Process.

Our meta-analysis was conducted according to the guidelines provided in the Cochrane handbook for systematic reviews of interventions and reported according to the preferred reporting items for systematic reviews and Meta-Analysis (PRISMA) statement.^16^ Our protocol is registered with PROSPERO (CRD420251019625). The PRISMA flowchart is available in Figure 1.

### Data sources and searches

Cochrane Central Register of Controlled Trials, MEDLINE, Embase, and ClinicalTrials.gov were used to perform a thorough electronic search from their inception to April 2025. Reference lists of included studies and similar systematic reviews were also screened to identify further pertinent studies.

The following terms (“Crohn’s Disease” OR “Crohns Disease” OR “Crohn’s Enteritis” OR “Inflammatory Bowel Disease” OR “Regional Enteritis” OR Ileocolitis OR “Terminal Ileitis” OR “Regional Ileitides” OR “Regional Ileitis” OR “Granulomatous Enteritis” OR “Granulomatous Colitis”) AND (“Janus Kinase Inhibitor” OR “JAK Inhibitor” OR “JAK Inhibitors” OR “upadacitinib” OR “ABT-494” OR “Rinvoq” OR “Filgotinib” OR “tasocitinib” OR “CP 690,550” OR “CP 690550” OR “CP690,550” OR “CP-690550” OR “CP690550” OR “tofacitinib citrate” OR “Xeljanz” OR “tofacitinib”) were used as either Medical Subject Heading (MeSH) terms or keywords.

### Eligibility criteria

This meta-analysis included randomized controlled trials (RCTs), assessing the comparative efficacy, and tolerability of Janus kinase inhibitors for moderate to severe Crohn’s disease. Parameters for exclusion included any study design other than RCTs, such as quasi-randomized trials, observational studies, and animal studies. No language or date restrictions were applied.

### Study selection an data extraction

We used Rayyan to narrow down and remove duplicates of all the articles yielded by our online search. Two authors independently screened titles and abstracts to exclude all irrelevant articles (S.A. and H.W.). Full-text screening following our eligibility criteria was then performed on the remaining studies. Any discrepancies over the selection of studies were settled by a third author (F.A.M.). Relevant data were extracted into a pre-piloted, password-protected Excel spreadsheet, including author name, publication year, country name, sample size, mean age, follow-up duration, interventional group, control group, conflict of interest, inclusion criteria, and exclusion criteria.

### Outcomes

The primary outcomes of our study were clinical remission and endoscopic response. Secondary outcomes were endoscopic remission, clinical response, total adverse events, serious adverse events, gastrointestinal perforation, drug discontinuation due to adverse events, and the Inflammatory Bowel Disease Questionnaire (IBDQ) at 12 weeks.

### Risk of bias assessment

The risk of bias in the included studies was evaluated using the revised Cochrane risk of bias tool for randomized trials (RoB 2.0).^17^ This tool assesses bias in five domains which comprise of [1] bias caused by the randomization process, [2] bias due to deviations from intended interventions, [3] bias arising from missing outcome data, [4] bias in the measurement of the outcome, and [5] bias in the selection of the reported result. The risk of bias for each included study was assessed by two investigators (A.C. and S.A.) with high, low, or some concerns of bias being reported. Any disagreement was settled by a senior investigator (H.W).

### Data synthesis and statistical analysis

A Bayesian network meta-analysis was performed on R software version 4.4.3. Pooled mean differences (MDs) and hazard ratios (HRs) with 95% credible intervals (CrIs) were approximated using a random or fixed effects model based on deviance information criteria (DIC). Surface Under the Cumulative Ranking Curve (SUCRA) values were calculated to rank interventions across outcomes, including clinical remission, endoscopic remission, clinical response, endoscopic response, IBDQ at WEEK 12, Safety profile, adverse events, and GI perforations. Analyses were performed in the intention-to-treat population; patients with missing outcome data or treatment discontinuation were treated as nonresponders where applicable. Funnel plot asymmetry was used to assess small-study and reporting bias. Network consistency was evaluated using node-splitting, and model convergence was assessed using trace plots and Gelman-Rubin potential scale reduction factors.

## Results

### Search results

A total of 1,463 articles were found in the databases search; 438 duplicates were removed, leaving 1,021 articles for screening; 956 articles were eliminated after title and abstract screening; 57 of the remaining 65 studies were removed after full-text screening, 4 due to wrong comparator, 03 due to abstract only, 32 due to incorrect study design, 15 due to ineligible interventions/dosing, 01 due to inadequate outcome data, 3 due to non-English language (Chinese), 01 due to ongoing study status, and 02 because they were study protocols only. Finally, 12 RCTs were included in this meta-analysis. The detailed screening process is depicted in Supplemental Table 1.

#### Study characteristics

Twelve RCTs that satisfied the eligibility requirements were included in our meta-analysis. The following baseline characteristics were extracted for baseline characteristics table (Supplemental Table 1): Age, gender, sample size, BMI, smoking status, country of origin, Crohn’s disease duration, SES score, location of Crohn’s disease, CDAI score at screening, Fecal calprotectin, CRP, previous biologics failure, previous TNF antagonist use, previous TBF antagonist failure, previous Ustekinumab failure, previous Vedolizumab use and failure, PDAI score, SF-36v2 PCS, SF-36v2 MCS, EQ-5D VAS, IBQD score, EQ-5D index score, FACTT-fatigue at baseline, WPAI-CD presentation, WPAI-CD absenteeism, WPAI-CD overall work impairment, WPAI-CD activity impairment. All included studies are multinational and multicenter. The studies were conducted across the globe, including the United States, Europe, Belgium, Canada, Australia, New Zealand, France, Italy, Germany, Poland, Hungary, Austria, the Netherlands, and Asian Pacific countries, etc.

#### Overview of included studies

A total of 12 RCTs comprising 25 treatment arms and 5,129 patients were included in the network meta-analysis. The network included two JAK inhibitors: upadacitinib (evaluated across six dosing regimens: 3 mg BID, 6 mg BID, 12 mg BID, 24 mg BID, 24 mg OD, and 45 mg OD) and filgotinib (100 mg OD and 200 mg OD), each compared against a common placebo comparator. As no direct head-to-head RCTs between upadacitinib and filgotinib exist, all cross-agent comparisons are indirect and should be interpreted as an exploratory synthesis of evidence rather than definitive comparative evidence.

### Clinical remission

**Figure 2.**
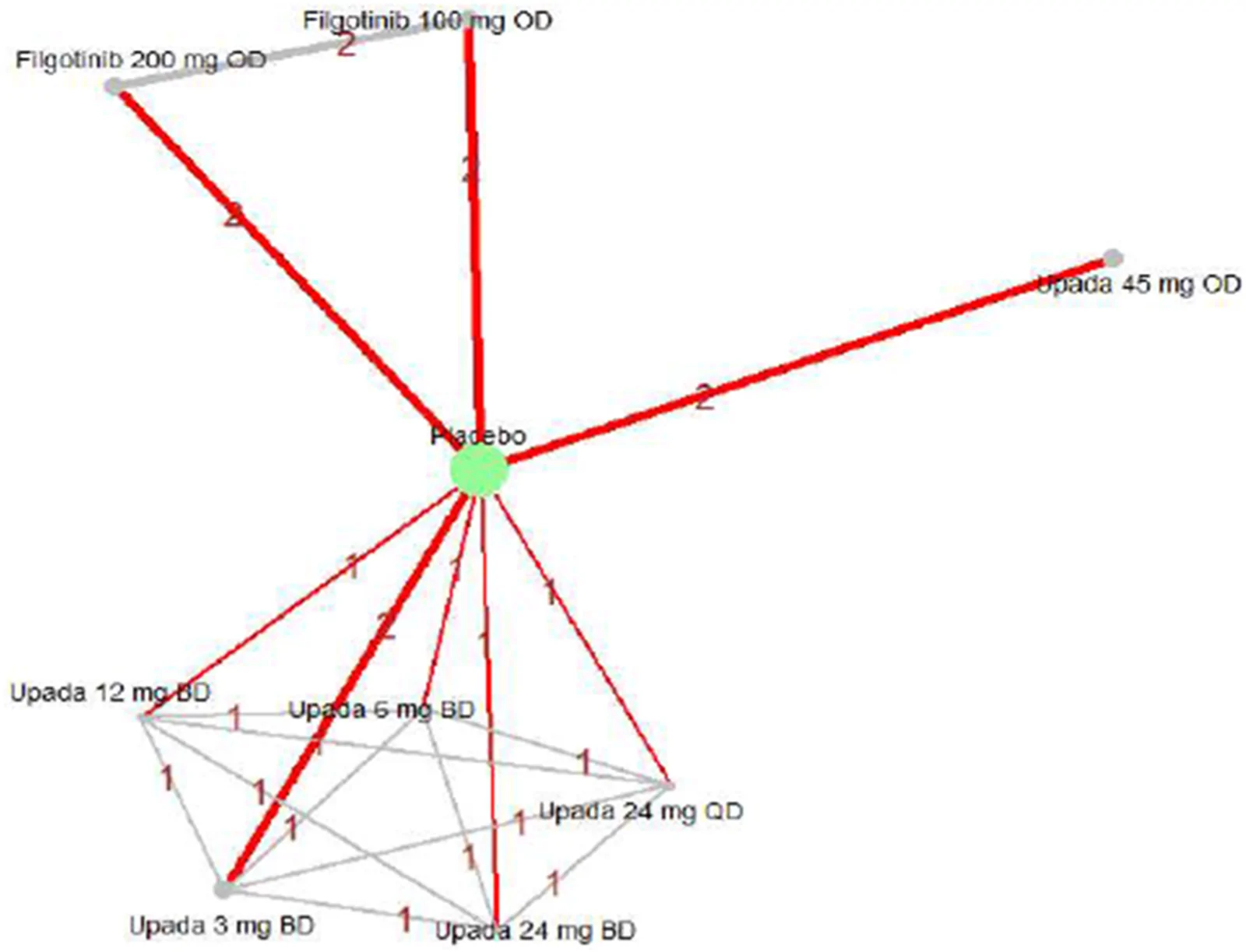
Network geometry plot for clinical remission.

**Figure 3.**
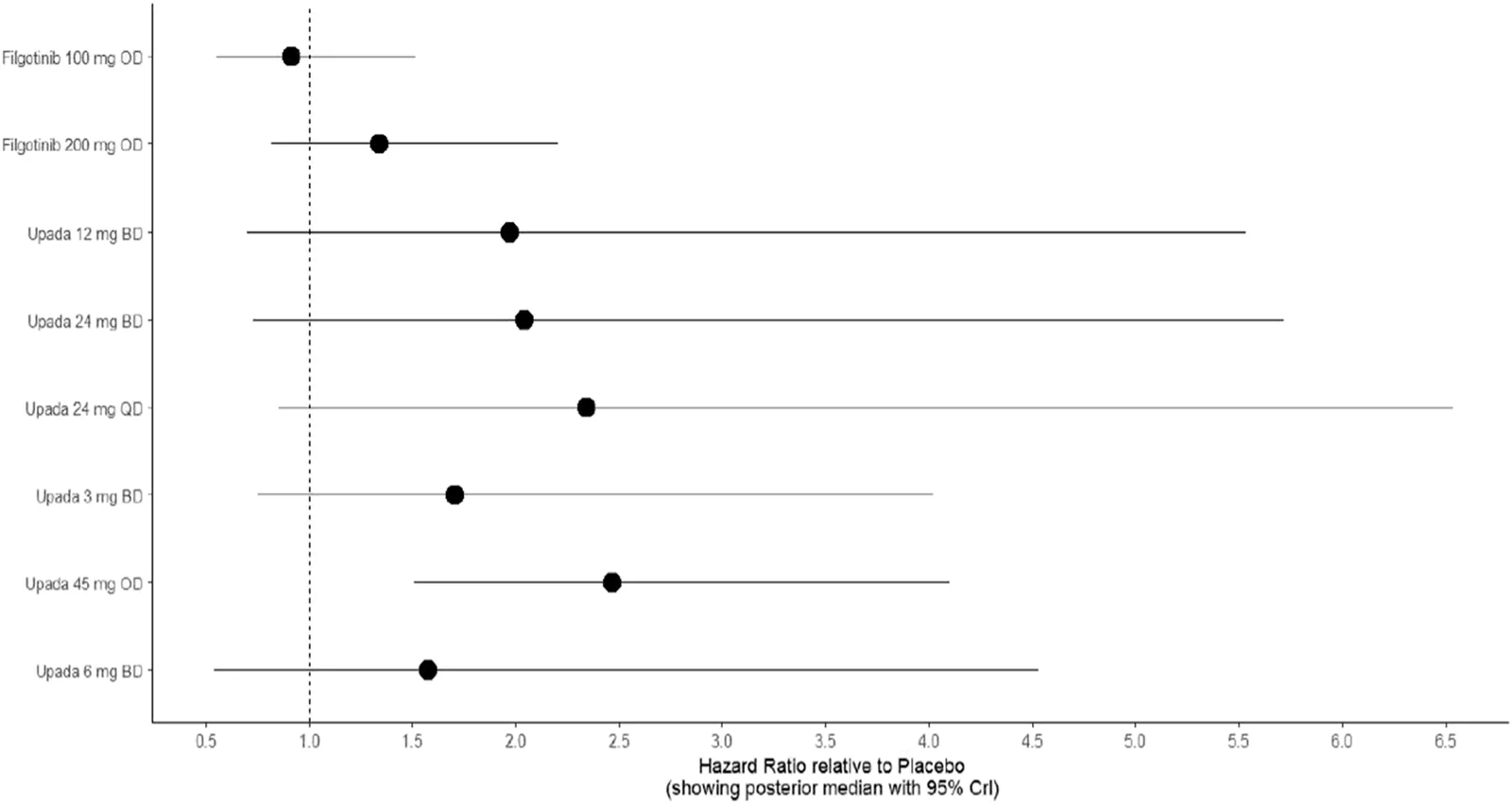
Forest plot for clinical remission.

**Figure 4.**
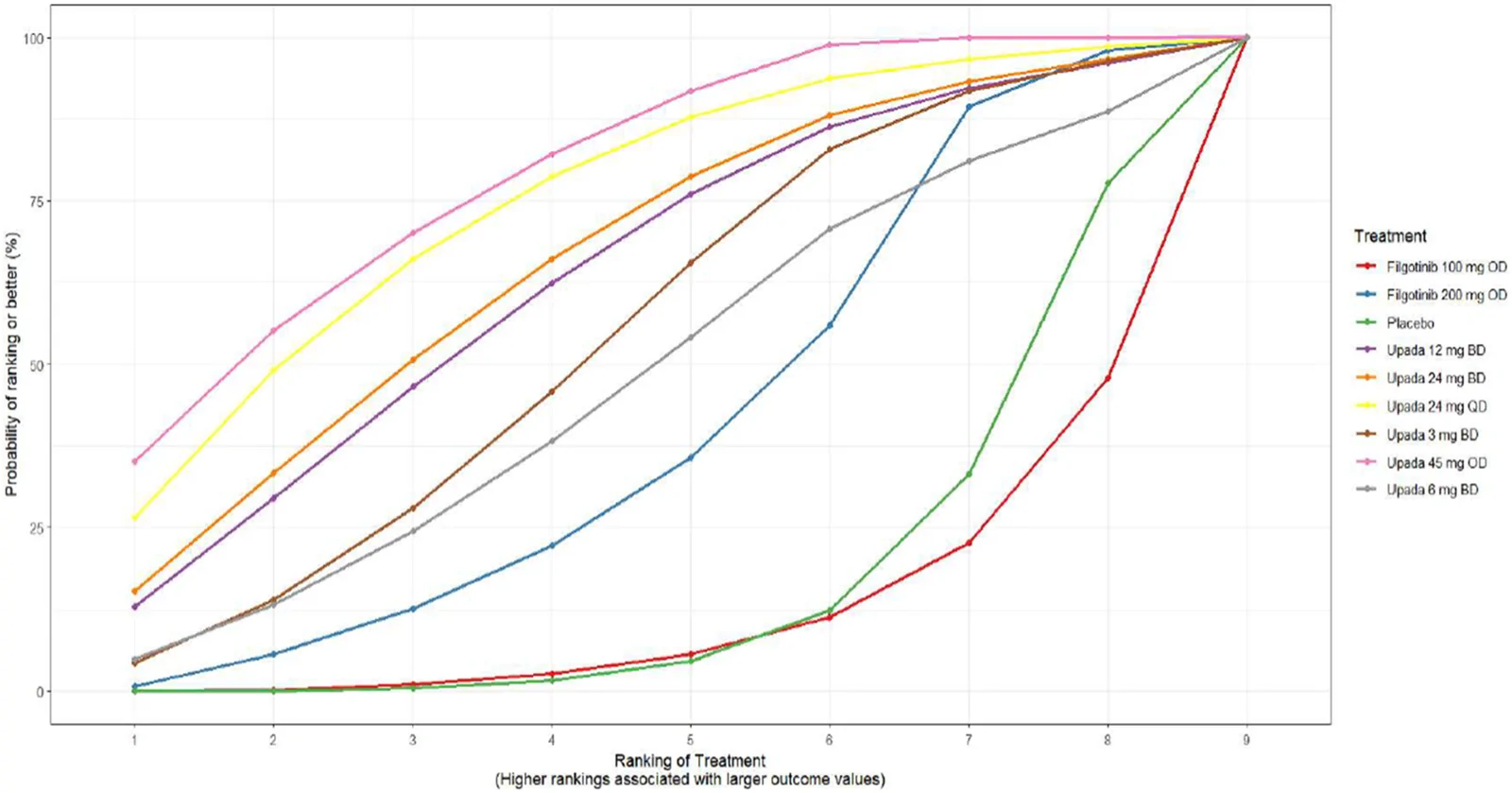
SUCRA plot for clinical remission.

A total of 19 randomized comparisons involving 2,481 patients from 6 RCTs evaluated upadacitinib and filgotinib regimens versus placebo for clinical remission. Among upadacitinib regimens, only upadacitinib 45 mg OD demonstrated statistically significant superiority over placebo (HR: 2.47; 95% CrI: 1.51–4.10; SUCRA: 79%). The 95% CrIs for all other upadacitinib dosing regimens (3 mg BID, 6 mg BID, 12 mg BID, 24 mg BID, 24 mg OD) crossed the null value, indicating no statistically significant effect at those doses. Among filgotinib regimens, neither filgotinib 200 mg OD nor filgotinib 100 mg OD achieved statistical significance for clinical remission (95% CrIs crossed null for both), consistent with Phase 3 DIVERSITY outcomes in which filgotinib did not meet the primary clinical remission endpoint. SUCRA rankings for all regimens are presented in Figures 2–4 and Supplemental Figure S1A–S1D.

### Endoscopic remission

**Figure 5.**
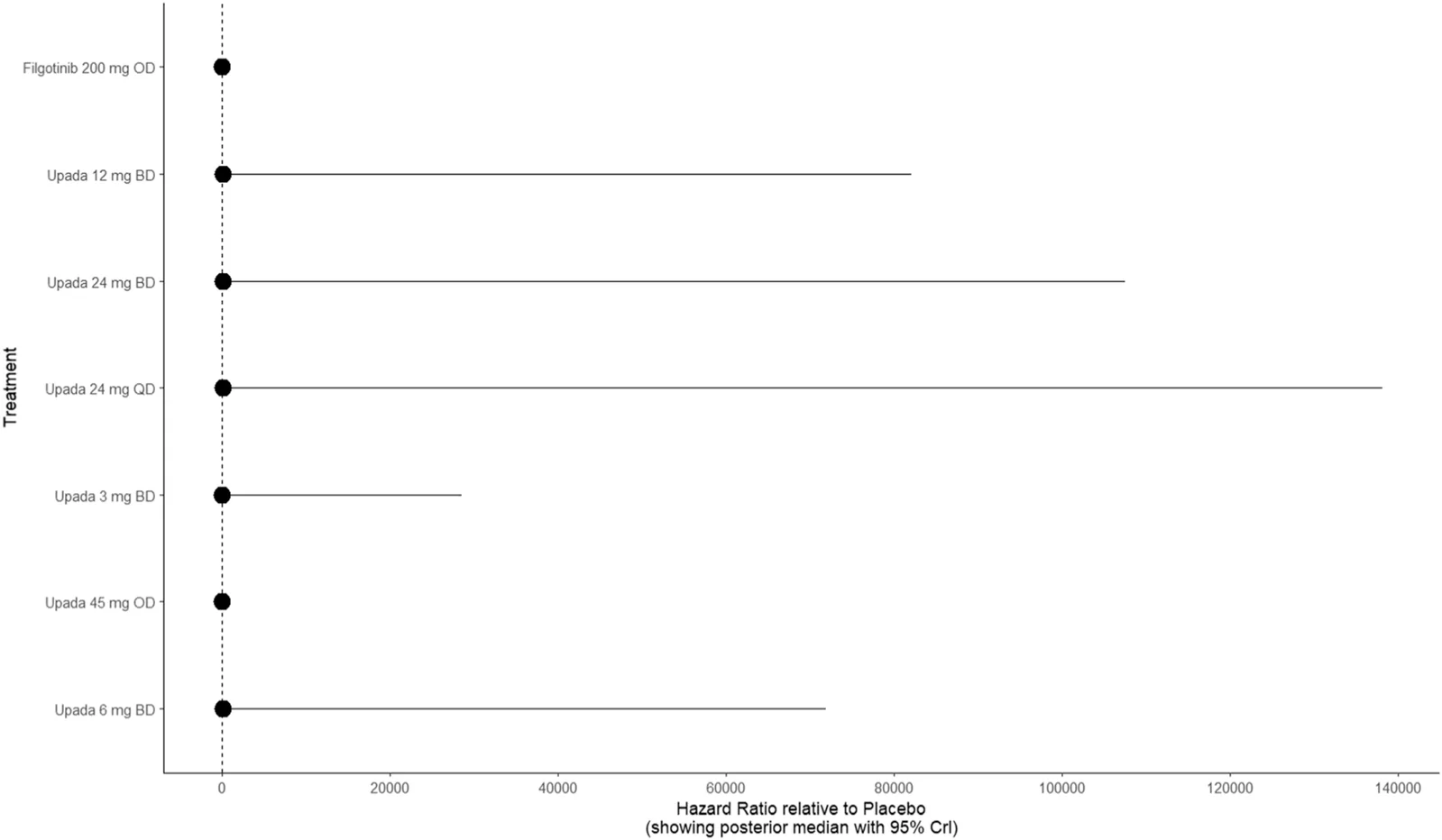
Forest plot for endoscopic remission.

A total of 17 randomized comparisons involving 1,336 patients from 4 RCTs evaluated upadacitinib and filgotinib regimens versus placebo for endoscopic remission. Among upadacitinib regimens, upadacitinib 24 mg OD ranked highest (HR: 103.67; 95% CrI: 3.85–138,064.75; SUCRA: 87%), followed by upadacitinib 24 mg BID (HR: 84.50; 95% CrI: 3.05–107,504.71; SUCRA: 80%), 12 mg BID (HR: 64.50; 95% CrI: 2.26–82,004.59; SUCRA: 71%), 6 mg BID (HR: 51.15; 95% CrI: 1.75–71,852.56; SUCRA: 64%), and 45 mg OD (HR: 5.89; 95% CrI: 2.79–13.86; SUCRA: 38%). Upadacitinib 3 mg BID did not reach statistical significance. Extremely wide credible intervals for several upadacitinib doses reflect the limited number of trials contributing endoscopic remission data and the resulting statistical imprecision. Therefore, the apparent ranking of upadacitinib 24 mg OD should be interpreted cautiously and should not be considered definitive evidence of comparative superiority over other upadacitinib doses or filgotinib. Among filgotinib regimens, filgotinib 200 mg OD did not demonstrate statistically significant endoscopic remission in this network (95% CrI crossed null). (Figure 5) (Supplemental Figure S2A–S2D).

### Clinical response

A total of 16 randomized comparisons involving 1,237 patients from 3 RCTs evaluated clinical response across upadacitinib dosing regimens. HRs ranged from 1.25 to 2.18, but none of the dosing regimens showed statistically significant clinical response (95% CrIs crossed null for all). SUCRA rankings were: upadacitinib 24 mg OD (74%) > 24 mg BID (70%) > 12 mg BID (66%) > 6 mg BID (58%) > 3 mg BID (44%) > 45 mg OD (30%). Filgotinib regimens were not included in the clinical response sub-network due to unavailability of extractable data for this endpoint from the included filgotinib trials (Supplemental Figure S3A–S3G).

### Endoscopic response

A total of 19 randomized comparisons encompassing 2,699 patients from 6 RCTs evaluated endoscopic response across filgotinib, upadacitinib, and placebo. Among upadacitinib regimens, upadacitinib 24 mg had the highest ranking probability (HR: 17.98; 95% CrI: 2.66–340.59; SUCRA: 85%), followed by 24 mg BID (HR: 17.29; 95% CrI: 2.56–324.17; SUCRA: 84%), 12 mg BID (HR: 13.33; 95% CrI: 1.93–253.36; SUCRA: 74%), 6 mg BID (HR: 8.72; 95% CrI: 1.18–167.59; SUCRA: 58%), and 45 mg OD (HR: 6.56; 95% CrI: 3.14–15.07; SUCRA: 57%). Upadacitinib 3 mg BID did not reach statistical significance. Wide credible intervals for most upadacitinib dose comparisons reflect substantial uncertainty from indirect comparisons. Among filgotinib regimens, neither filgotinib 200 mg OD nor filgotinib 100 mg OD achieved statistical significance for endoscopic response (95% CrIs crossed null for both). (Supplemental Figure S4A–S4G).

### Total adverse events

**Figure 6.**
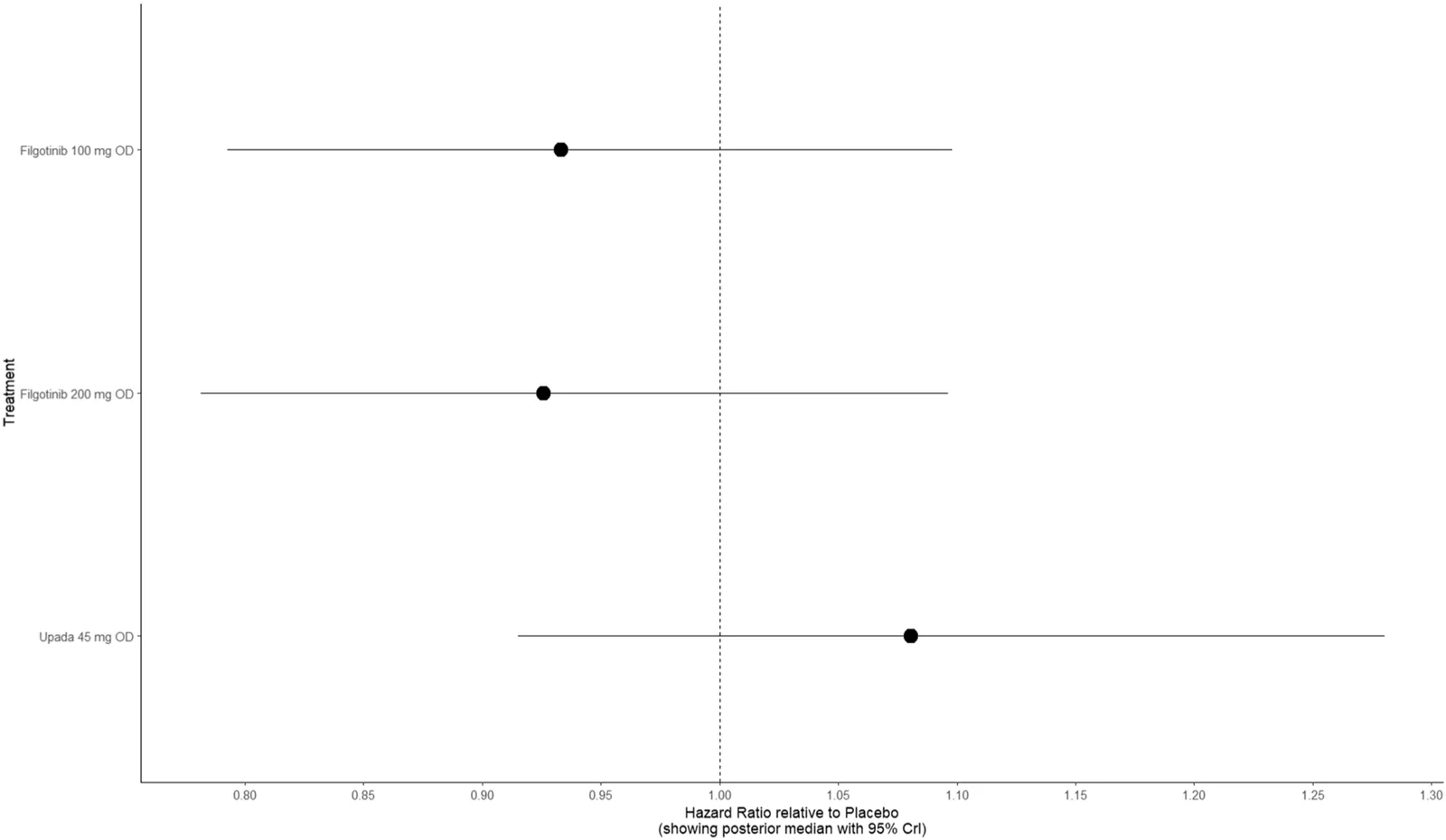
Forest plot for total adverse events.

A total of 4 randomized comparisons involving 2,441 patients across 5 RCTs compared upadacitinib 45 mg OD, filgotinib 200 mg OD, and filgotinib 100 mg OD against placebo for total adverse events. No statistically significant differences were observed for any regimen (all 95% CrIs crossed null). Filgotinib 200 mg OD and 100 mg OD demonstrated the most numerically favorable profiles (HR: 0.93 for both; SUCRA: 75% and 71%, respectively). Upadacitinib 45 mg OD showed a slightly higher point estimate (HR: 1.08; 95% CrI: 0.92–1.28; SUCRA: 36%), though not statistically significant. Overall, no clear differences in total adverse events were identified among the evaluated JAK inhibitor regimens and placebo. SUCRA-based safety rankings should be interpreted cautiously because they were based on limited comparisons and nonsignificant effect estimates (Figure 6) (Supplemental Figure S5A–S5D).

### Serious adverse events

**Figure 7.**
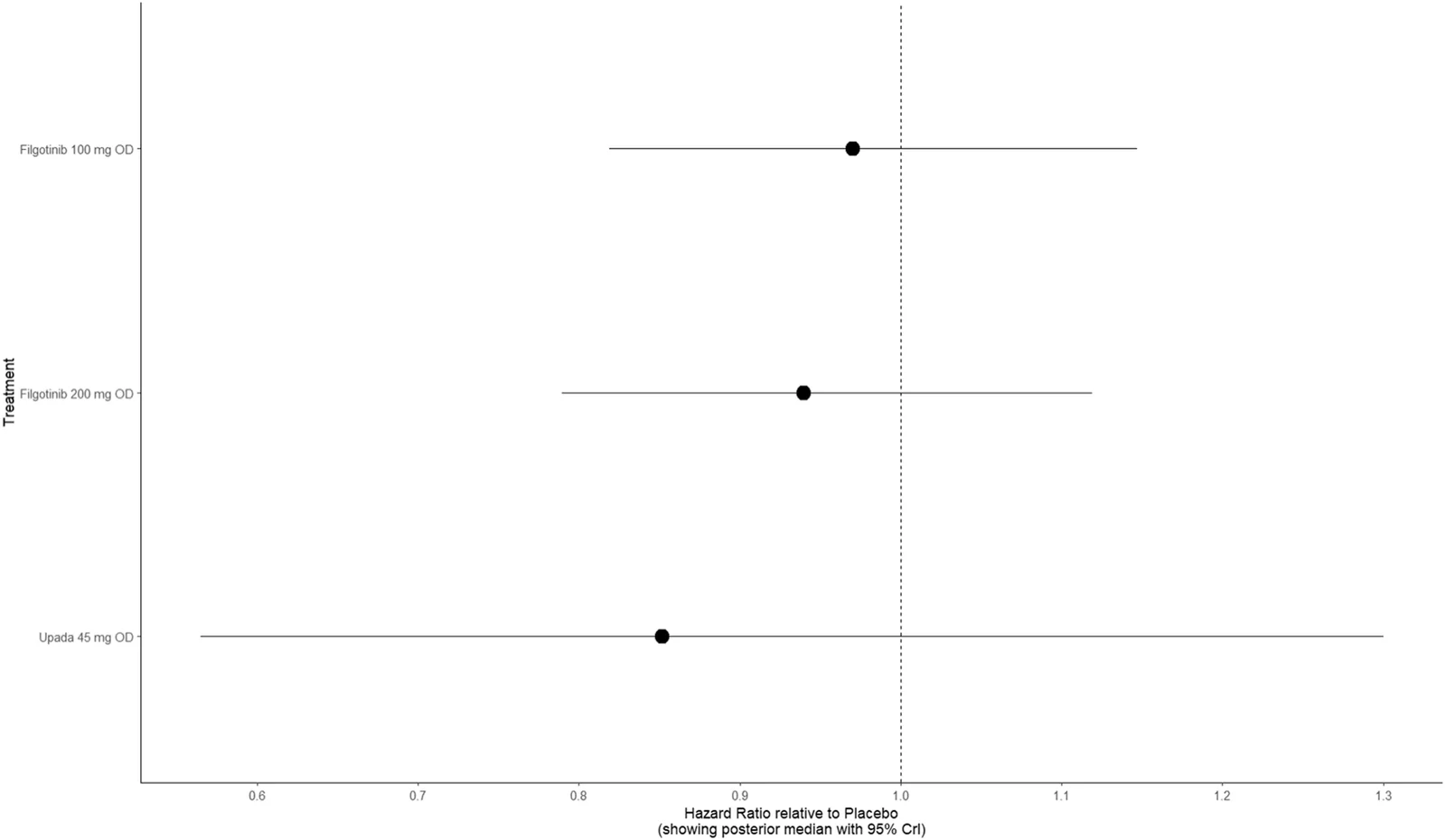
Forest plot for serious adverse events, https://creativecommons.org/licenses/by/4.0/.

A total of 4 randomized comparisons involving 2,441 patients from 5 RCTs assessed SAEs across upadacitinib 45 mg OD, filgotinib 200 mg OD, filgotinib 100 mg OD, and placebo. No statistically significant differences were observed for any regimen (all 95% CrIs crossed null). SUCRA rankings were: upadacitinib 45 mg OD (72%) > filgotinib 200 mg OD (58%) > filgotinib 100 mg OD (43%). These probabilistic rankings do not indicate statistical superiority and should be interpreted as exploratory (Figure 7) (Supplemental Figure S6A, S6B).

### Gastrointestinal perforation

A total of 4 randomized comparisons involving 1,915 patients from 4 RCTs assessed GI perforation across filgotinib 200 mg OD, filgotinib 100 mg OD, upadacitinib 45 mg OD, and placebo. HRs ranged from 0.95 to 1.84; none of the regimens showed statistical significance (all 95% CrIs crossed null). SUCRA rankings were: filgotinib 200 mg OD (64%) > upadacitinib 45 mg OD (45%) > filgotinib 100 mg OD (28%). Overall, no statistically significant increase in gastrointestinal perforation was observed for either upadacitinib or filgotinib in the available randomized trial evidence (Supplemental Figure S7A–S7F).

### Drug discontinuation due to adverse events

A total of 3 studies including 14,202 patients from 3 RCTs evaluated drug discontinuation for filgotinib 100 mg OD, filgotinib 200 mg OD, and placebo. Both filgotinib doses showed numerically higher but not statistically significant discontinuation rates versus placebo (95% CrIs crossed null). SUCRA rankings were: placebo (80%) > filgotinib 100 mg OD (63%) > filgotinib 200 mg OD (7%). Upadacitinib regimens were not included in this sub-network due to unavailability of extractable drug discontinuation data from the relevant upadacitinib trials (Supplemental Figure S8A–S8E).

### IBDQ at week 12

A total of 16 comparisons encompassing 1,241 patients from 3 RCTs reported IBDQ ≥16 improvement at week 12 across upadacitinib dosing regimens and placebo. All upadacitinib dosing regimens showed statistically significant improvements versus placebo, indicating a clinically meaningful quality-of-life response: upadacitinib 6 mg BID (HR: 2.97; 95% CrI: 1.41–6.73; SUCRA: 79%), 24 mg BID (HR: 2.87; 95% CrI: 1.35–6.54; SUCRA: 76%), 12 mg BID (HR: 2.45; 95% CrI: 1.13–5.62; SUCRA: 59%), 24 mg OD (HR: 2.35; 95% CrI: 1.07–5.41; SUCRA: 55%), 3 mg BID (HR: 2.19; 95% CrI: 1.01–5.01; SUCRA: 47%), and 45 mg OD (HR: 1.79; 95% CrI: 1.49–2.16; SUCRA: 34%). Although all upadacitinib dosing regimens favored treatment over placebo, the relative SUCRA rankings should be interpreted cautiously and should not be considered definitive evidence of dose-level superiority. Filgotinib regimens were not included in the IBDQ sub-network due to the unavailability of IBDQ ≥16 endpoint data from the included filgotinib trials. (Supplemental Figure S9A–S9E).

## Discussion

Crohn’s disease is associated with various disruptive complications that warrant urgent and timely management through medications such as antibiotics, immunomodulators, anti-inflammatory agents, biological therapy, as well as surgical intervention.^18^ For many patients with moderate to severe IBD, accessibility limitations, suboptimal response, treatment failure, and disease recurrence remain significant problems. Janus kinase inhibitors serve as a promising alternative treatment option in these cases owing to their oral formulation, potential cost effectiveness, rapid onset of action, and their developing therapeutic potential in mitigating symptoms in addition to endoscopic findings in moderate to severe IBD over the past few years.^18,19^ The JAK STAT signalling cascade plays a crucial role in the pathogenesis of Crohn’s disease by initiating the cytokine-dependent immune destruction.^20^

Our network meta-analysis was designed to integrate existing randomized data and compare the relative performance of different JAK inhibitors and dosing strategies in the treatment of moderate to severe Crohn’s disease. Our findings build on the recently published evidence syntheses, including the updated review of oral therapies by Noor et al. which emphasized the potential of JAK inhibitors as an important addition to the treatment options for patients who remain refractory to biologics, or for patients seeking oral alternatives, and the 2025 systematic review by Xu et al., which highlighted the strong overall performance of JAK1-selective agents.^21,22^

This network meta-analysis demonstrates that upadacitinib, particularly at higher induction doses, provided the most substantial improvements in clinical remission, endoscopic remission, endoscopic response, and quality-of-life outcomes among JAK inhibitors for moderate-to-severe Crohn’s disease. These results are consistent with the pivotal U-EXCEL and U-EXCEED trials, in which upadacitinib 45 mg OD achieved significant superiority over placebo across clinical and endoscopic endpoints.^23^ The high-level evidence from U-ACHIEVE and U-ACCOMPLISH trials further confirms the strong dose-dependent efficacy of upadacitinib in inducing mucosal healing in both biologic-experienced and biologic-naive populations.^24^

The numerically weaker efficacy estimates of filgotinib in our analysis is consistent with the contrast between its phase 2 and phase 3 data. The phase 2 FITZROY trial established filgotinib 200 mg once daily as an effective therapy for inducing clinical and endoscopic improvement in Crohn’s disease, demonstrating robust early-phase effect sizes. In contrast, the phase 3 DIVERSITY program evaluated a larger, more heterogeneous, and treatment-refractory population under stringent regulatory endpoints. Although the 200 mg dose continued to show improvements in both clinical and endoscopic outcomes, the effect estimates were attenuated and did not consistently meet primary regulatory thresholds. Safety findings were comparable between trials, but the overall benefit-risk balance in DIVERSITY was insufficient to support regulatory approval.^25,26^ These differences illustrate the typical attenuation of efficacy between early-phase and confirmatory trials in immune-mediated diseases.

Across all the safety endpoints, total adverse events, serious adverse events, gastrointestinal perforation, and drug discontinuation, our network found no statistically significant differences among the included regimens. These findings were consistent with the favorable safety profiles of upadacitinib and filgotinib in the comprehensive safety synthesis by Núñez et al., and reinforced by real-world data summarized by Weiss et al., which reported low absolute rates of venous thromboembolism (VTE), major cardiovascular events (MACE), serious infections, and malignancy for both agents.^27,28^ In addition, there have been no cases of lymphoma reported in randomized clinical trials involving the use of JAK inhibitors for Crohn’s disease. Even though there have been rare cases of malignancies, which include mainly non-melanoma skin cancers (NMSC), their scarcity, combined with the short study period, prevents conclusive evidence of the risk for lymphoma.

### Clinical implications

The prevalence of Crohn’s disease continues to rise in Western countries.^29^ The increased disease burden underscores the need for effective therapies. Biologic agents like TNF alpha inhibitors, anti-integrins, and interleukin inhibitors have improved patient outcomes, but also have potential limitations, including high cost, parenteral administration, immunogenicity, and safety concerns, including infection and malignancy risk.^30^ Janus Kinase inhibitors, with their oral route of administration and intracellular mechanism targeting key signaling pathways, represent a promising class of agents. Our findings position upadacitinib as the most effective oral agent, currently available for moderate to severe Crohn’s disease, with a favorable safety profile. This provides further evidence to integrate JAK inhibitors into the therapeutic management of Crohn’s disease.

### Strengths

Our study’s strengths include an updated review of the literature to more definitively assess the efficacy and safety of JAK inhibitors in moderate-severe Crohn’s disease. Network meta-analysis allows for indirect comparisons between JAK inhibitors, given the lack of direct, head-to-head, randomized controlled trials. This may help inform dosing and positioning of JAK inhibitors for both patients and clinicians.

### Limitations

Lack of head-to-head comparison in any network meta-analysis introduces bias, which should raise caution in interpretation and applicability. In general, all comparative efficacy and safety analyses were based on indirect comparisons. These results are not generalizable to other patient populations and require confirmation in larger patient populations and real-world settings. Long-term, head-to-head studies are needed to determine the relative efficacy and safety of JAK inhibitors in a larger number of patients with Crohn’s disease. A few of the included RCTs had relatively short follow-up durations (12 or 24 weeks), which are insufficient to fully capture the long-term safety profile of JAK inhibitors or to comprehensively assess the sustained clinical efficacy of these agents. In contrast, other trials included in the same network reported outcomes at the standard 52-week follow-up, which is more appropriate for evaluating both efficacy and safety. However, the inclusion of studies with varying follow-up durations within the same comparison groups introduce heterogeneity and may compromise the consistency and interpretability of pooled estimates.

The evidence base for several outcomes was sparse, with serious adverse events and gastrointestinal perforation each reported in only four RCTs, and other endpoints supported by similarly limited data. This limited the feasibility of meaningful subgroup analyses, heterogeneity assessment, and sensitivity analyses.

Another key limitation is the inability to perform subgroup analysis based on prior biologic exposure, due to lack of stratified outcome reporting in most of the included trials. In addition, a key factor to consider would be the possible impact of the phenotype of Crohn’s disease on treatment response. The site (L1-L3) and behavior (B1-B3) of Crohn’s disease based on the Montreal Classification can have an impact on the efficacy and safety profiles of JAK inhibitors. Unfortunately, most of the studies that were included in this review have not conducted analysis based on these sub-phenotypes, preventing further analysis for this network meta-analysis. Future randomized trials need to include and analyze data based on the sub-phenotypes to determine any differences in treatment response among the Crohn’s disease phenotypes.

Despite these limitations, our study contributes to the existing knowledge base on the risks associated with JAK inhibitors in the context of treating moderate-severe Crohn’s Disease.

## Conclusions

This network meta-analysis provides an updated synthesis of evidence evaluating JAK inhibition strategies for moderate-to-severe Crohn’s disease across clinical, endoscopic, quality-of-life, and safety outcomes. Overall, upadacitinib-containing regimens, particularly upadacitinib 45 mg once daily for clinical remission, showed the most consistent efficacy signal among the evaluated interventions. However, interpretation of cross-agent and dose-level comparisons is limited by the sparse evidence network, absence of direct head-to-head trials, reliance on placebo-controlled comparisons, and wide credible intervals for several outcomes, particularly endoscopic endpoints. Safety outcomes did not demonstrate statistically significant differences among the evaluated regimens and placebo, although the available evidence remains limited for uncommon adverse events. Collectively, these findings should be viewed as an exploratory evidence synthesis that may help contextualize the evolving role of JAK inhibition in Crohn’s disease and guide future head-to-head trials.

## Supplemental material

Supplemental material - Efficacy and tolerability of janus kinase inhibition strategies for moderate-to-severe Crohn’s disease: A systematic review and bayesian network meta-analysis Supplemental material for Efficacy and tolerability of janus kinase inhibition strategies for moderate-to-severe Crohn’s disease: A systematic review and bayesian network meta-analysis by Hakim Ullah Wazir, Kartik Mehta, Muhammad Shikaib Shabbir, M. Rafiqul Islam, Muhammad Maaz, Maryam Muzaffa, Nimra Ehsan, Saba Aliha, Anchit Chauhan, Abdul Khurram, George Saffouri in Therapeutic Advances in Gastroenterology.

Supplemental material - Efficacy and tolerability of janus kinase inhibition strategies for moderate-to-severe Crohn’s disease: A systematic review and bayesian network meta-analysis Supplemental material for Efficacy and tolerability of janus kinase inhibition strategies for moderate-to-severe Crohn’s disease: A systematic review and bayesian network meta-analysis by Hakim Ullah Wazir, Kartik Mehta, Muhammad Shikaib Shabbir, M. Rafiqul Islam, Muhammad Maaz, Maryam Muzaffa, Nimra Ehsan, Saba Aliha, Anchit Chauhan, Abdul Khurram, George Saffouri in Therapeutic Advances in Gastroenterology.

## Data Availability

All data generated or analyzed during this study are included in this article.*
