# Supplementary material for: Efficacy and tolerability of janus kinase inhibition strategies for moderate-to-severe Crohn’s disease: A systematic review and bayesian network meta-analysis
Source: Ther Adv Gastroenterol. 2026 Sep 9;19:17562848261485778. doi: 10.1177/17562848261485778 (PMC13558925; doi:10.1177/17562848261485778)
Supplement: Supplementary file 1 [file sj-pdf-1-tag-10.1177_17562848261485778.pdf]

Study Name			Loftus et al,2023				Ghosh et al 2024				Reinisch et al 2024			Reinisch et al 2023		Muhammad et al,2020					Peyrin-Biroulet et al,2021						Vermeire et al 2024 (A)			Vermeire et al 2024 (B)		
Trial Groups			U-EXCEL trial 1	U-EXCEED trial 2	U-ENDURE 15MG trial 3	U-ENDURE 30mg	U-EXCEL trial 1	U-EXCEED trial 2	U-ENDURE 15mg trial 3	U-ENDURE 30mg	Filgotinib 200 mg	Filgotinib 100 mg	Placebo group	Filgotinib	Placebo	3 mg b.i.d. Upadacitinib	6 mg b.i.d. Upadacitinib	12 mg b.i.d. Upadacitinib	24 mg b.i.d. Upadacitinib	24 mg q.d Upadacitinib	Upadacitinib 3mg BID,	Upadacitinib 6mg BID,	Upadacitinib 12mg BID,	Upadacitinib 24mg BID,	Upadacitinib 24mg QD		Filgotinib 100 mg	Filgotinib 200 mg	PLACEBO	Filgotinib 100 mg	Filgotinib 200 mg	placebo
			43 countries (USA, Europe, Asia)				USA,Canada,Brazil,Japan,Belgium,Portugal,Ireland				Austria, Belgium, Canada, France, Germany, Hungary, Italy, UK, and USA			Europe		19 Countries (USA,Europe,Israel,Australia,New Zealand etc)					40 countries (Europe countries,USA, Asian pacific countries etc)						USA, Netherlands, Austria, Germany, Italy, Switerland, Poland, and UK					
Sample Size (n)			526	495	502	1523	526	495	334	333	17	25	15	76	23	216	NA	NA	NA	NA	39	37	36	36	35		245	222	237	228	202	229
No. of Participants (n)	I		350	324	169	168	350	324	169	168	17	25	NA	76	NA	38	36	36	35	34	39	37	36	36	35		245	222	NA	228	202	NA
	C		176	171	165		176	171	165		15	15	NA	23	NA	37	37	37	37	37	37	37	37	37	37		237	237	NA	229	229	NA
Age in years (Mean, SD)	I		39.7 ± 13.7	38.4 ± 13.7	38.1 ± 13.5	37.0 ± 13.3	39.7 [13.7]	38.4 [13.7]	38.1 [13.5]	37.0 [13.3]	39 [11.2]	41 [14.0]	NA	35	NA	39.2 (13.9)	40.8 (13.5)	40.8 (15.2)	43.1 (9.64	40.4 (12.7)	37(19–66)	39(22–76)	41(19–70)	44(20–65)	41(21–64)		39 (14.1)	39 (13.8)	NA	42 (13.5)	39 (14.2)	NA
	C		39.3 ± 13.6	23.9 ± 6.2	24.6 ± 6.6		39.3 [13.6]	37.5 [12.1]	38.1 [13.0]		39 [11.8]	39 [11.8]	NA	37.33	NA	40.5 (12.1)	40.5 (12.1)	40.5 (12.1)	40.5 (12.1)	40.5 (12.1)	40(20–68)	40(20–68)	40(20–68)	40(20–68)	40(20–68)		38 (14.0)	38 (14.0)	NA	39 (12.5)	39 (12.5)	NA
Gender %	I	Male no. (%)	189	169	102	93	189 [54]	169 [52]	102 [60]	93 [55]	8 (47.1%)	15 (60.0%)	NA	31 (40.8)	NA	20 (53%)	16 (44%)	19 (53%)	10 (29%)	15 (44%)	20 (51.3%)	16 (43.2%)	19 (52.8%)	11 (30.6%)	16 (45.7%)		139 (57%)	112 (50%)	NA	101 (44%)	88 (44%)	NA
		Female	161	155	67	75	161(46)	155(47.8)	67(40)	75(45)	9 [52.9%]	10 [40.0%]	NA	45 (59.2)	NA	18 (47%)	20 (56%)	17 (47%)	25 (71%)	19 (56%)	19(48.7)	21(56.8)	17(47.2)	25(69.4)	19(54.3)		106 (43%)	110 (50%)	NA	127 (56%)	114 (56%)	NA
	C	Male	94	96	88		94 [53]	96 [56]	88 [53]	NA	11 (73.3%)	11 (73.3%)	NA	8 (34.8)	NA	13 (35%)	14 (35%)	15 (35%)	16 (35%)	17 (35%)	13 (35.1%)	13 (35.1%)	13 (35.1%)	13 (35.1%)	13 (35.1%)		107 (45%)	107 (45%)	NA	114 (50%)	114 (50%)	NA
		Female	82	75	77		82(46.5)	75(43.8)	77(46.6)	NA	4 [26.7%]	4 [26.7%]	NA	15 (65.2)	NA	24 (65%)	25 (65%)	26 (65%)	27 (65%)	28 (65%)	24(64.9)	24(64.9)	24(64.9)	24(64.9)	24(64.9)		130 (55%)	130 (55%)	NA	115 (50%)	115 (50%)	NA
BMI	I		24.5±6.0	24.2±6.0	24.1±6.0	24.2±6.6	24.5 [6.0]	24.2 [6.0]	24.1 [6.0]	24.2 [6.6]	NA	NA	NA	23.38 (3.78)	NA	NR	NR	NA	NA	NA	NA	NA	NA	NA	NA		24 (5.7)	23.9 (5.7)	NA	24.8 (5.7)	25.1 (6.4)	NA
	C		25.6±7.0	23.9±6.2	24.6±6.6		25.6 [7.0]	23.9 [6.2]	24.6 [6.6]	NA	NA	NA	NA	24.02 (4.26	NA	NR	NR	NA	NA	NA	NA	NA	NA	NA	NA		24.4 (6)	NA	NA	24.3 (6.1)	NA	NA
Crohn's Disease Duration-years (Mean ± SD)	I	< 1year	16.4 ± 8.9	18.5 ± 9.4	14 ± 7.4	14.9 ± 8.3	16.4 (8.9)	18.5(9.4)	14 (7.4)	14.9 (8.3)	NA	NA	NA	6.57 (4.99)	NA	NA	NA	NA	NA	NA	10.7(0.1–44.7)	8.8(2.2–46.4)	9.1(1.2–38.3)	14.1(1.1–35.5)	10.8(0.7–36.3)		25 (10%)	20 (9%)	NA	3 (1%)	0	NA
		> 1 year to < 3 years									NA	NA	NA		NA	NA	NA	NA	NA	NA	NA	NA	NA	NA	NA		40 (16%)	40 (18%)	NA	16 (7%)	13 (6%)	NA
		>3 years to < 7 years									NA	NA	NA		NA	NA	NA	NA	NA	NA	NA	NA	NA	NA	NA		58 (24%)	59 (27%)	NA	46 (20%)	53 (26%)	NA
		> or equal to 7 years									NA	NA	NA		NA	NA	NA	NA	NA	NA	NA	NA	NA	NA	NA		122 (50%)	103 (46%)	NA	163 (71%)	136 (67%)	NA
	C	< 1 year	14.5 ± 8.5	16.5 ± 8.4	16 ± 9	NA	14.5 (8.5)	16.5 (8.4)	16(9.06)	NA	NA	NA	NA	6.53 (5.45)	NA	NA	NA	NA	NA	NA	8.7(1.2–41.6)	8.7(1.2–41.6)	8.7(1.2–41.6)	8.7(1.2–41.6)	8.7(1.2–41.6)		24 (10%)	24 (10%)	NA	3 (1%)	3 (1%)	NA
		> 1 year to < 3 years				NA				NA	NA	NA	NA		NA	NA	NA	NA	NA	NA	NA	NA	NA	NA	NA		49 (21%)	49 (21%)	NA	23 (10%)	23 (10%)	NA
		>3 years to < 7 years				NA					NA	NA	NA		NA	NA	NA	NA	NA	NA	NA	NA	NA	NA	NA		40 (17%)	40 (17%)	NA	44 (19%)	44 (19%)	NA
		> or equal to 7 years				NA				NA	NA	NA	NA		NA	NA	NA	NA	NA	NA	NA	NA	NA	NA	NA		124 (52%)	124 (52%)	NA	159 (69%)	159 (69%)	NA
SES Score Mean	I		13.7 ± 7.3	15.2 ± 7.8	15.8 ± 7.6	15.5 ± 8.1	NA	NA	NA	NA	NA	NA	NA	13.50 (7.56)		14.6 (8.9)	16.3 (9.0)	15.6 (9.4)	14.0 (7.1)	13.2 (7.5)	NA	NA	NA	NA	NA		14 (7.9)	13 (7.1)	NA	15 (8·2)	15 (7·9)	NA
	C		13.6 ± 7.0	14.9 ± 7.8	14.8 ± 7.7	NA	NA	NA	NA	NA	NA	NA	NA	15.33 (8.69)		15.8 (8.6)	15.8 (8.6)	15.8 (8.6)	15.8 (8.6)	15.8 (8.6)	NA	NA	NA	NA	NA		13 (7.2)	13 (7.2)	NA	15 (7·8)	15 (7·8)	NA
Location of Crohn's Disease no. (%)	I	Ileum only	58	48	22	20	NA	NA	NA	NA	NA	NA	NA	NA	NA		NA	NA	NA	NA	NA	NA	NA	NA	NA		31 (13%)	31 (14%)	NA	39 (17%)	30 (15%)	NA
		Colon only	121	121	62	70	NA	NA	NA	NA	NA	NA	NA	NA	NA	NA	NA	NA	NA	NA	NA	NA	NA	NA	NA		104 (42%)	90 (41%)	NA	85 (37%)	67 (33%)	NA
		Ileum and Colon	171	164	85	78	NA	NA	NA	NA	NA	NA	NA	NA	NA	NA	NA	NA	NA	NA	NA	NA	NA	NA	NA		110 (45%)	101 (45%)	NA	104 (46%)	105 (52%)	NA
	C	Ileum only	27	23	24	NA	NA	NA	NA	NA	NA	NA	NA	NA	NA	NA	NA	NA	NA	NA	NA	NA	NA	NA	NA		33 (14%)	33 (14%)	NA	34 (15%)	34 (15%)	NA
		Colon only	57	68	67	NA	NA	NA	NA	NA	NA	NA	NA	NA	NA	NA	NA	NA	NA	NA	NA	NA	NA	NA	NA		98 (41%)	98 (41%)	NA	106 (46%)	106 (46%)	NA

		Ileum and Colon	92	80	74	NA	NA	NA	NA	NA	NA	NA	NA	NA	NA	NA	NA	NA	NA	NA	NA	NA	NA	NA	NA		106 (45%)	106 (45%)	NA	89 (39%)	89 (39%)	NA
**CDAI score at screening**	I		292.4 ± 81.3	306.6 ± 89.4	300.8 ± 90.8	312.1 ± 75.4	NA	NA	NA	NA	190 [62.4]	194 [67.1]	NA	295.00 (47.99)	NA	296 (59.7)	308 (55.1)	305 (60.0)	291 (68.8)	313 (54.4)	296(230–599)	NA	280(224–446)	278(162–556)	305(231–421)		322 (55·5)	322 (55·5)	NA	321 (55·7)	306 (54·0)	NA
	C		293.9 ± 85.4	308.1 ± 84.3	308.4 ± 82.3	NA	NA	NA	NA	NA	190 [57.9]	190 [57.9]	NA	275.33 (47.41)	NA	288 (60.0)	289 (60.0)	290 (60.0)	291 (60.0)	292 (60.0)	276(188–447)	276(188–447)	276(188–447)	276(188–447)	276(188–447)		320 (59·4)	320 (59·4)	NA	322 (57·5)	322 (57·5)	NA
**Fecal Calprotectin mg (mean ± SD)**	I		7659.5 ± 4986.3	7728 ± 5023.7	8036.5 ± 5446.6	7818 ± 5460.7	NA	NA	NA	NA	1103 (± 335)	1273 (± 2287)	NA	318.00 (291.32)	NA	NA	NA	NA	NA	NA	NA	NA	NA	NA	NA		1870 (2944.5)	1891 (2725.7)	NA	1833 (3117·9)	1864 (3208·1)	NA
	C		6540.5 ± 4545.0	5341 ± 3587	4817 ± 3205.6	NA	NA	NA	NA	NA	1186 (± 2278)	1186 (± 2278)	NA	247 (173.3)	NA	NA	NA	NA	NA	NA	NA	NA	NA	NA	NA		1912 (3719.6)	1912 (3719.6)	NA	1882 (3016·2)	1882 (3016·2)	
**CRP mg/L**	I		34.1 ± 20.6	41.3 ± 24.9	32.9 ± 20.5,	35.7 ± 23.1	NA	NA	NA	NA	8.6 (± 10.3)	14.7 (± 21.3)	NA	7.2 (9.8)	NA	NA	NA	NA	NA	NA	NA	NA	NA	NA	NA		18.7 (27.3)	15.4 (23.4)	NA	20·0 (29·1)	23·9 (33·7)	
	C		31.8 ± 20.9	36.3 ± 23.5	28.6 ± 18.1	NA	NA	NA	NA	NA	13.1 (± 14.4)	13.1 (± 14.4)	NA	5.3 (6.7)	NA	NA	NA	NA	NA	NA	NA	NA	NA	NA	NA		16.4 (25.3)	16.4 (25.3)	NA	18·6 (23·4)	18·6 (23·4)	
**Previous Biologics Therapy Failure n/N**	I	0	NA	NA	NA	NA	NA	NA	NA	NA	NA	NA	NA	46 (60.5)	NA	NA	NA	NA	NA	NA	NA	NA	NA	NA	NA		NA	NA	NA	NA	NA	NA
		1	58/161 (36.0)	126/324 (38.9)	52/124 (41.9)	43/127 (33.9)	58 (36)	126 [39]	52 [42]	43 [34	NA	NA	NA	29 (38.2)	NA	NA	NA	NA	NA	NA	NA	NA	NA	NA	NA		NA	NA		NA	NA	NA
		2	52/161 (32.3)	92/324 (28.4)	31/124 (25.0	35/127 (27.6)	52 [32]	92 [28]	31 [25]	35 [28	NA	NA	NA	22 (52.4)	NA	NA	NA	NA	NA	NA	NA	NA	NA	NA	NA		NA	NA	NA	NA	NA	NA
		3 or more	51/161 (31.7)	106/324 (32.7)	41/124 (33.1)	49/127 (38.6)	51 [32]	106 [33]	41 [33]	49 [39]	6 [35.3%]	12 [48.0%]	NA	NA	NA	NA	NA	NA	NA	NA	NA	NA	NA	NA	NA		NA	NA	NA	NA	NA	NA
	C	0	NA	NA	NA	NA	NA	NA	NA	NA	NA	NA	NA	17 (73.9)	NA	NA	NA	NA	NA	NA	NA	NA	NA	NA	NA		NA	NA	NA	NA	NA	NA
		1	28/78 (35.9)	68/171 (39.8)	52/126 (41.3)	NA	28 [36]	68 [40]	52 [41]	NA	NA	NA	NA	15 (71.4)	NA	NA	NA	NA	NA	NA	NA	NA	NA	NA	NA		NA	NA	NA	NA	NA	NA
		2	24/78 (30.8)	55/171 (32.2)	32/126 (25.4)	NA	24 [31]	55 [32]	32 [25]	NA	NA	NA	NA	13 (72.2)	NA	NA	NA	NA	NA	NA	NA	NA	NA	NA	NA		NA	NA	NA	NA	NA	NA
		3 or more	26/78 (33.3)	48/171 (28.1)	42/126 (33.3)	NA	26 [33]	48 [28]	42 [33]	NA	4 [26.7%]	4 [26.7%]	NA	NA	NA	NA	NA	NA	NA	NA	NA	NA	NA	NA	NA		NA	NA	NA	NA	NA	NA
**Previous TNF antagonist use (%)**	I					NA	NA	NA	NA	NA	NA	NA	NA	46 (60.5)	NA	NA	NA	NA	NA	NA	37	36	34	36	33		109 (44%)	97 (44%)		224 (98%)	197 (98%)	
	C					NA	NA	NA	NA	NA	NA	NA	NA	17 (73.9)	NA	NA	NA	NA	NA	NA	35	35	35	35	35		108 (46%)	108 (46%)		219 (96%	219 (96%	
**Previous failure of TNF antagonist (%)**	I		157(97.5)	308(95.1)	117(94.4)	123(96.6)	NA	NA	NA	NA	12 [70.6%]	16 [64.0%],	NA	NA	NA	NA	NA	NA	NA	NA	NA	NA	NA	NA	NA		91 (37%)	80 (36%)		198 (87%)	175 (87%)	
	C		75(96.2)	164(95.9)	118(93.7)	118(93.7)	NA	NA	NA	NA	9 [60.0%]	9 [60.0%]	NA	NA	NA	NA	NA	NA	NA	NA	NA	NA	NA	NA	NA		93 (39%)	93 (39%)		186 (81%)	186 (81%)	
**Previous Ustekinumab failed n (%)**	I	NA	64(39.8)	118(36.4)	41(33.1)	49(38.6)	NA	NA	NA	NA	3 [17.6%]	10 [40.0%]	NA	NA	NA	NA	NA	NA	NA	NA	NA	NA	NA	NA	NA		NA	NA				
	C	NA	33(42.3)	57(33.3)	48(38.1)	48(38.1)	NA	NA	NA	NA	3 [20.0%]	3 [20.0%]	NA	NA	NA	NA	NA	NA	NA	NA	NA	NA	NA	NA	NA		NA	NA				
**Previous Vedolizumab use (%)**	I	NA	NA	NA	NA	NA	NA	NA	NA	NA	NA	NA	NA	NA	NA	NA	NA	NA	NA	NA	NA	NA	NA	NA	NA		71 (29%)	71 (29%)		100 (44%)	106 (52%)	
	C	NA	NA	NA	NA	NA	NA	NA	NA	NA	NA	NA	NA	NA	NA	NA	NA	NA	NA	NA	NA	NA	NA				66 (28%)	66 (28%)		97 (42%)	97 (42%)	
**Previous failure of Vedolizumab n (%)**	I	NA	49(30.4)	99(30.6)	39(31.5	43(33.9)	NA	NA	NA	NA	5 [29.4%]	2 [8.0%]	NA	NA	NA	NA	NA	NA	NA	NA	NA	NA	NA	NA	NA		65 (27%)	62 (28%)		87 (38%)	94 (47%)	
	C	NA	25(32.10	47(27.5)	38(30.2)	NA	NA	NA	NA	NA	2 [13.3%]	2 [13.3%]	NA	NA	NA	NA	NA	NA	NA	NA	NA	NA	NA	NA	NA		58 (24%)	58 (24%)		85 (37%)	85 (37%)	NA
**Smoking Status**	I	NA	NA	NA	NA	NA	NA	NA	NA	NA	5 [29.4%]	6 [24.0%]	NA	NA	NA	NA	NA	NA	NA	NA	NA	NA	NA	NA	NA		NA	NA	NA	NA	NA	NA
	C	NA	NA	NA	NA	NA	NA	NA	NA	NA	4 [26.7%],	4 [26.7%],	NA	NA	NA	NA	NA	NA	NA	NA	NA	NA	NA	NA	NA		NA	NA	NA	NA	NA	NA

**PDAI score**	I	NA	NA	NA	NA	NA	NA	NA	NA	NA	9 [3.1]	8 [3.4]	NA	NA	NA	NA	NA	NA	NA	NA	NA	NA	NA	NA	NA		NA	NA	NA	NA	NA	NA
	C	NA	NA	NA	NA	NA	NA	NA	NA	NA	7 [3.1]	7 [3.1]	NA	NA	NA	NA	NA	NA	NA	NA	NA	NA	NA	NA	NA		NA	NA	NA	NA	NA	NA
**SF-36v2 PCS(physical component summary)**	I	NA	NA	NA	NA	NA	39.1 [8.1]	38.4 [8.1]	39.4 [8.6]	38.6 [8.0]	NA	NA	NA	NA	NA	NA	NA	NA	NA	NA	NA	NA	NA	NA	NA		NA	NA	NA	NA	NA	NA
	C	NA	NA	NA	NA	NA	39.6 [8.1]	38.4 [8.4]	37.6 [7.9]	NA	NA	NA	NA	NA	NA	NA	NA	NA	NA	NA	NA	NA	NA	NA	NA		NA	NA	NA	NA	NA	NA
**SF-36v2 MCS(mental component summary)**	I	NA	NA	NA	NA	NA	39.2 [10.5]	39.6 [11.0]	39.1 [11.0]	39.3 [9.9]	NA	NA	NA	NA	NA	NA	NA	NA	NA	NA	NA	NA	NA	NA	NA		NA	NA	NA	NA	NA	NA
	C	NA	NA	NA	NA	NA	37.8 [11.2]	38.8 [11.5]	38.6 [10.4]	NA	NA	NA	NA	NA		NA	NA	NA	NA	NA	NA	NA	NA	NA	NA		NA	NA	NA	NA	NA	NA
**EQ-5D VAS(visual analogue scale)**	I	NA	NA	NA	NA	NA	52.6 [20.7]	50.9 [21.3]	51.1 [21.2]	52.8 [18.5]	NA	NA	NA	NA	NA	NA	NA	NA	NA	NA	49.4 ± 18.7	47.8 ± 16.4	50.7 ± 17.6	51.6 ± 18.5	50.7 ± 17.0		NA	NA	NA	NA	NA	NA
	C	NA	NA	NA	NA	NA	50.5 [21.2]	49.3 [20.8]	50.1 [20.9]	NA	NA	NA	NA	NA	NA	NA	NA	NA	NA	NA	48.6 ± 14.6	48.6 ± 14.7	48.6 ± 14.8	48.6 ± 14.9	48.6 ± 14.10		NA	NA	NA	NA	NA	NA
**IBQD score**	I	NA	NA	NA	NA	NA	122.0 [34.3]	120.5 [33.8	121.8 [33.9]	120.1 [30.9]	NA	NA	NA	NA	NA	NA	NA	NA	NA	NA	115.2 ± 27.5	113.7 ± 25.9	115.2 ± 36.1	113.8 ± 36.0	120.7 ± 36.3		NA	NA	NA	NA	NA	NA
	C	NA	NA	NA	NA	NA	118.0 [33.8]	117.2 [31.4]	117.3 [34.8]	NA	NA	NA	NA	NA	NA	NA	NA	NA	NA	NA	118.0 ± 28.5	118.0 ± 28.6	118.0 ± 28.7	118.0 ± 28.8	118.0 ± 28.9		NA	NA		NA	NA	NA
**EQ-5D index score**	I	NA	NA	NA	NA	NA	52.6 [20.7]	50.9 [21.3]	51.1 [21.2]	52.8 [18.5]	NA	NA	NA	NA	NA	NA	NA	NA	NA	NA	0.69 ± 0.17	0.71 ± 0.18	0.67 ± 0.21	0.69 ± 0.20	0.68 ± 0.22		NA	NA	NA	NA	NA	NA
	C	NA	NA	NA	NA	NA	50.5 [21.2]	49.3 [20.8]	50.1 [20.9		NA	NA	NA	NA	NA	NA	NA	NA	NA	NA	0.65 ± 0.24	0.65 ± 0.25	0.65 ± 0.26	0.65 ± 0.27	0.65 ± 0.28		NA	NA	NA	NA	NA	NA
**FACIT-Fatigue at baseline, mean [SD]**	I	NA	NA	NA	NA	NA	24.5 [12.1]	23.5 [12.2]	24.9 [12.5]	23.2 [11.5	NA	NA	NA	NA	NA	NA	NA	NA	NA	NA	NA	NA	NA	NA	NA		NA	NA	NA	NA	NA	NA
	C	NA	NA	NA	NA	NA	23.5 [12.3]	23.8 [12.4]	22.9 [11.9]	NA	NA	NA	NA	NA	NA	NA	NA	NA	NA	NA	NA	NA	NA	NA	NA		NA	NA	NA	NA	NA	NA
**WPAI-CD presenteeism**	I	NA	NA	NA	NA	NA	47.1 [23.6]	48.3 [23.8	49.2 [25.4]	47.3 [22.3]	NA	NA	NA	NA	NA	NA	NA	NA	NA	NA	58.8 ± 26.4	57.2 ± 23.2	47.3 ± 27.9	47.5 ± 25.5	47.0 ± 26.5		NA	NA	NA	NA	NA	NA
	C	NA	NA	NA	NA		50.0 [25.2]	45.4 [22.9]	50.4 [24.6	NA	NA	NA	NA	NA	NA	NA	NA	NA	NA	NA	41.8 ± 23.2	41.8 ± 23.3	41.8 ± 23.4	41.8 ± 23.5	41.8 ± 23.6		NA	NA	NA	NA	NA	NA
**WPAI-CD absenteeism**	I	NA	NA	NA	NA	NA	19.5 [28.9]	22.8 [30.7]	21.1 [29.9]	24.1 [33.3	NA	NA	NA	NA	NA	NA	NA	NA	NA	NA	24.0 ± 36.0	15.6 ± 26.2	23.4 ± 40.2	15.2 ± 26.5	20.7 ± 31.4		NA	NA	NA	NA	NA	NA
	C	NA	NA	NA	NA	NA	20.7 [27.4]	18.8. [29.6]	21.1 [26.9]	NA	NA	NA	NA	NA	NA	NA	NA	NA	NA	NA	19.8 ± 29.5	19.8 ± 29.6	19.8 ± 29.7	19.8 ± 29.8	19.8 ± 29.9		NA	NA	NA	NA	NA	NA
**WPAI-CD overall work impairmen**	I	NA	NA	NA	NA	NA	55.6 [27.5	58.6 [28.0]	57.6 [28.7]	58.8 [27.2	NA	NA	NA	NA	NA	NA	NA	NA	NA	NA	66.5 ± 27.4	61.4 ± 25.0	57.1 ± 29.3	52.4 ± 29.8	53.3 ± 30.7		NA	NA	NA	NA	NA	NA
	C	NA	NA	NA	NA	NA	58.1 [28.2	54.5 [27.1]	59.1 [27.7]		NA	NA	NA	NA	NA	NA	NA	NA	NA	NA	52.1 ± 29.1	52.1 ± 29.2	52.1 ± 29.3	52.1 ± 29.4	52.1 ± 29.5		NA	NA	NA	NA	NA	NA
**WPAI-CD activity impairment**	I	NA	NA	NA	NA	NA	55.4 [26.2]	56.4 [25.0	55.6 [27.2]	56.0 [23.3]	NA	NA	NA	NA	NA	NA	NA	NA	NA	NA	63.3 ± 19.8	61.9 ± 18.2	62.6 ± 24.2	57.5 ± 23.5	60.6 ± 24.1		NA	NA	NA	NA	NA	NA
	C	NA	NA	NA	NA	NA	56.4 [25.8]	58.4 [24.4]	58.7 [24.5]	NA	NA	NA	NA	NA	NA	NA	NA	NA	NA		60.3 ± 26.5	60.3 ± 26.6	60.3 ± 26.7	60.3 ± 26.8	60.3 ± 26.9		NA	NA	NA	NA	NA	NA
