# Supplementary material for: Efficacy and tolerability of janus kinase inhibition strategies for moderate-to-severe Crohn’s disease: A systematic review and bayesian network meta-analysis
Source: Ther Adv Gastroenterol. 2026 Sep 9;19:17562848261485778. doi: 10.1177/17562848261485778 (PMC13558925; doi:10.1177/17562848261485778)
Supplement: Supplementary file 2 [file sj-pdf-2-tag-10.1177_17562848261485778.pdf]

SUPPLEMENTARY FIGURES:

CLINICAL REMISSION:

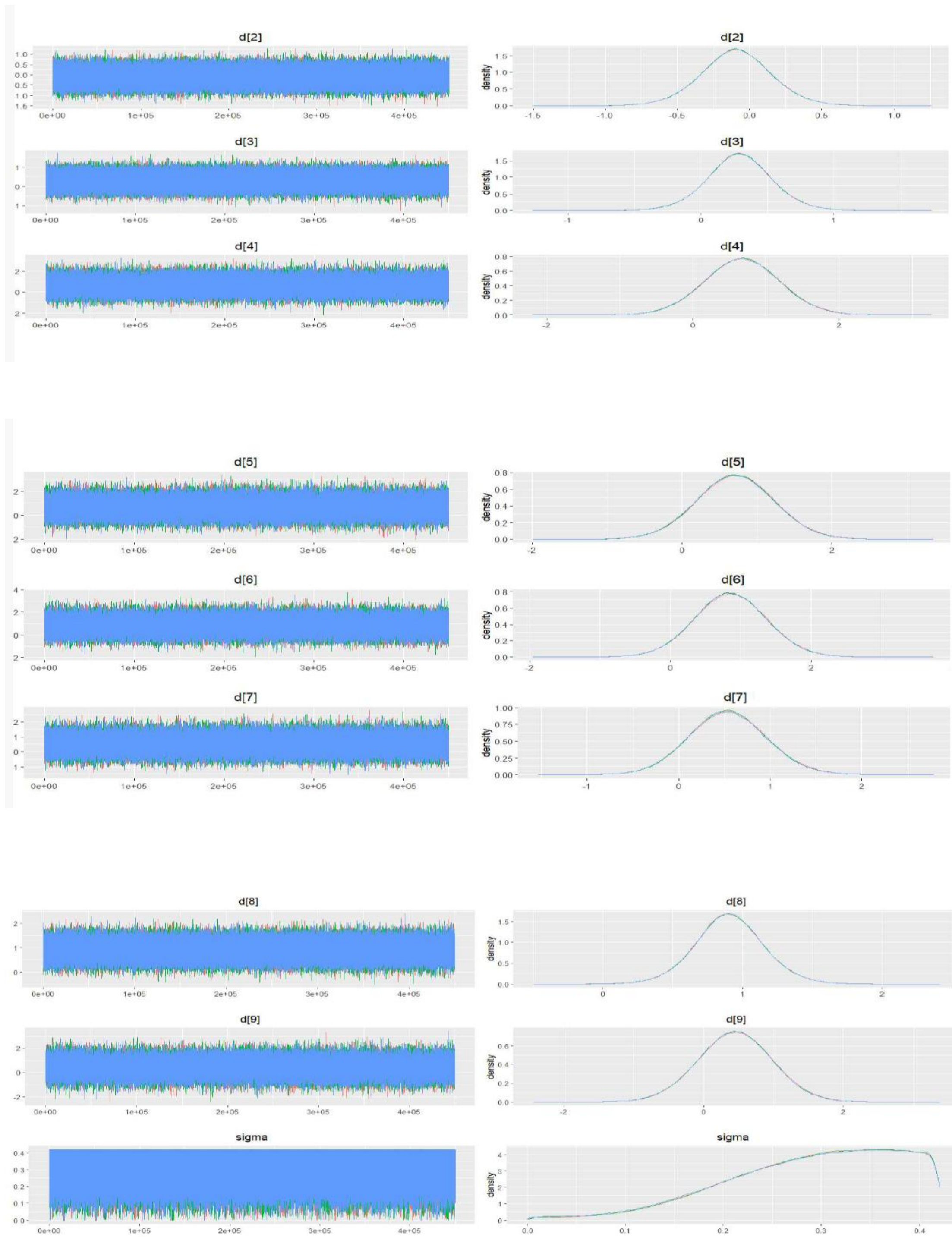

Supplementary Figure S1A: Density and trace plots for clinical remission

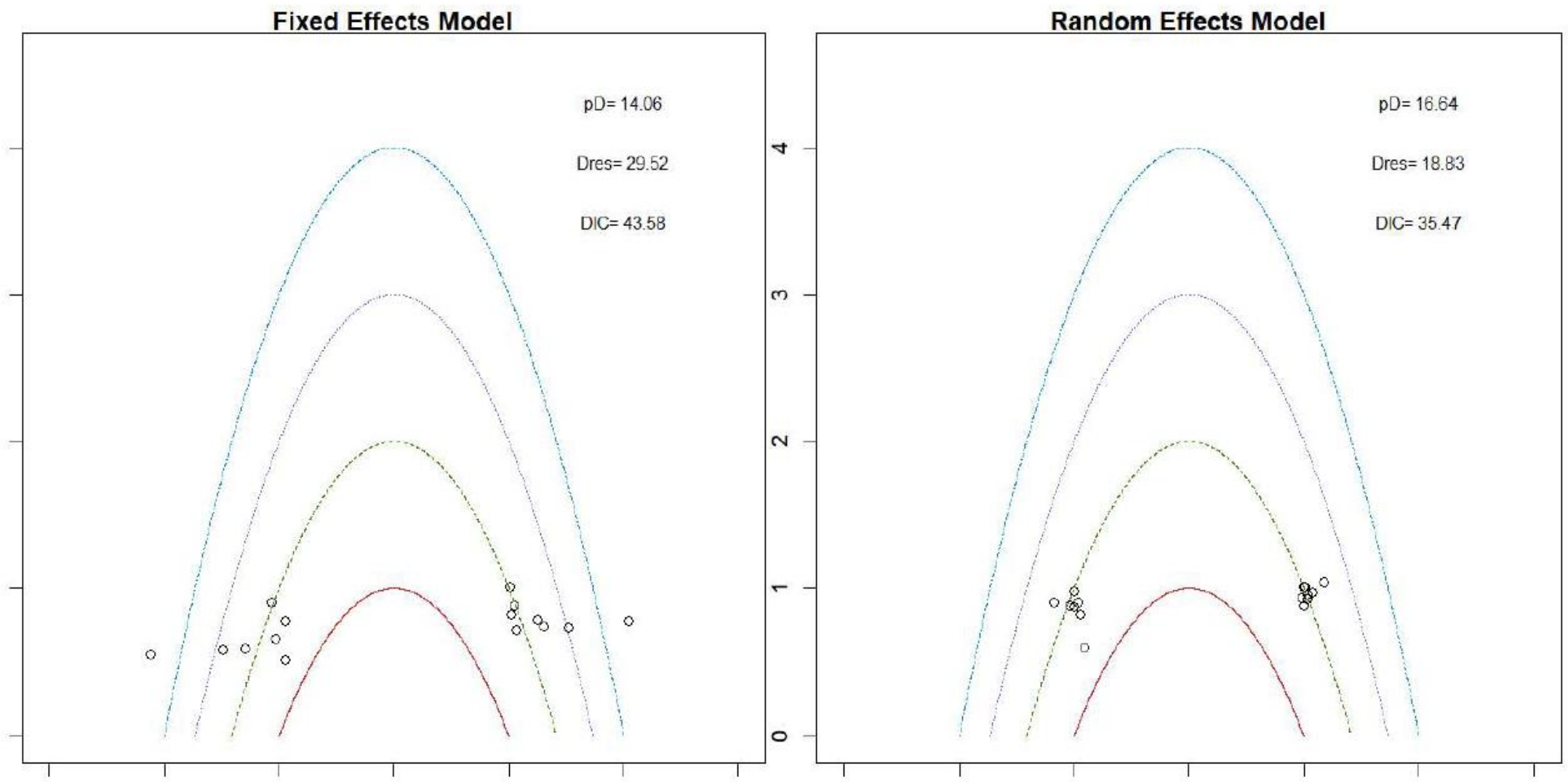

Supplementary Figure S1B: Rainbow plot for clinical remission

ENDOSCOPIC REMISSION:

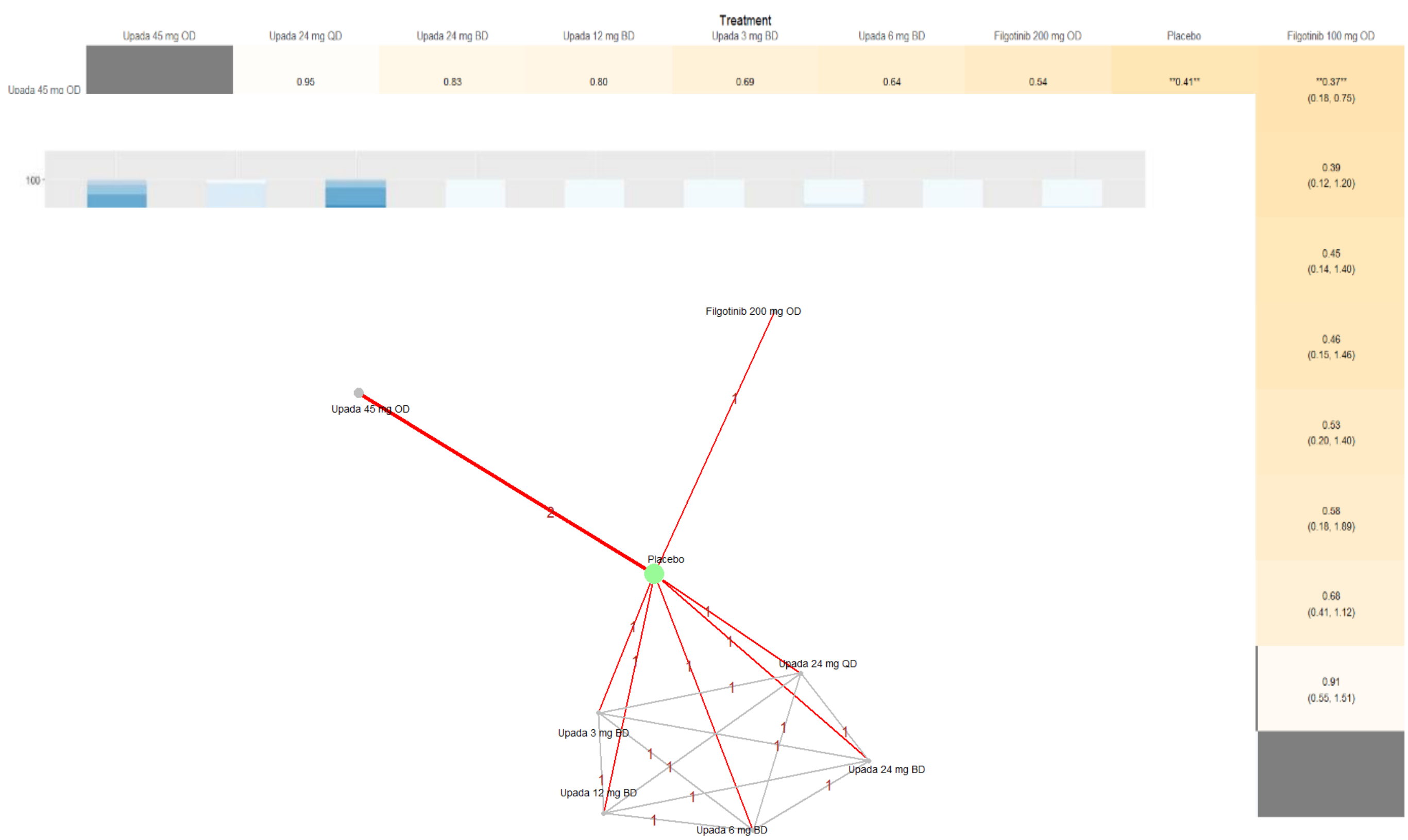

Supplementary Figure S2A: Network geometry plot for

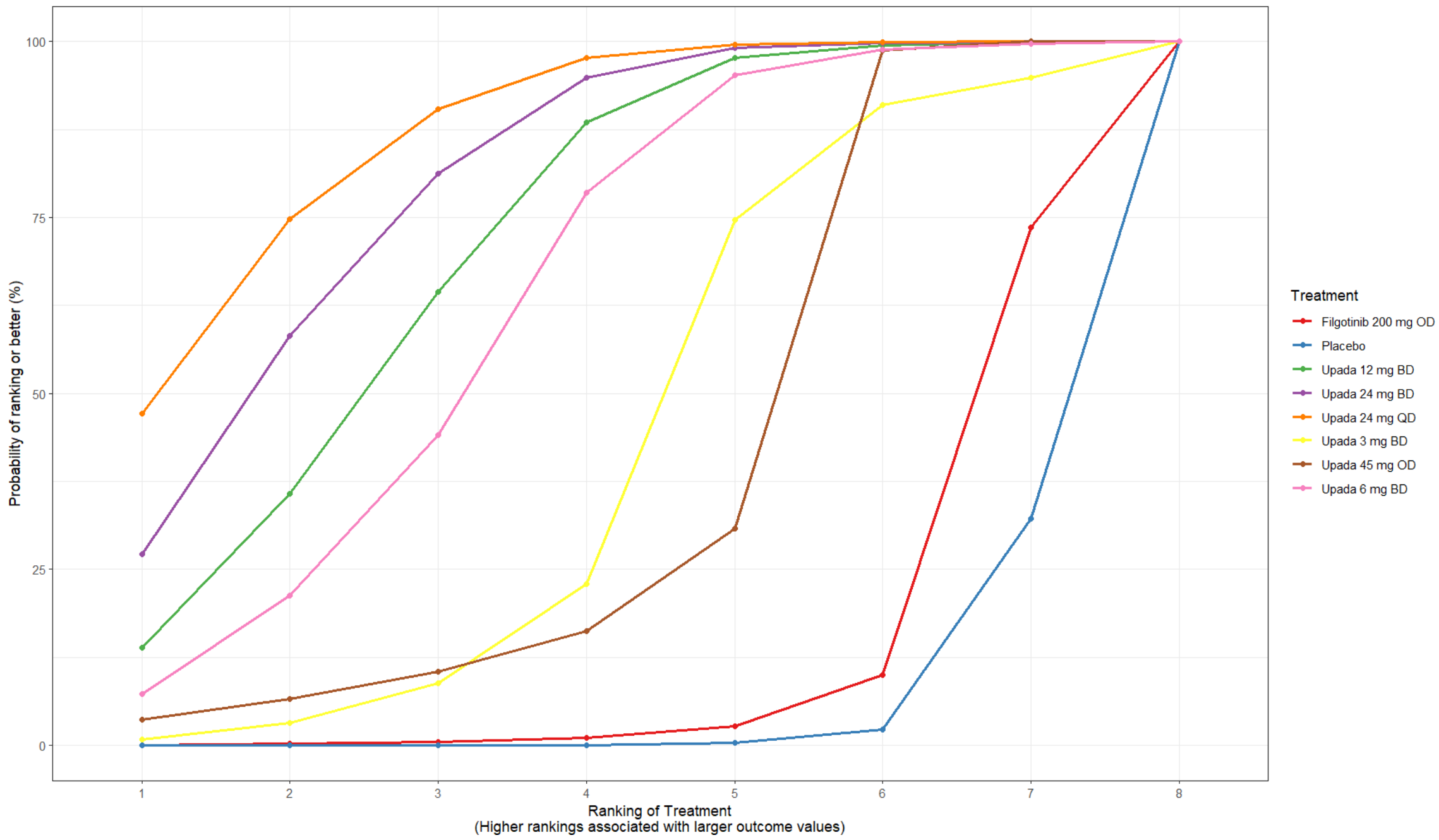

Supplementary Figure S2B: SUCRA Plot for endoscopic remission

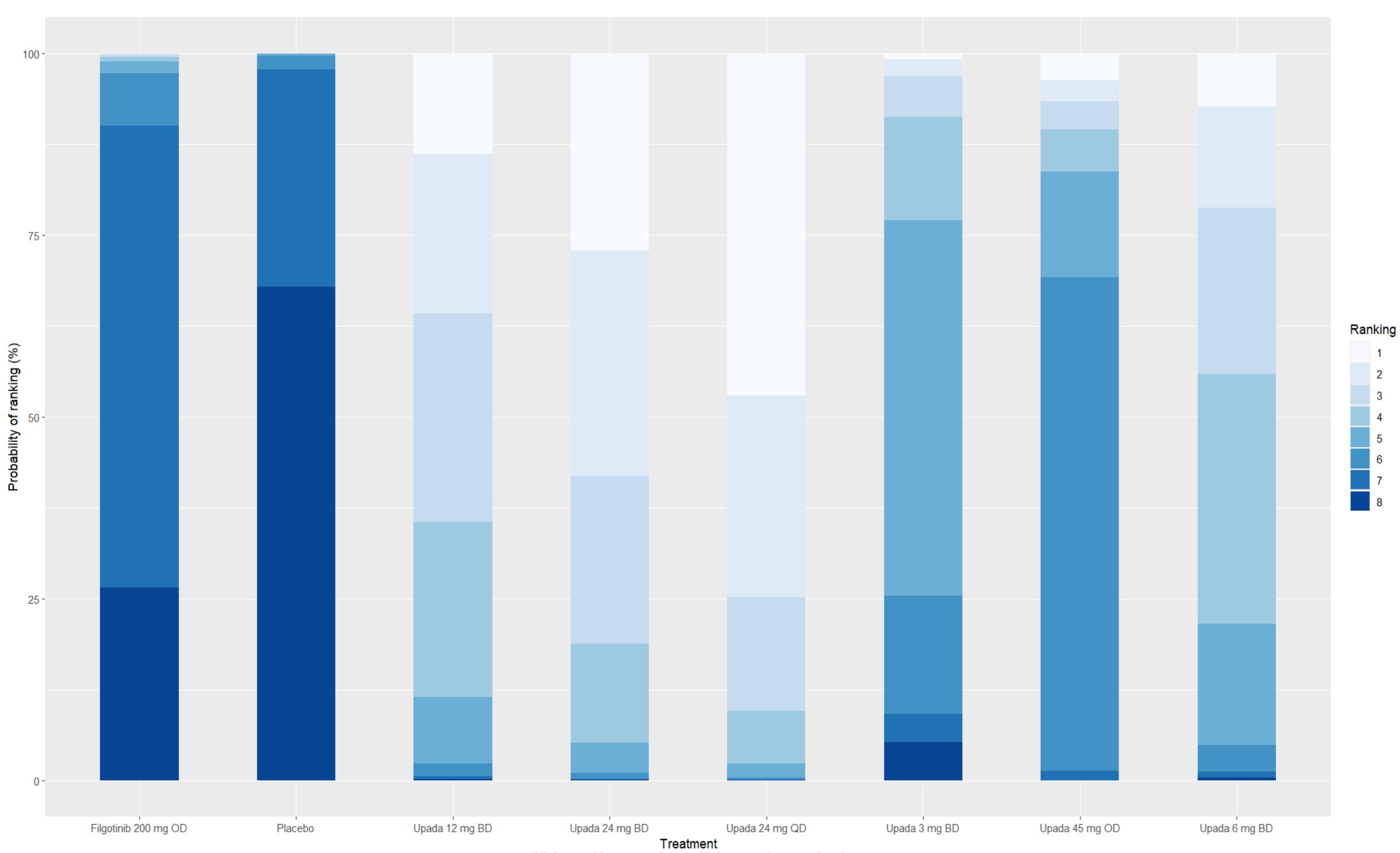

Supplementary Figure S2C: Rankogram for endoscopic remission

Treatment

Comparator	Upada 24 mg QD	Upada 24 mg BD	Upada 12 mg BD	Upada 6 mg BD	Upada 3 mg BD	Upada 45 mg OD	Filgotinib 200 mg OD	Placebo
Upada 24 mg QD		0.81 (0.20, 3.32)	0.63 (0.14, 2.61)	0.49 (0.10, 2.19)	0.21 (0.02, 1.16)	0.06 (0.00, 1.75)	**0.01** (0.00, 0.43)	**0.01** (0.00, 0.26)
Upada 24 mg BD	1.24 (0.30, 5.10)		0.77 (0.17, 3.43)	0.61 (0.12, 2.83)	0.25 (0.03, 1.47)	0.07 (0.00, 2.20)	**0.02** (0.00, 0.54)	**0.01** (0.00, 0.33)
Upada 12 mg BD	1.59 (0.38, 7.14)	1.29 (0.29, 5.94)		0.78 (0.15, 3.94)	0.33 (0.04, 2.01)	0.09 (0.00, 2.93)	**0.02** (0.00, 0.74)	**0.02** (0.00, 0.44)
Upada 6 mg BD	2.03 (0.46, 9.91)	1.64 (0.35, 8.40)	1.28 (0.25, 6.71)		0.42 (0.04, 2.76)	0.12 (0.00, 3.92)	**0.03** (0.00, 0.94)	**0.02** (0.00, 0.57)
Upada 3 mg BD	4.87 (0.86, 43.21)	3.94 (0.68, 35.86)	3.05 (0.50, 27.91)	2.40 (0.36, 22.93)		0.28 (0.00, 12.60)	0.06 (0.00, 3.09)	0.05 (0.00, 1.92)
Upada 45 mg OD	17.45 (0.57, 24205.70)	14.19 (0.46, 19017.08)	10.91 (0.34, 14786.32)	8.66 (0.26, 12956.78)	3.57 (0.08, 5100.31)		**0.23** (0.06, 0.87)	**0.17** (0.07, 0.36)
Filgotinib 200 mg OD	**77.86** (2.31, 107675.18)	**62.83** (1.86, 83159.16)	**48.94** (1.36, 63850.48)	**38.32** (1.06, 53664.56)	15.78 (0.32, 21822.82)	**4.40** (1.15, 17.59)		0.75 (0.24, 2.20)
Placebo	**103.67** (3.85, 138064.75)	**84.50** (3.05, 107504.71)	**64.50** (2.26, 82004.59)	**51.15** (1.75, 71852.56)	21.00 (0.52, 28484.55)	**5.89** (2.79, 13.86)	1.33 (0.45, 4.20)	

Supplementary Figure S2D: League table for endoscopic remission

CLINICAL RESPONSE:

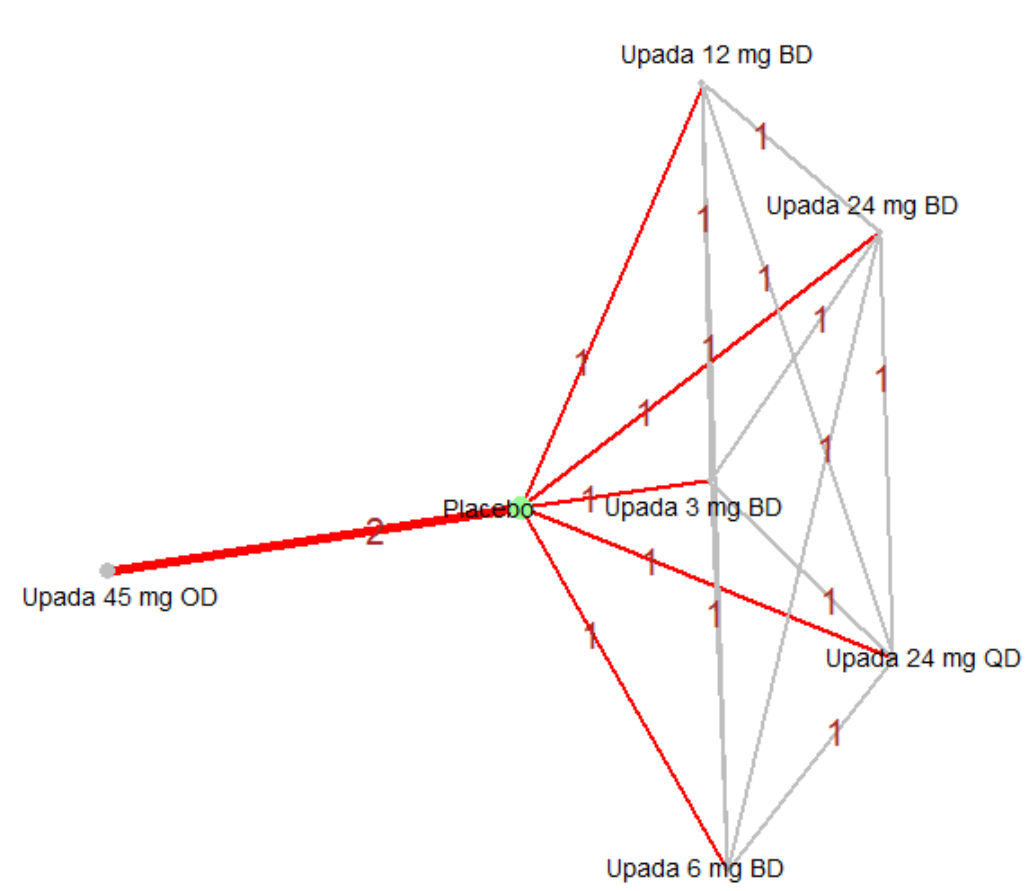

Supplementary Figure S3A: Network geometry plot for clinical response

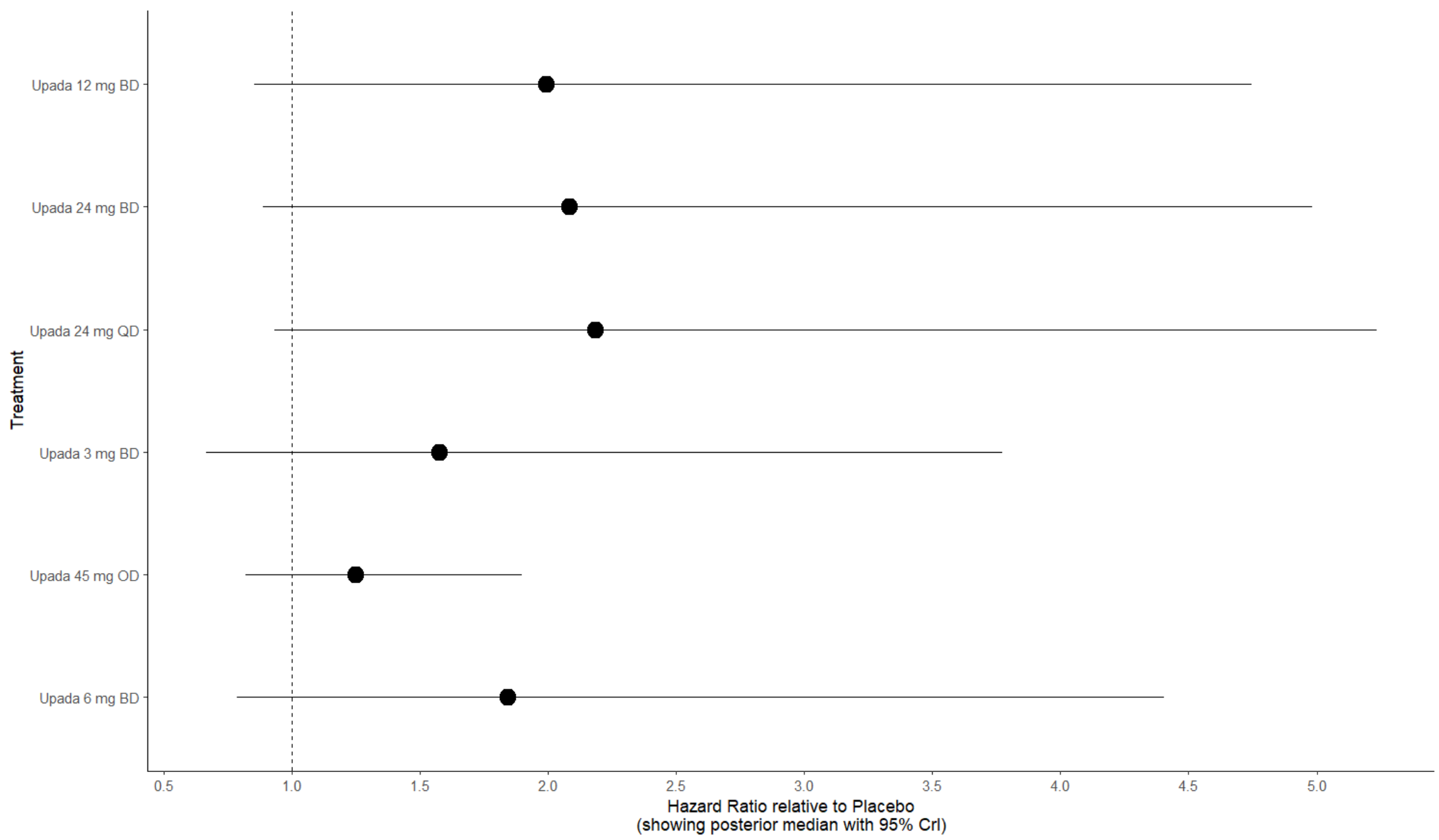

Supplementary Figure S3B: Forest plot for clinical response

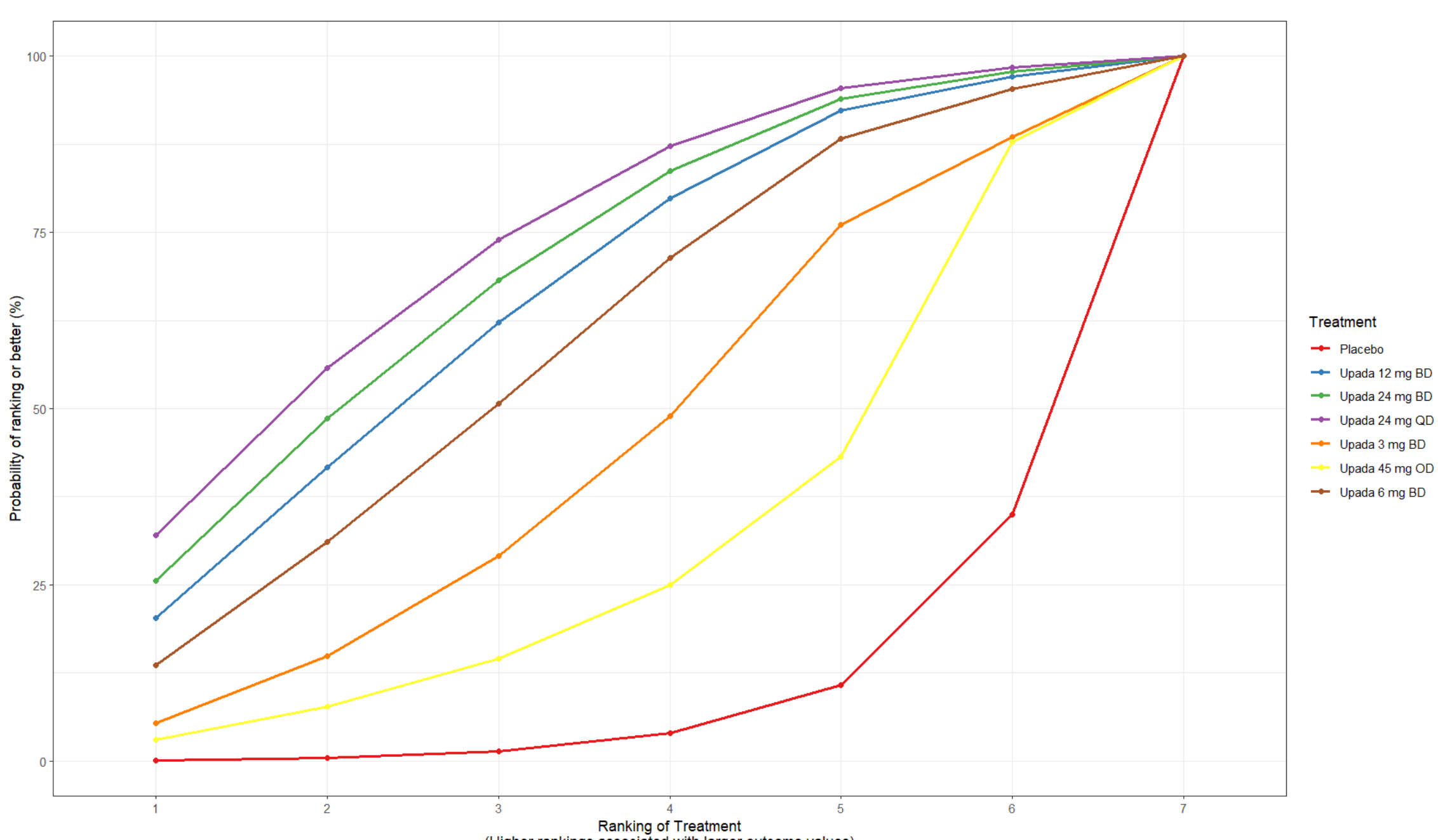

Supplementary Figure S3C: SUCRA plot for clinical response

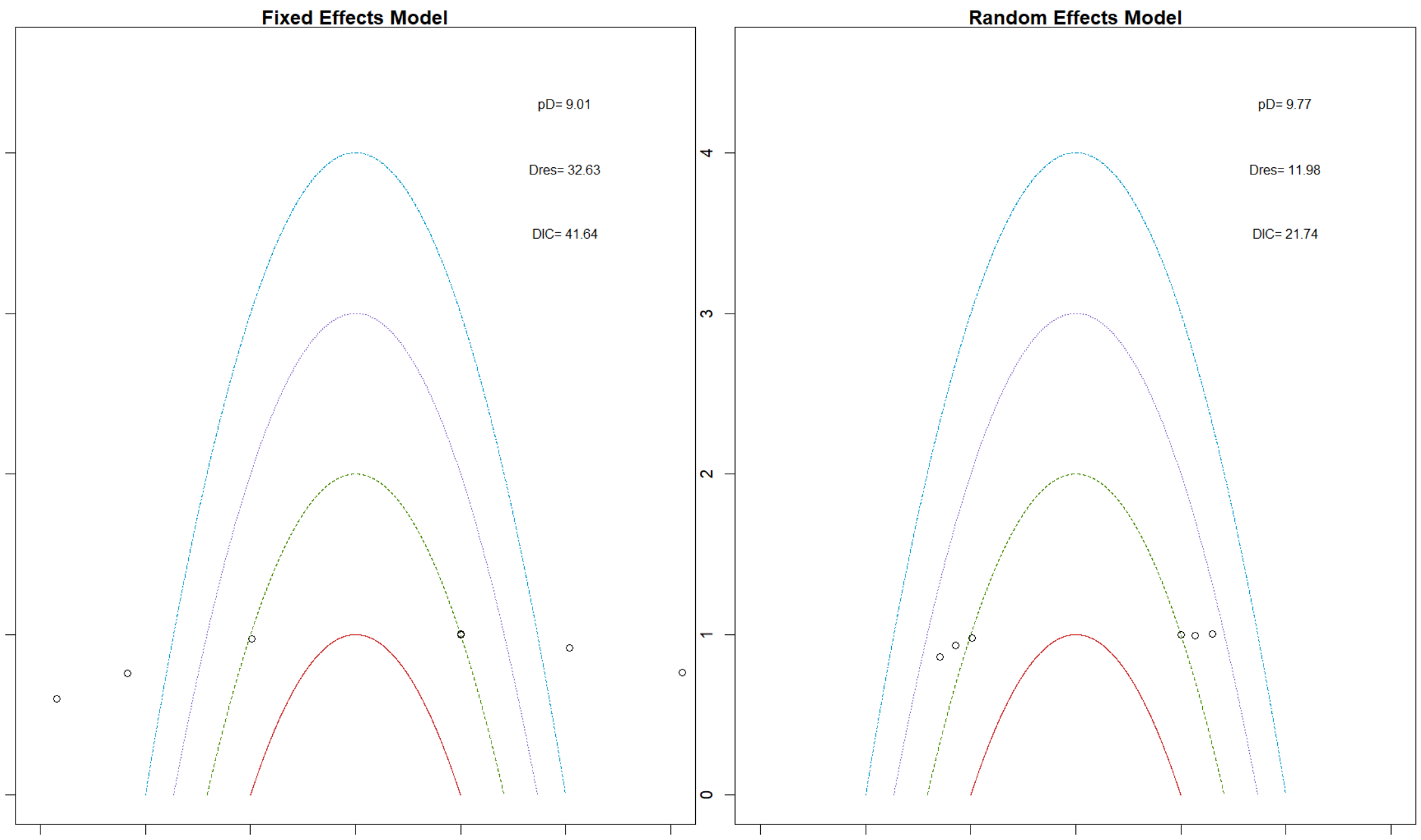

Supplementary Figure S3D: Rainbow plot for clinical response

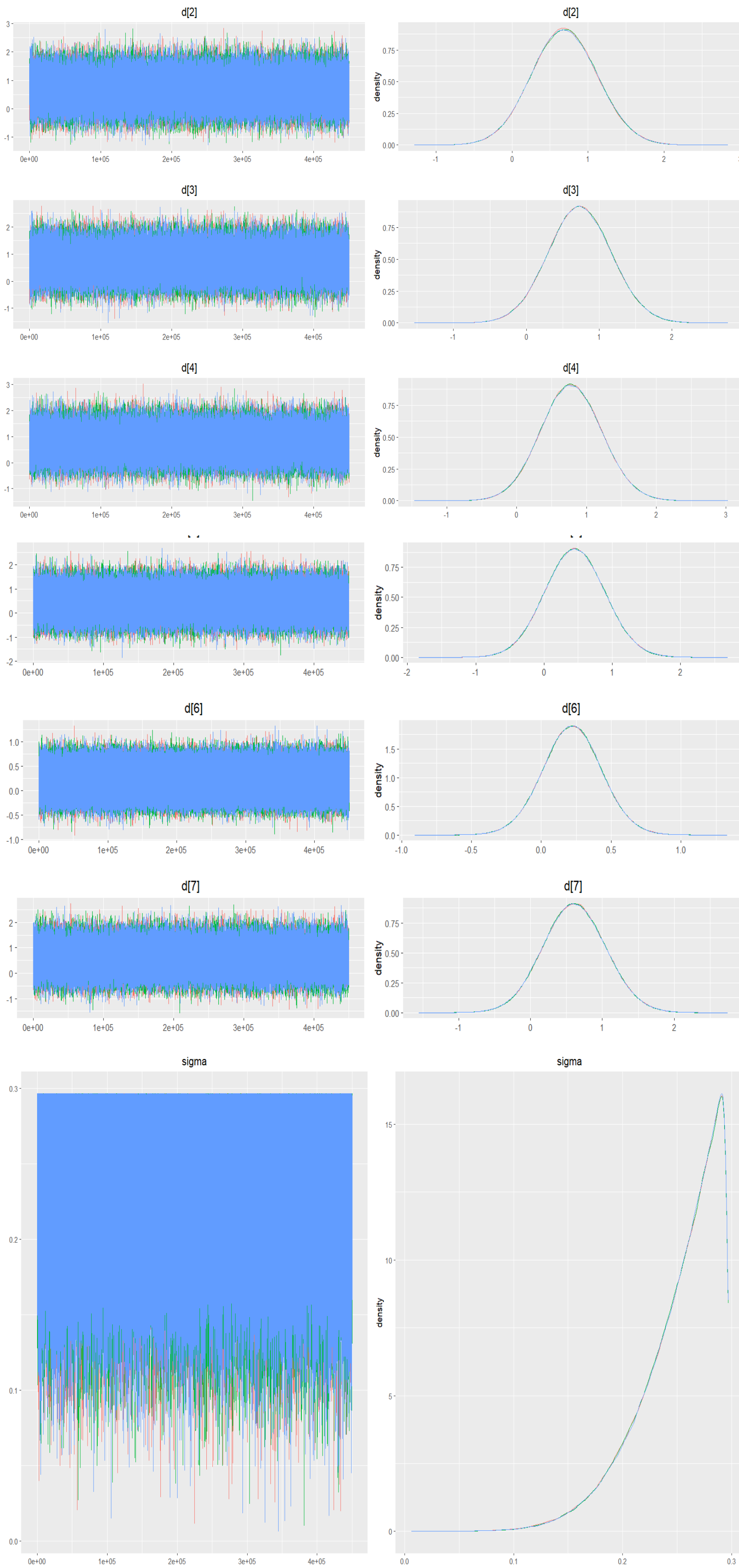

Supplementary Figure S3E: Density and trace plots for clinical response

Comparator \ Treatment	Upada 24 mg QD	Upada 24 mg BD	Upada 12 mg BD	Upada 6 mg BD	Upada 3 mg BD	Upada 45 mg OD	Placebo
Upada 24 mg QD		0.95 (0.43, 2.14)	0.91 (0.41, 2.04)	0.84 (0.37, 1.90)	0.72 (0.32, 1.62)	0.57 (0.22, 1.48)	0.46 (0.19, 1.07)
Upada 24 mg BD	1.05 (0.47, 2.35)		0.96 (0.43, 2.14)	0.88 (0.39, 1.98)	0.76 (0.33, 1.70)	0.60 (0.23, 1.55)	0.48 (0.20, 1.12)
Upada 12 mg BD	1.10 (0.49, 2.46)	1.05 (0.47, 2.34)		0.93 (0.41, 2.08)	0.79 (0.35, 1.78)	0.63 (0.24, 1.62)	0.50 (0.21, 1.17)
Upada 6 mg BD	1.19 (0.53, 2.68)	1.13 (0.50, 2.55)	1.08 (0.48, 2.43)		0.85 (0.38, 1.94)	0.68 (0.26, 1.75)	0.54 (0.23, 1.27)
Upada 3 mg BD	1.39 (0.62, 3.14)	1.32 (0.59, 3.00)	1.27 (0.56, 2.86)	1.17 (0.52, 2.65)		0.79 (0.30, 2.07)	0.64 (0.26, 1.50)
Upada 45 mg OD	1.75 (0.68, 4.62)	1.67 (0.65, 4.40)	1.60 (0.62, 4.19)	1.48 (0.57, 3.88)	1.26 (0.48, 3.33)		0.80 (0.53, 1.22)
Placebo	2.18 (0.93, 5.23)	2.08 (0.89, 4.98)	1.99 (0.85, 4.75)	1.84 (0.79, 4.40)	1.57 (0.67, 3.77)	1.25 (0.82, 1.90)	

Supplementary Figure S3F: League table for clinical response

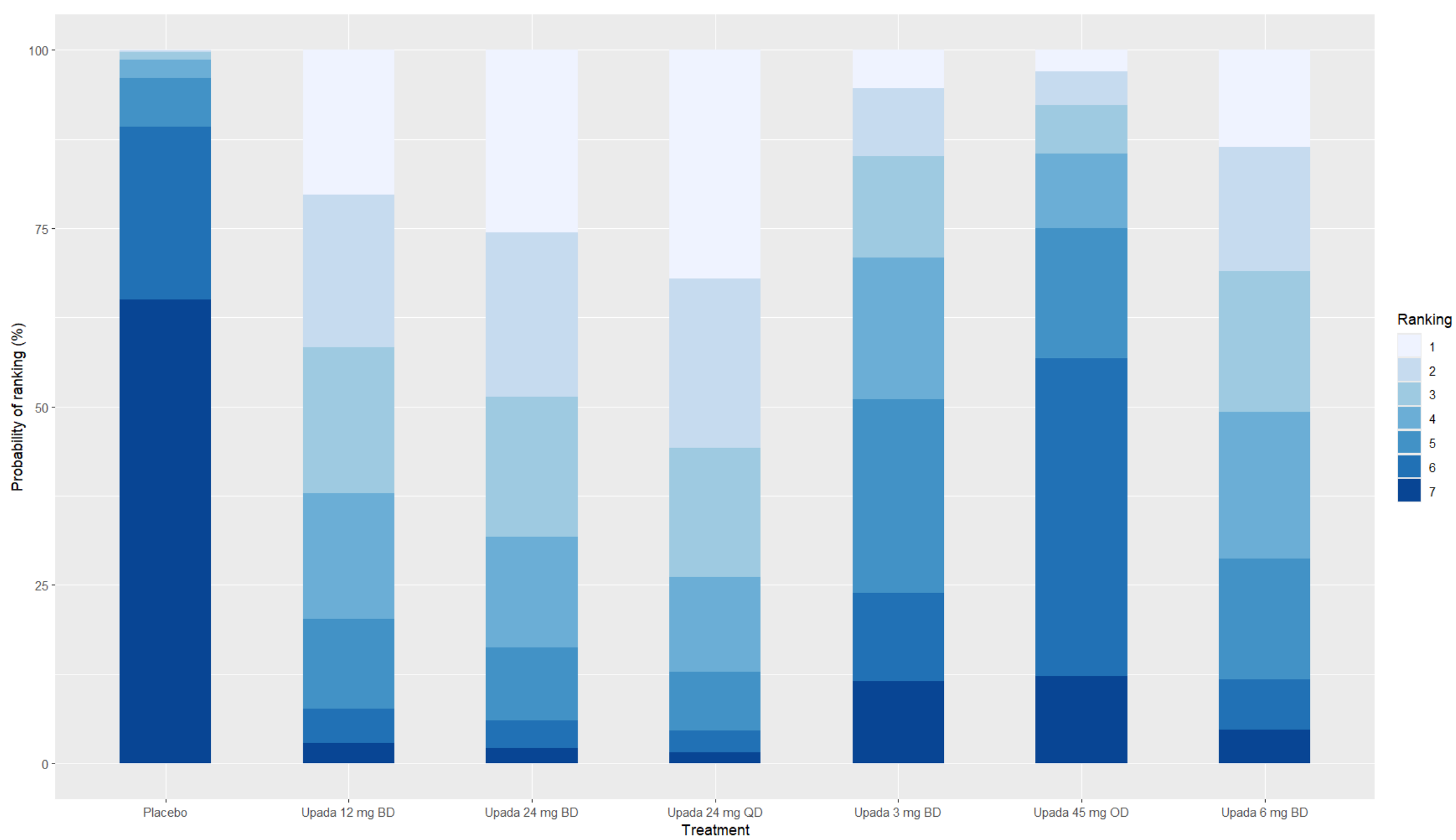

Supplementary Figure S3G: Rankogram for clinical response

ENDOSCOPIC RESPONSE:

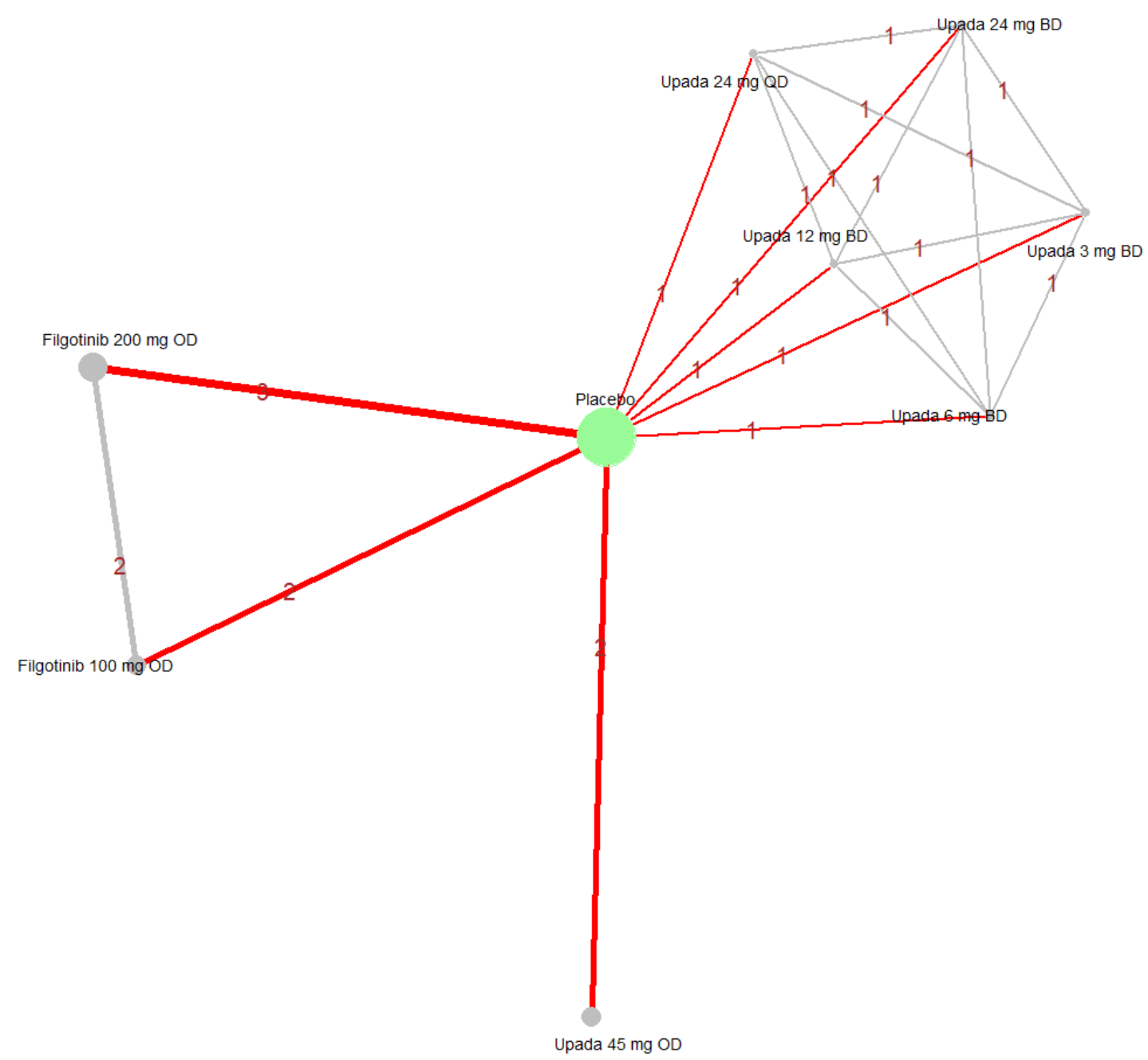

Supplementary Figure S4A: Network geometry plot for endoscopic response

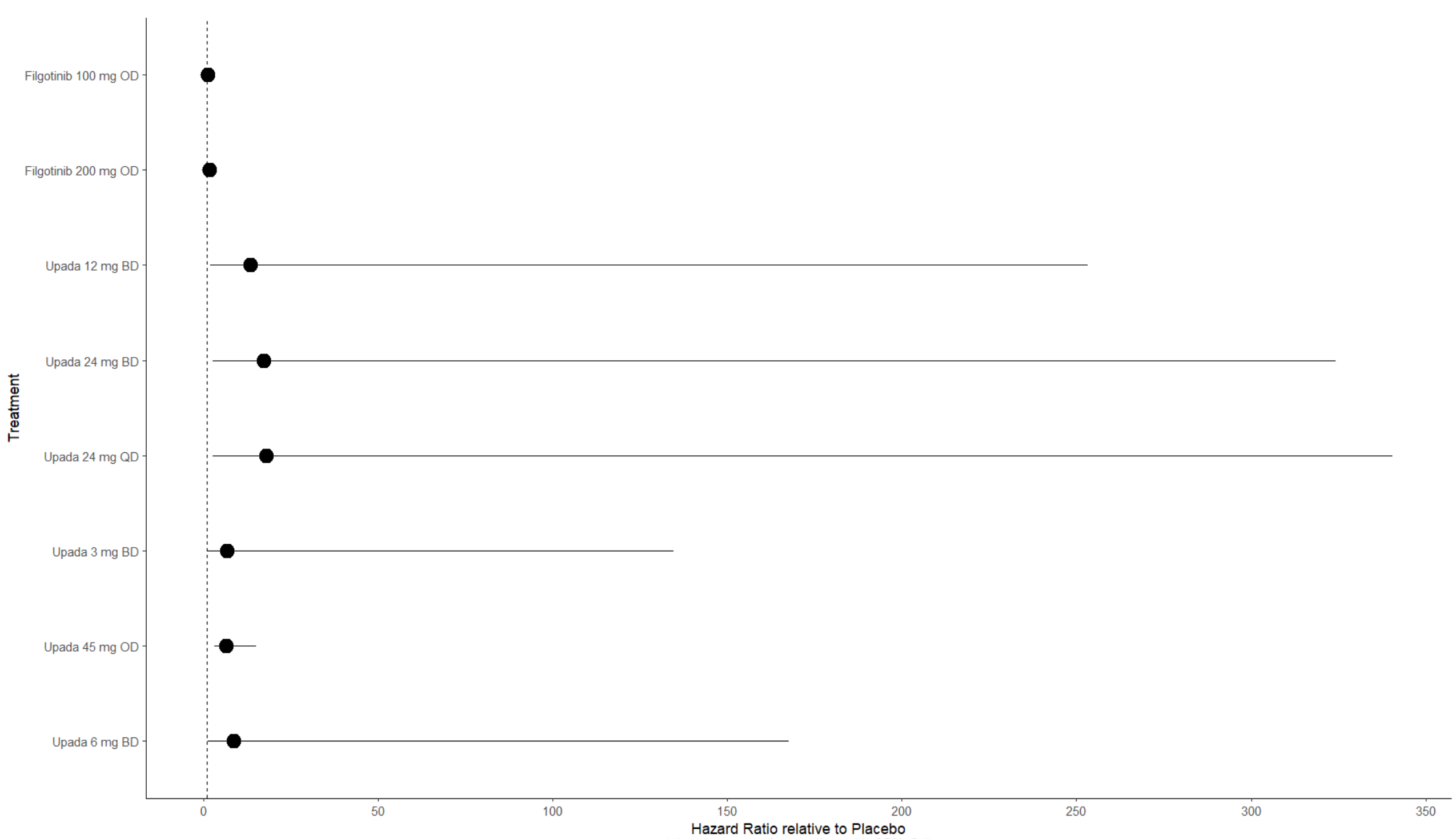

Supplementary Figure S4B: Forest plot for endoscopic response

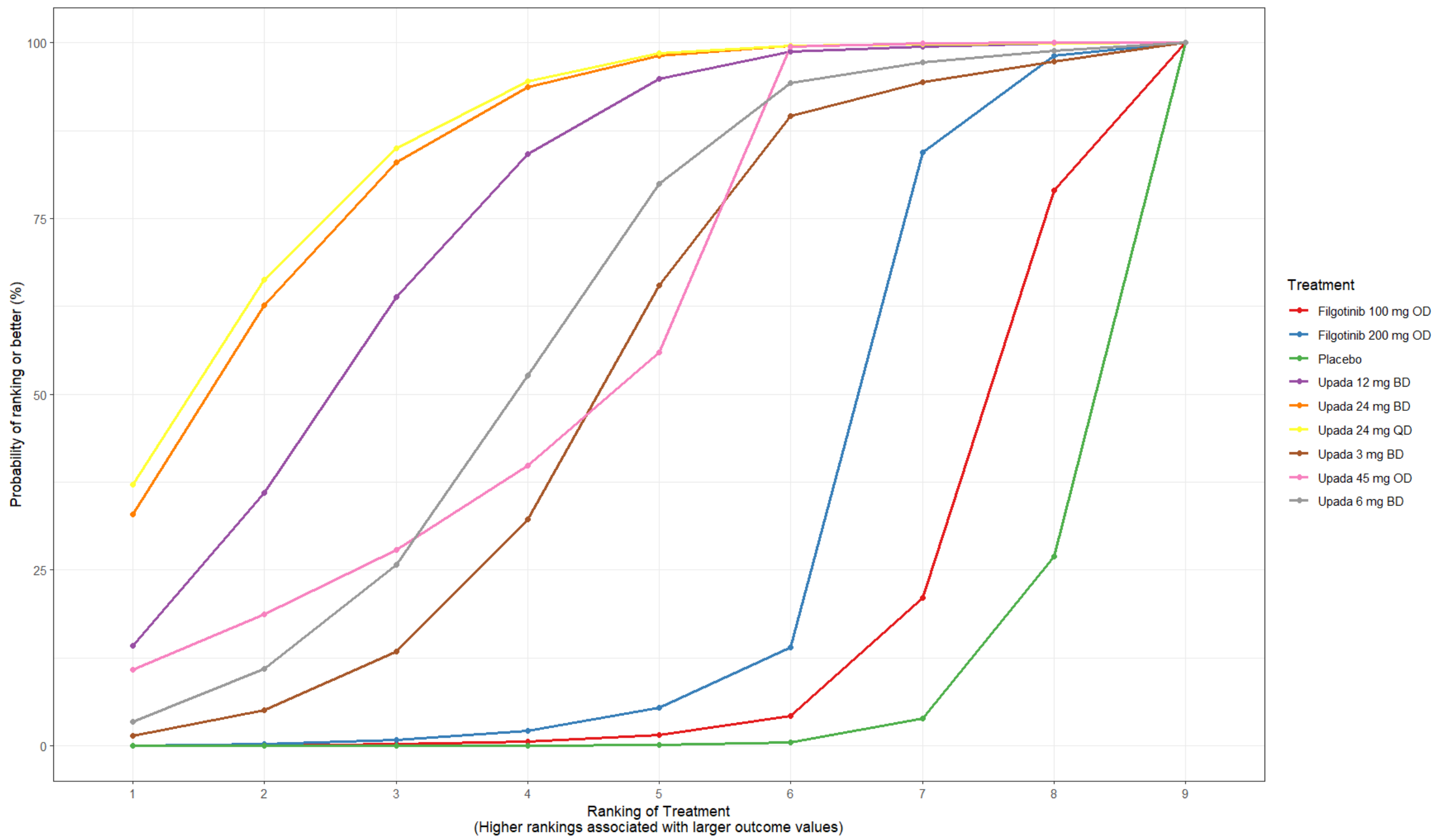

Supplementary Figure S4C: SUCRA plot for endoscopic response

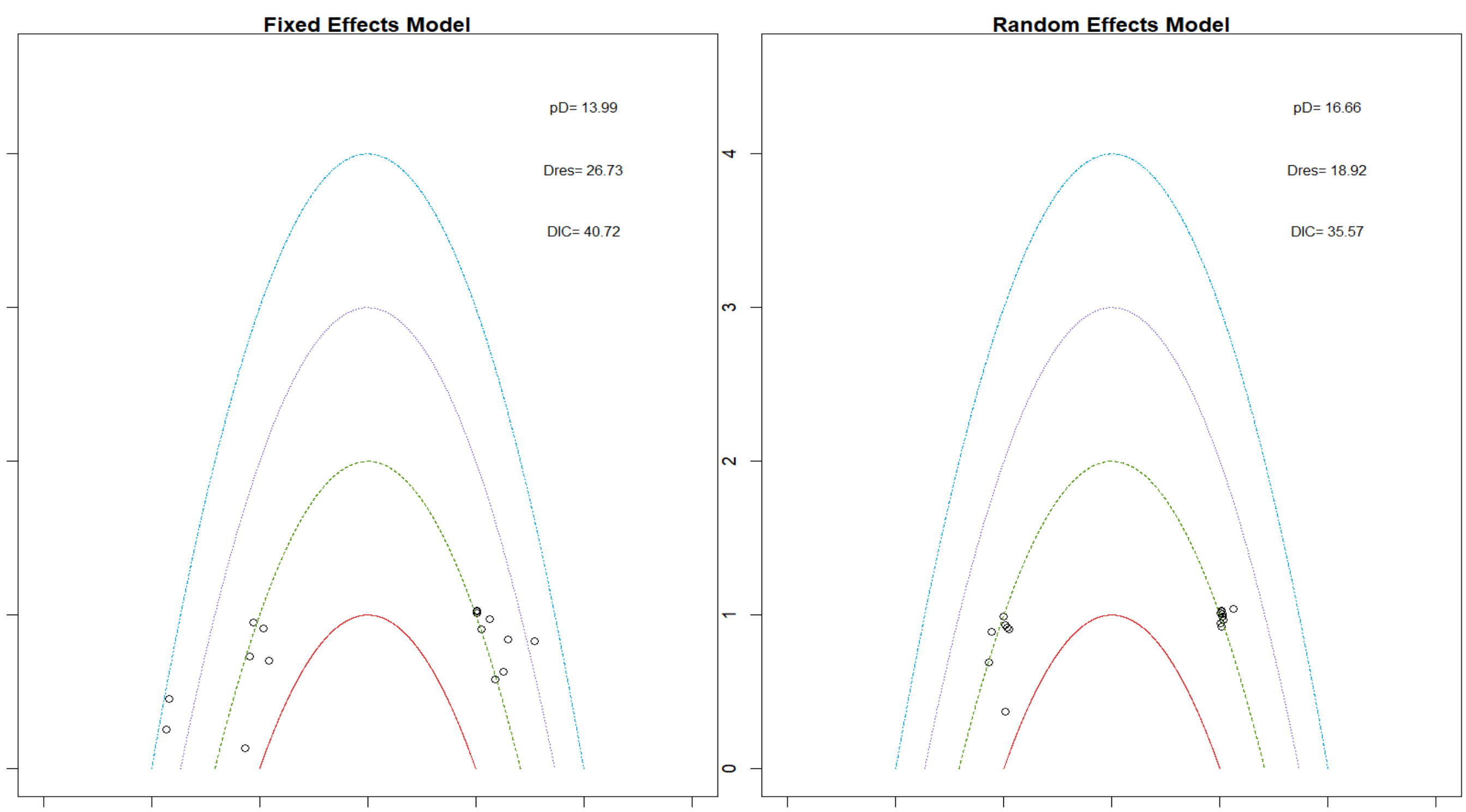

Supplementary Figure S4D: Rainbow plot for endoscopic response

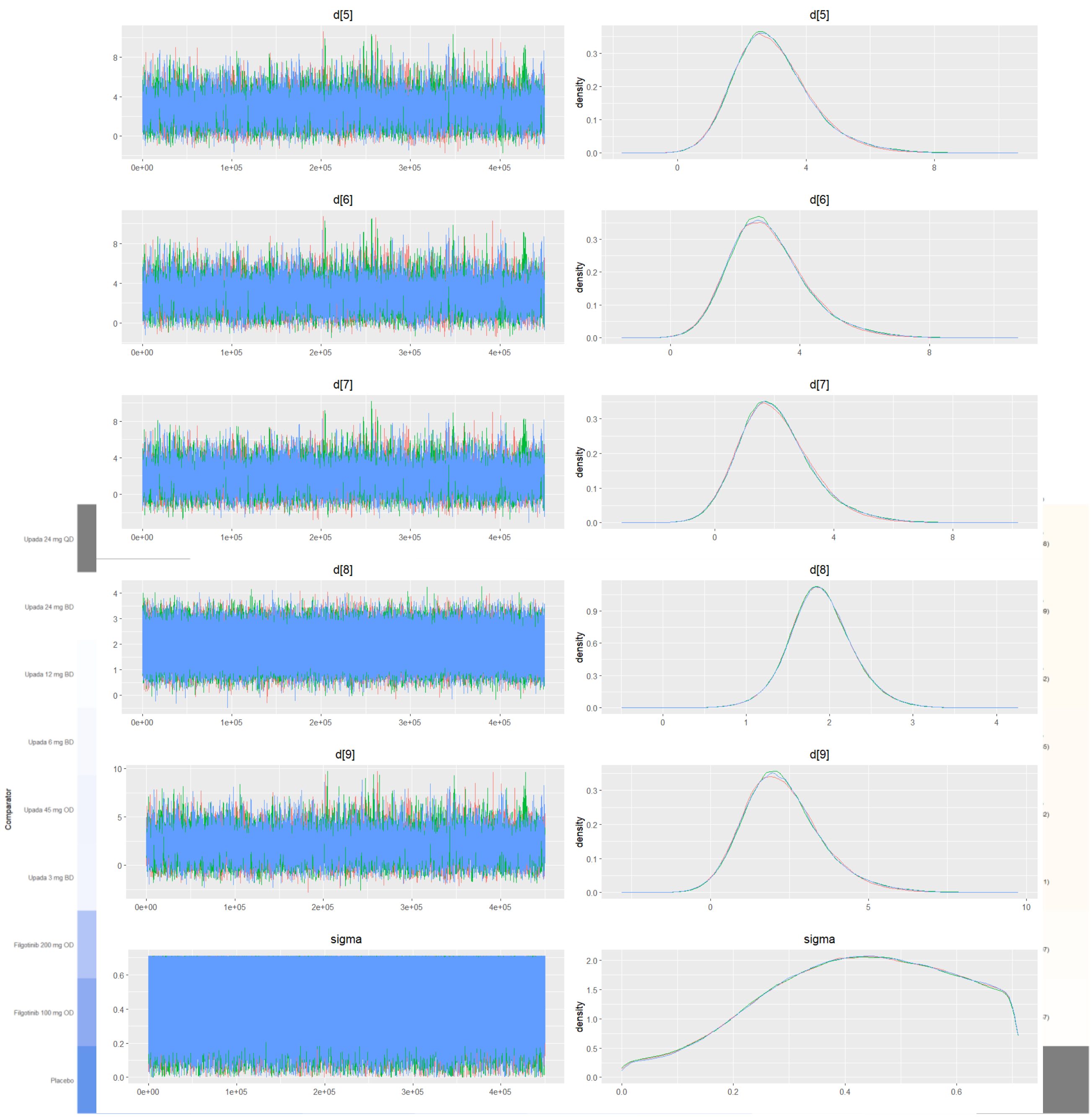

Supplementary Figure S4E: Density and trace plot for endoscopic response

TOTAL ADVERSE EVENTS:

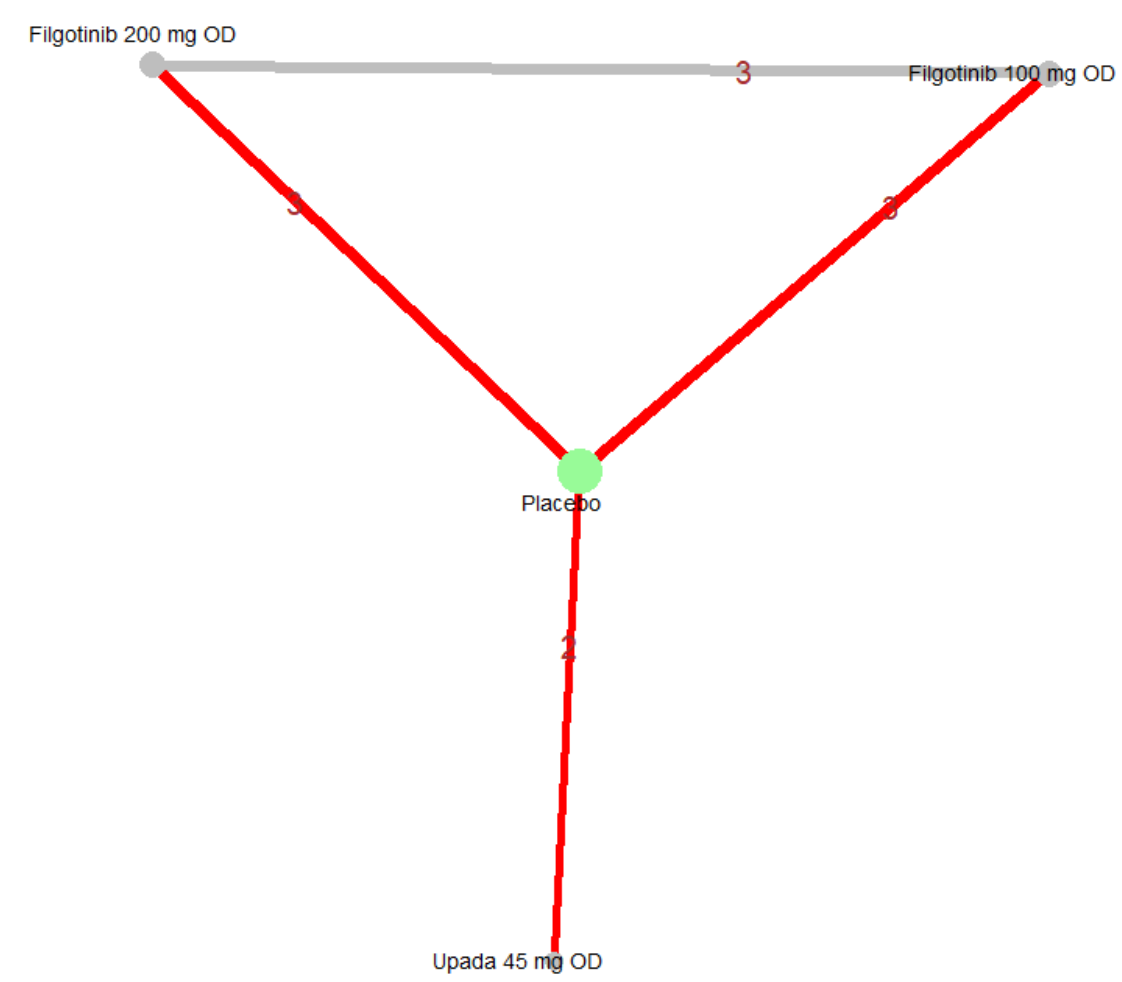

Supplementary Figure S5A: Network geometry plot for total adverse events

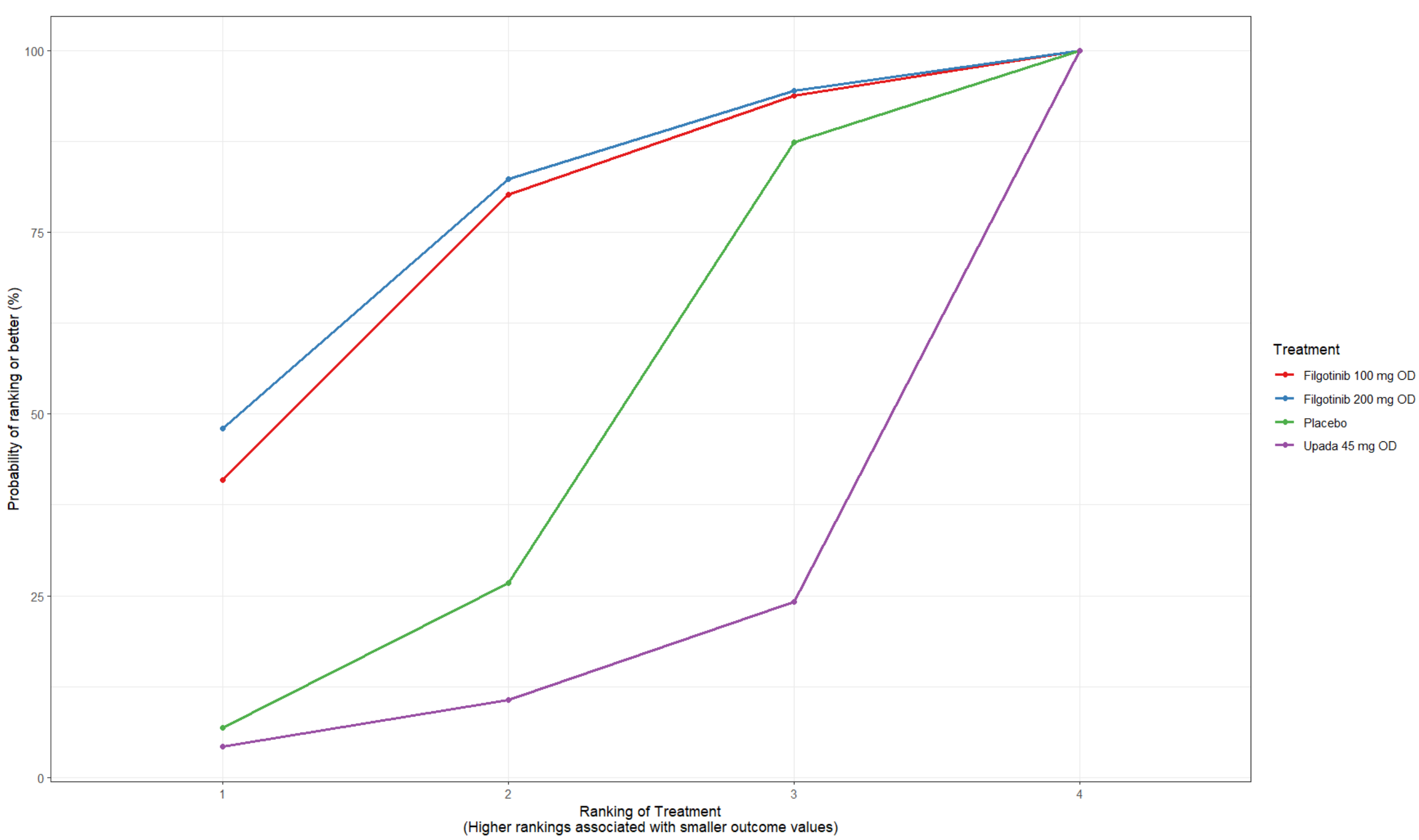

Supplementary Figure S5B: SUCRA plot for total adverse events

Comparator \ Treatment	Filgotinib 200 mg OD	Filgotinib 100 mg OD	Placebo	Upada 45 mg OD
Filgotinib 200 mg OD		1.01<br>(0.85, 1.20)	1.08<br>(0.91, 1.28)	1.17<br>(0.92, 1.48)
Filgotinib 100 mg OD	0.99<br>(0.84, 1.18)		1.07<br>(0.91, 1.26)	1.16<br>(0.92, 1.46)
Placebo	0.93<br>(0.78, 1.10)	0.93<br>(0.79, 1.10)		1.08<br>(0.92, 1.28)
Upada 45 mg OD	0.86<br>(0.67, 1.09)	0.86<br>(0.68, 1.09)	0.93<br>(0.78, 1.09)	

Supplementary Figure S5C: League table for total adverse events

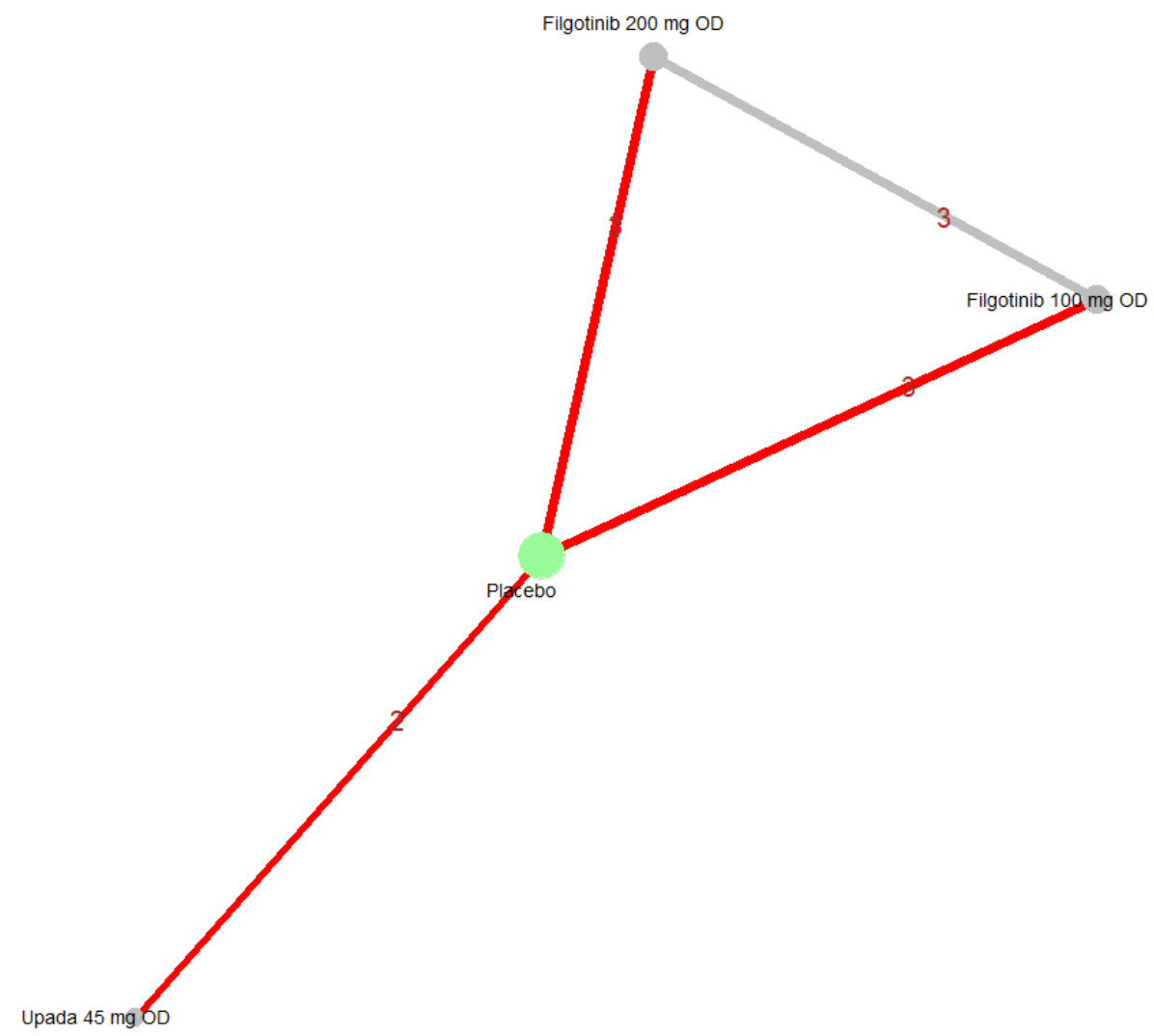

Supplementary Figure S6A: Network geometry plot for serious adverse events

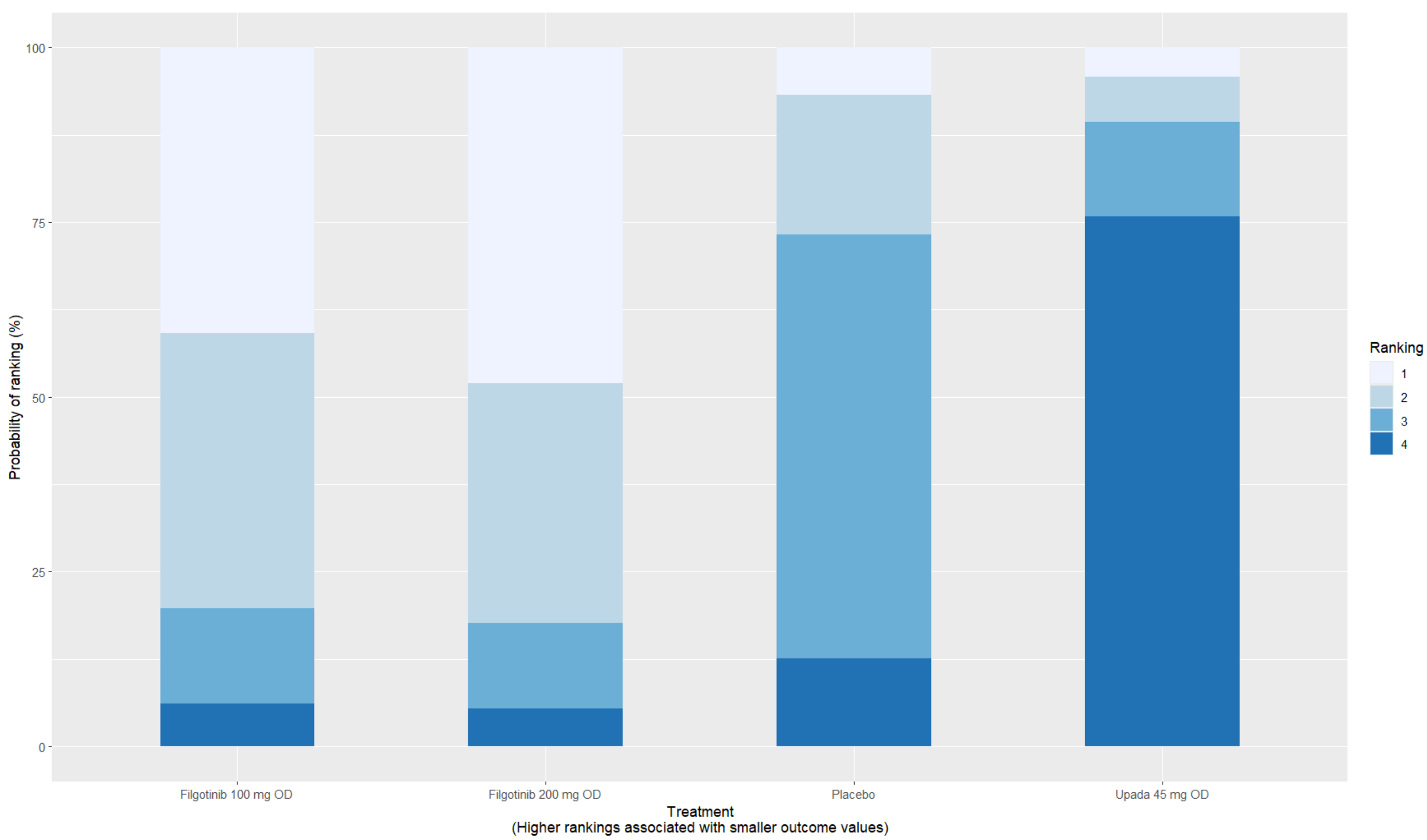

Supplementary Figure S5D: Rankogram for total adverse events

SERIOUS ADVERSE EVENTS:

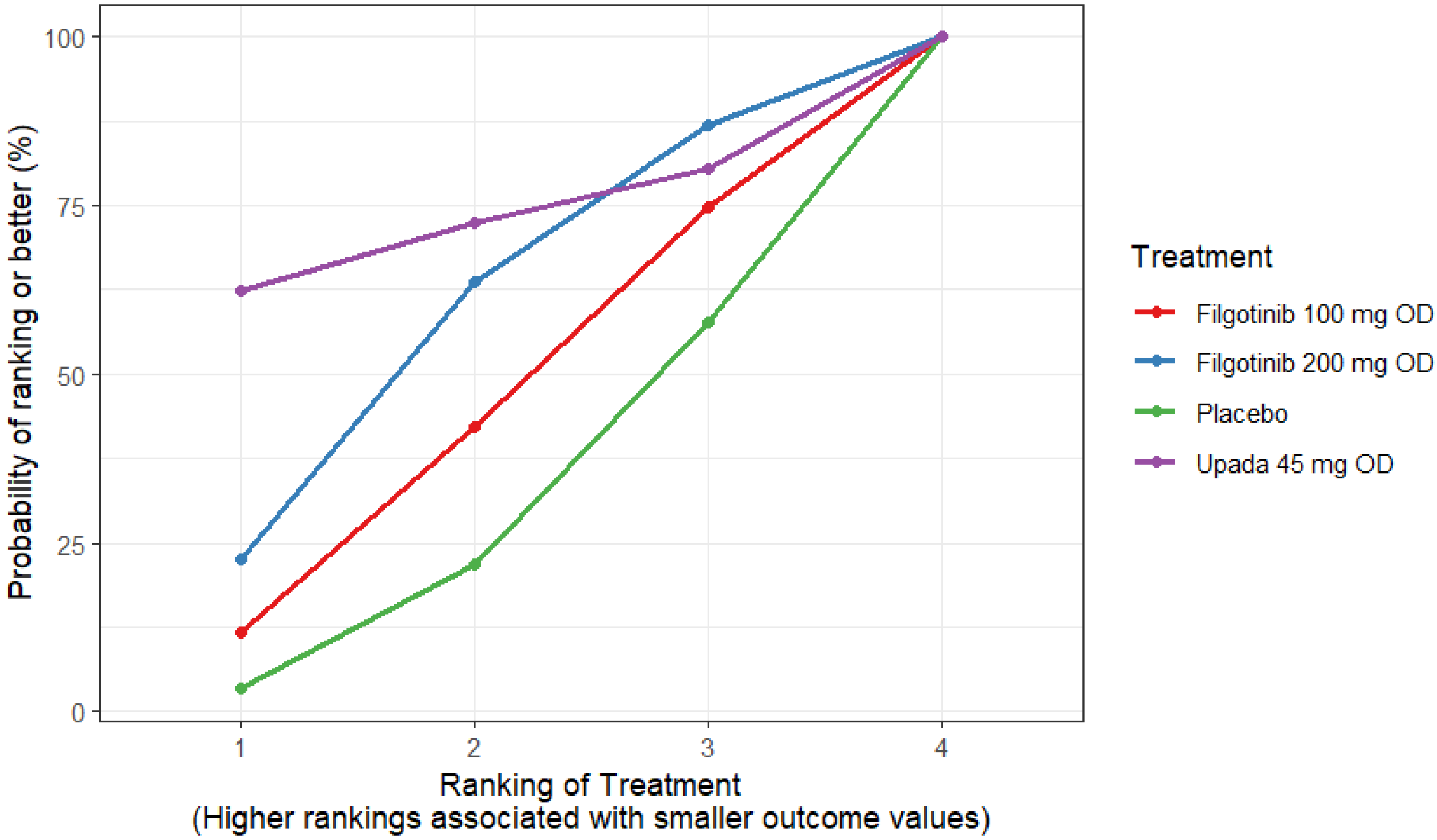

Supplementary Figure S6B: SUCRA plot for serious adverse events

GI PERFORATION:

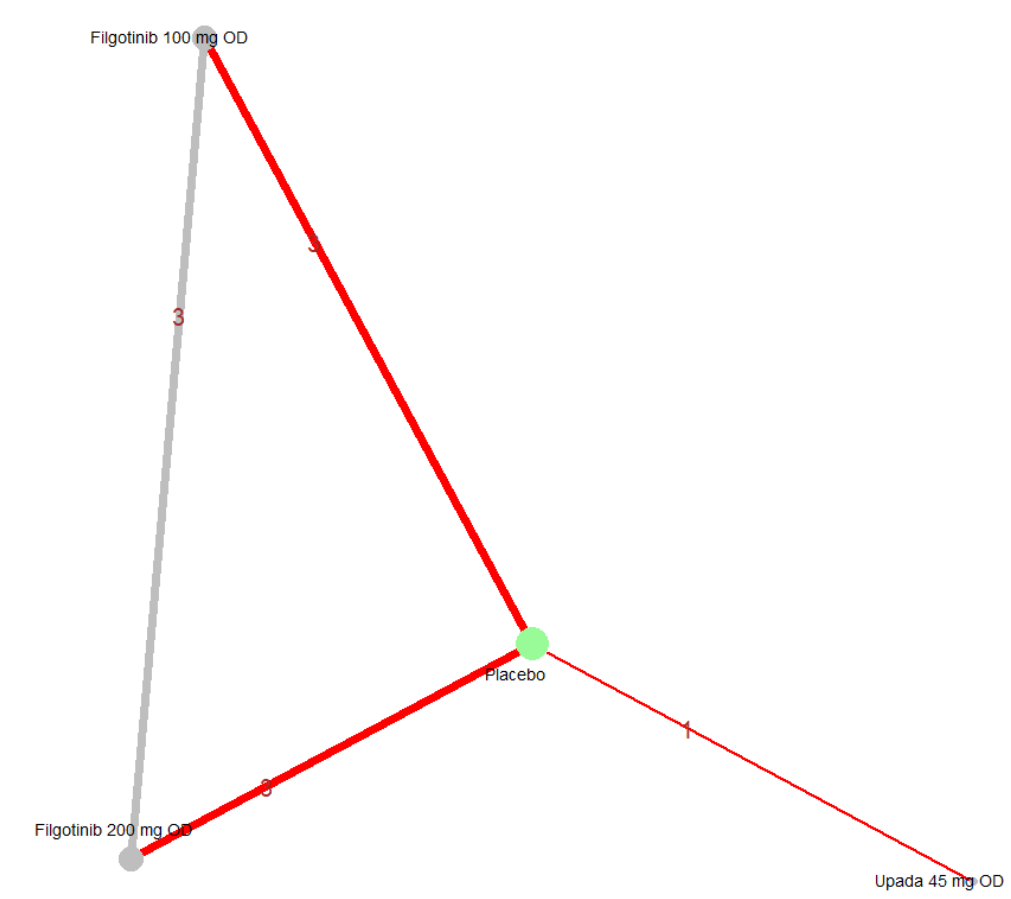

Supplementary Figure S7A: Network geometry plot for GI perforation

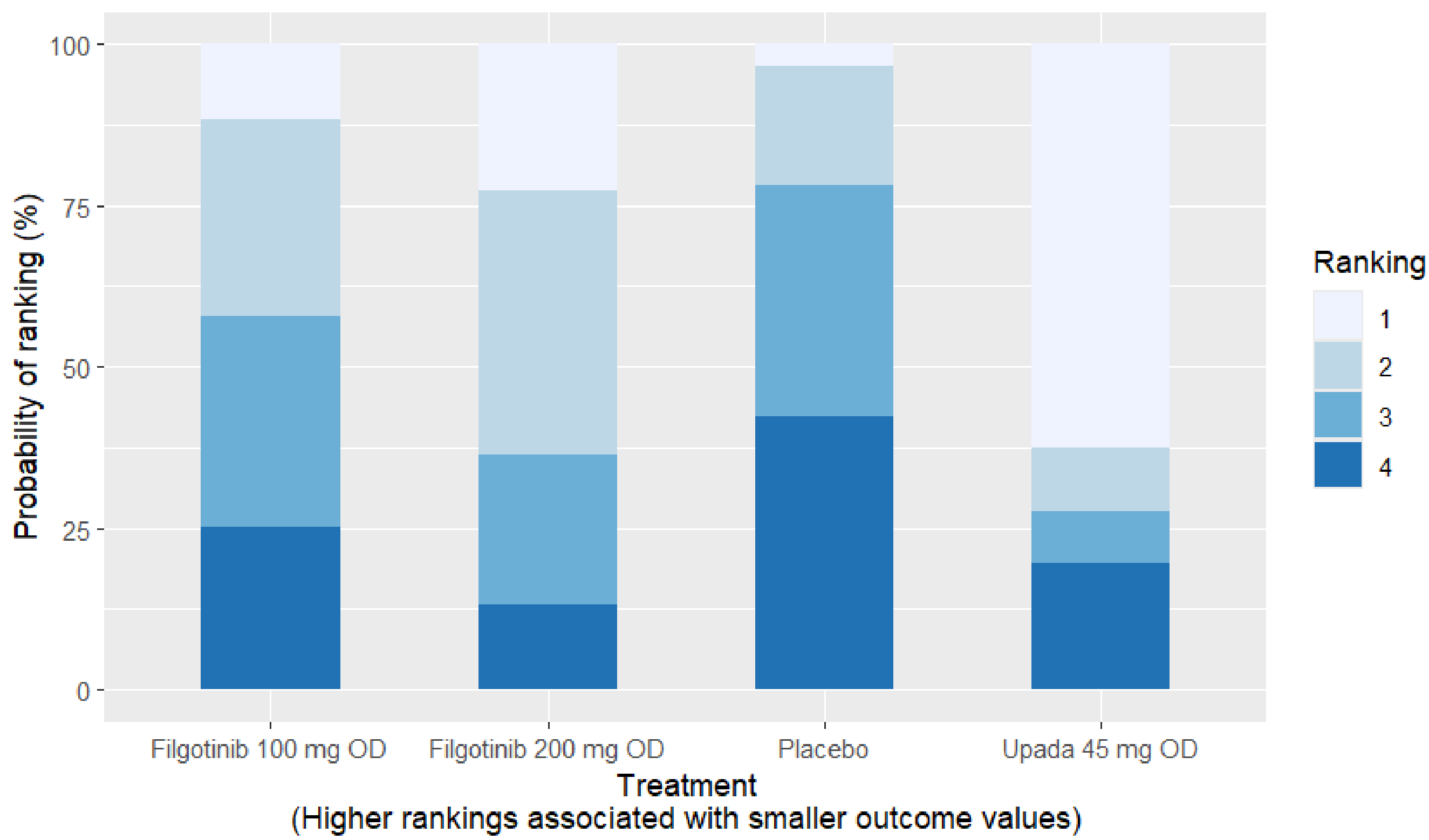

Supplementary Figure S7B: Rankogram for serious adverse events

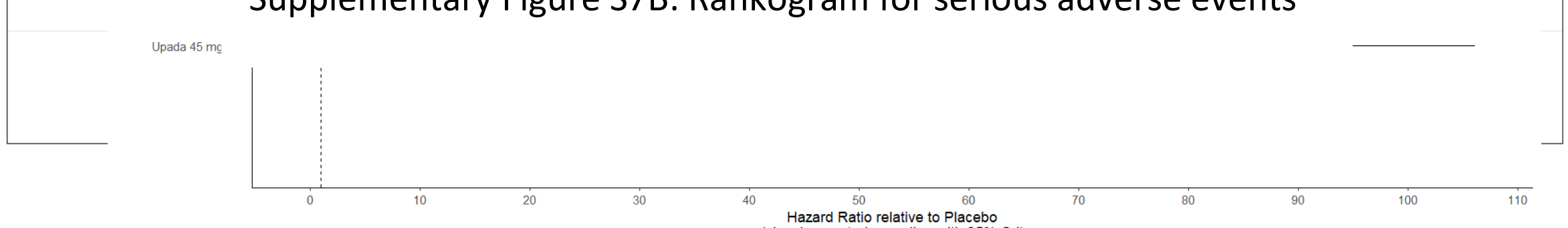

Supplementary Figure S7C: Forest plot for GI perforation

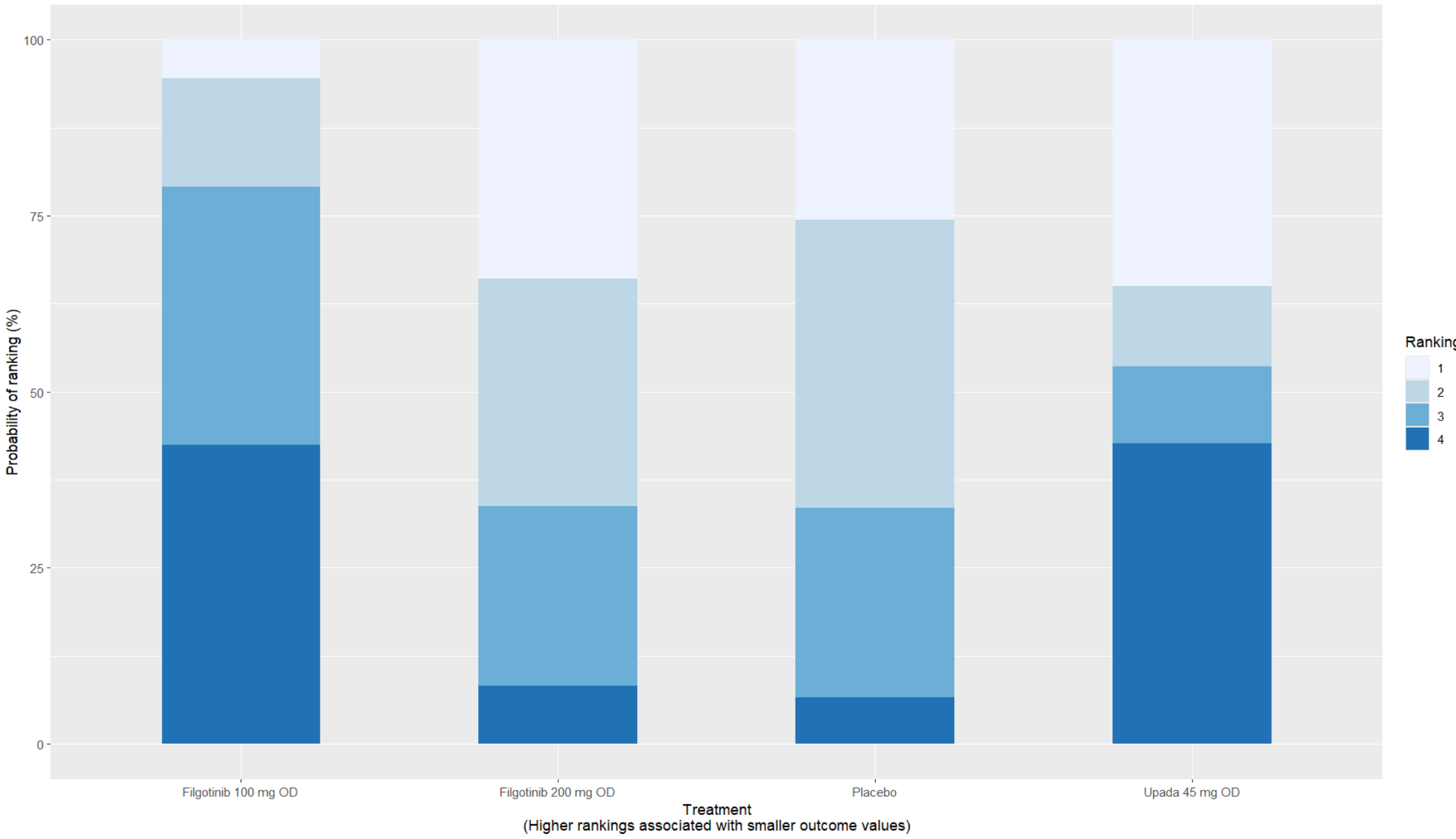

Supplementary Figure S7E: Rankogram for GI perforation

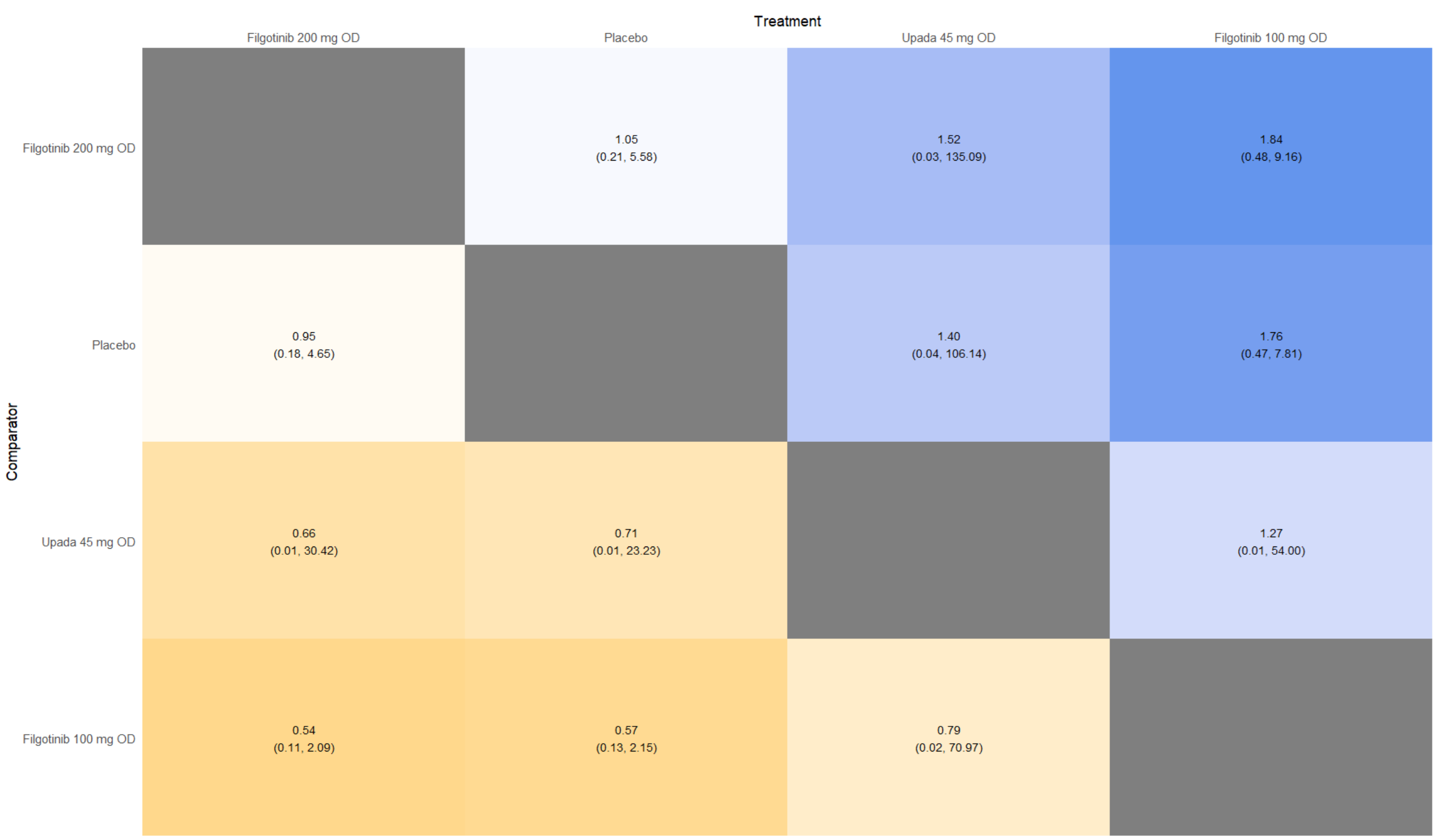

Supplementary Figure S7F: League table for GI perforation

DRUG DISCONTINUATION DUE TO ADVERSE EVENTS:

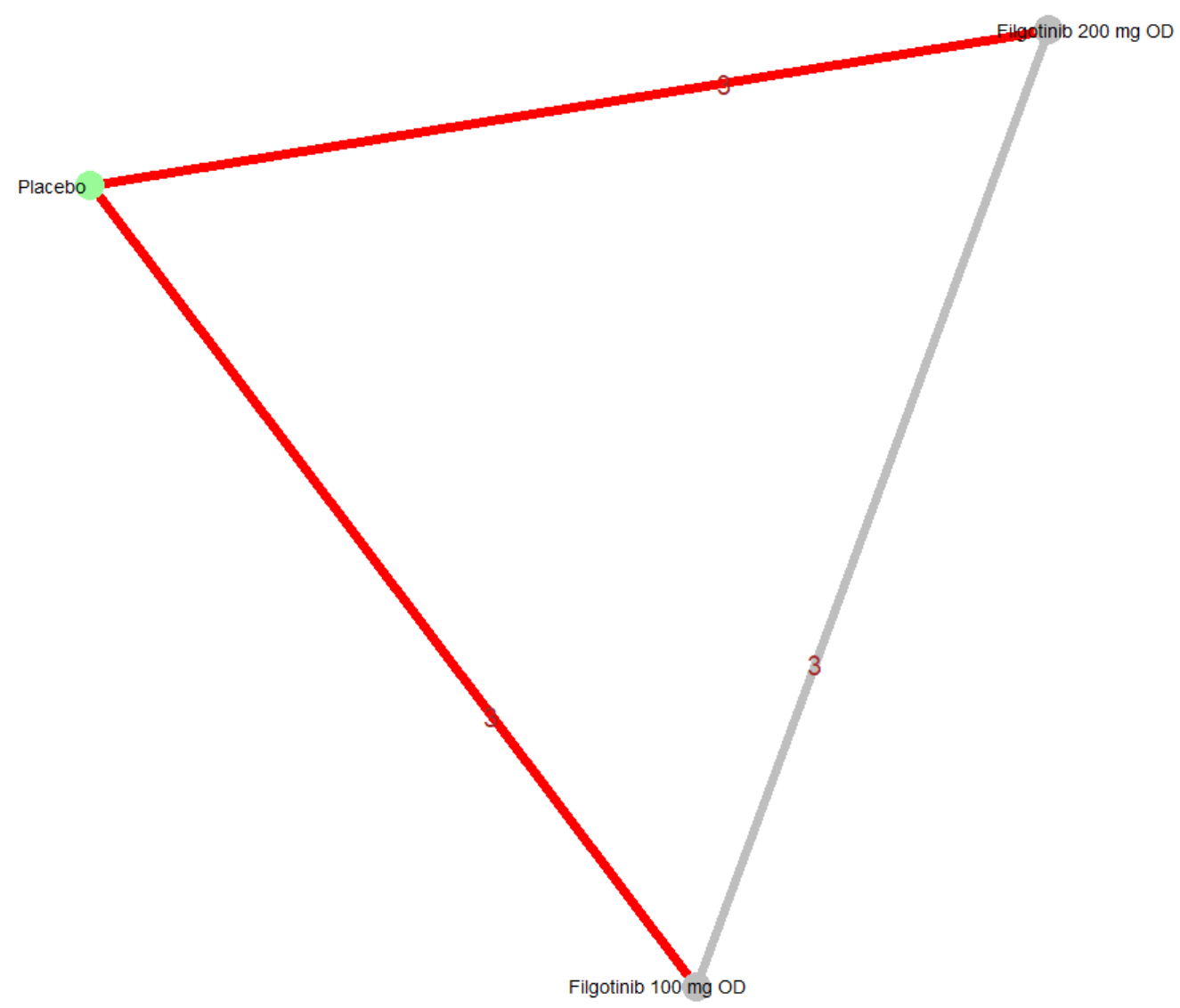

Supplementary Figure S8A: Network geometry plot for drug discontinuation due to adverse events

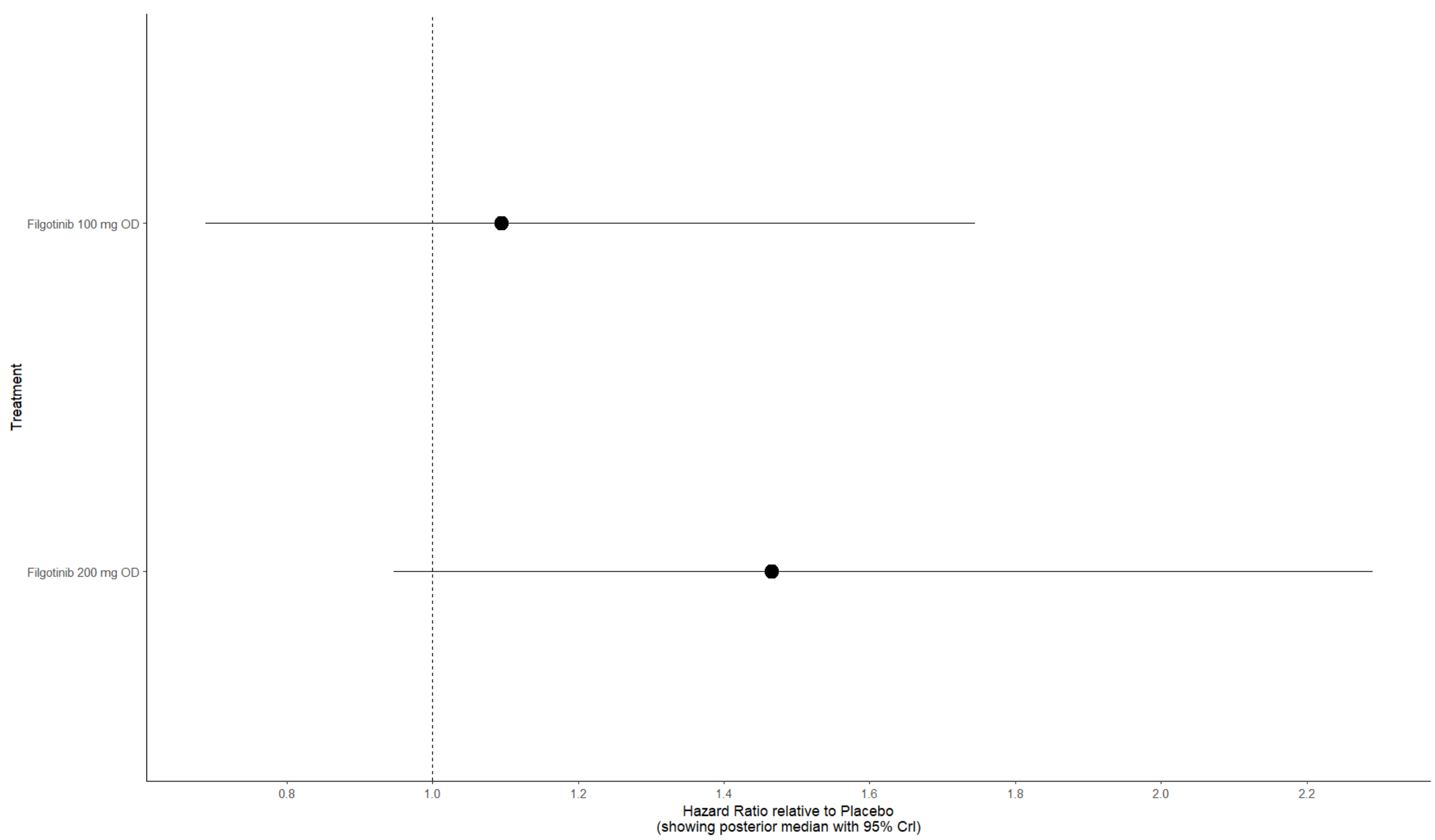

Supplementary Figure S8B: Forest plot for drug discontinuation due to adverse events

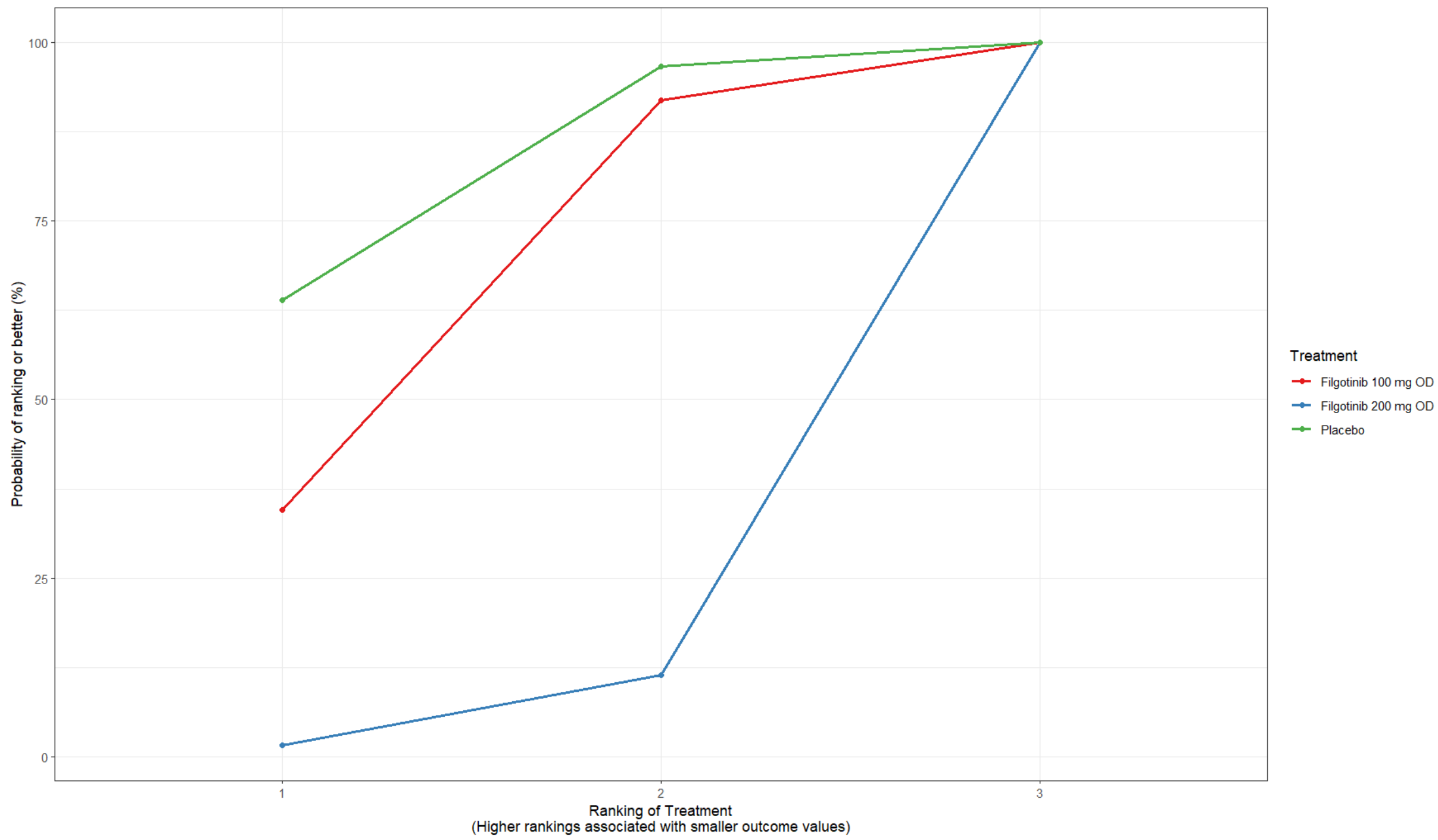

Supplementary Figure S8C: SUCRA plot for drug discontinuation due to adverse events

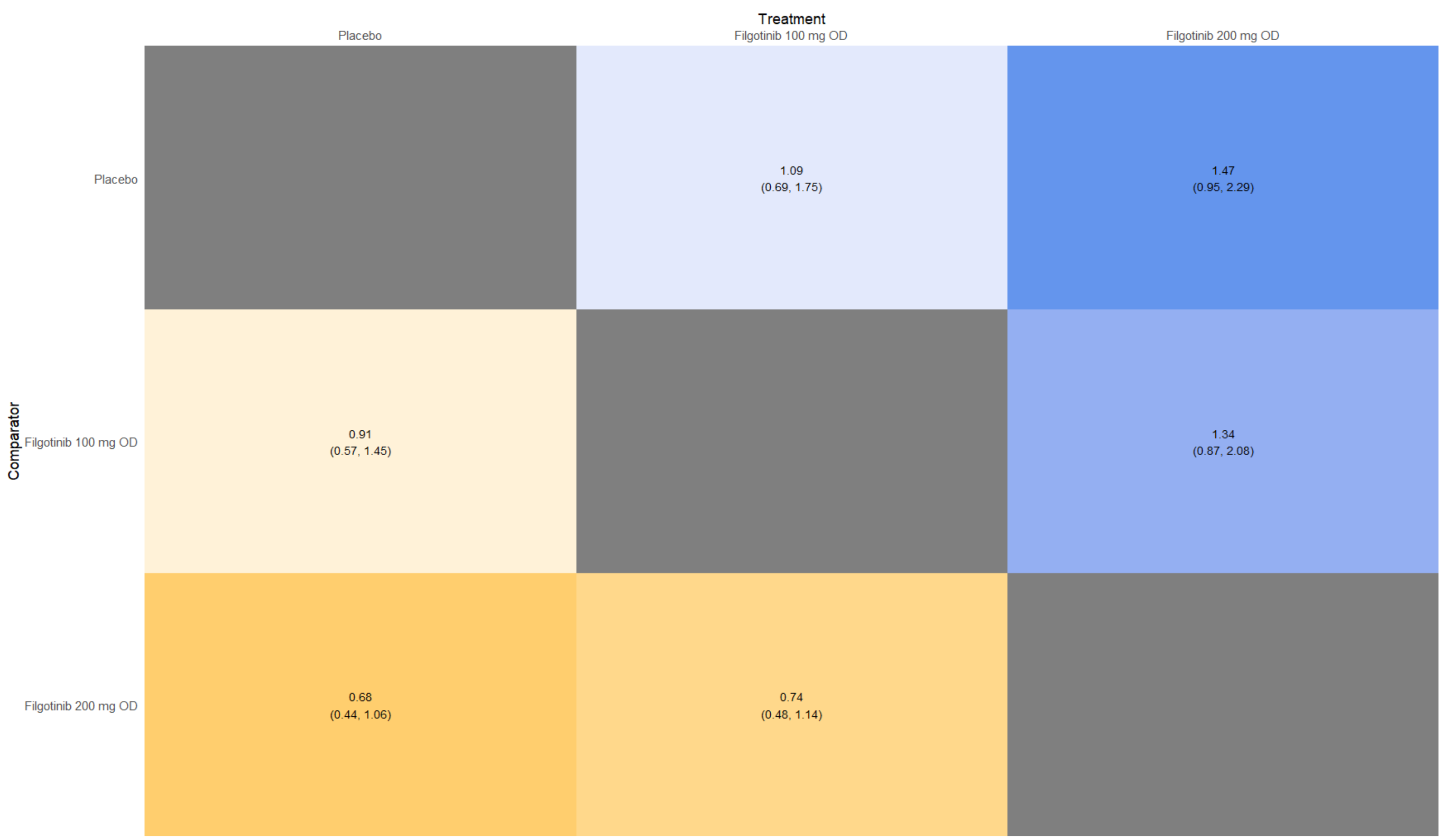

Supplementary Figure S8D: League table for drug discontinuation due to adverse events

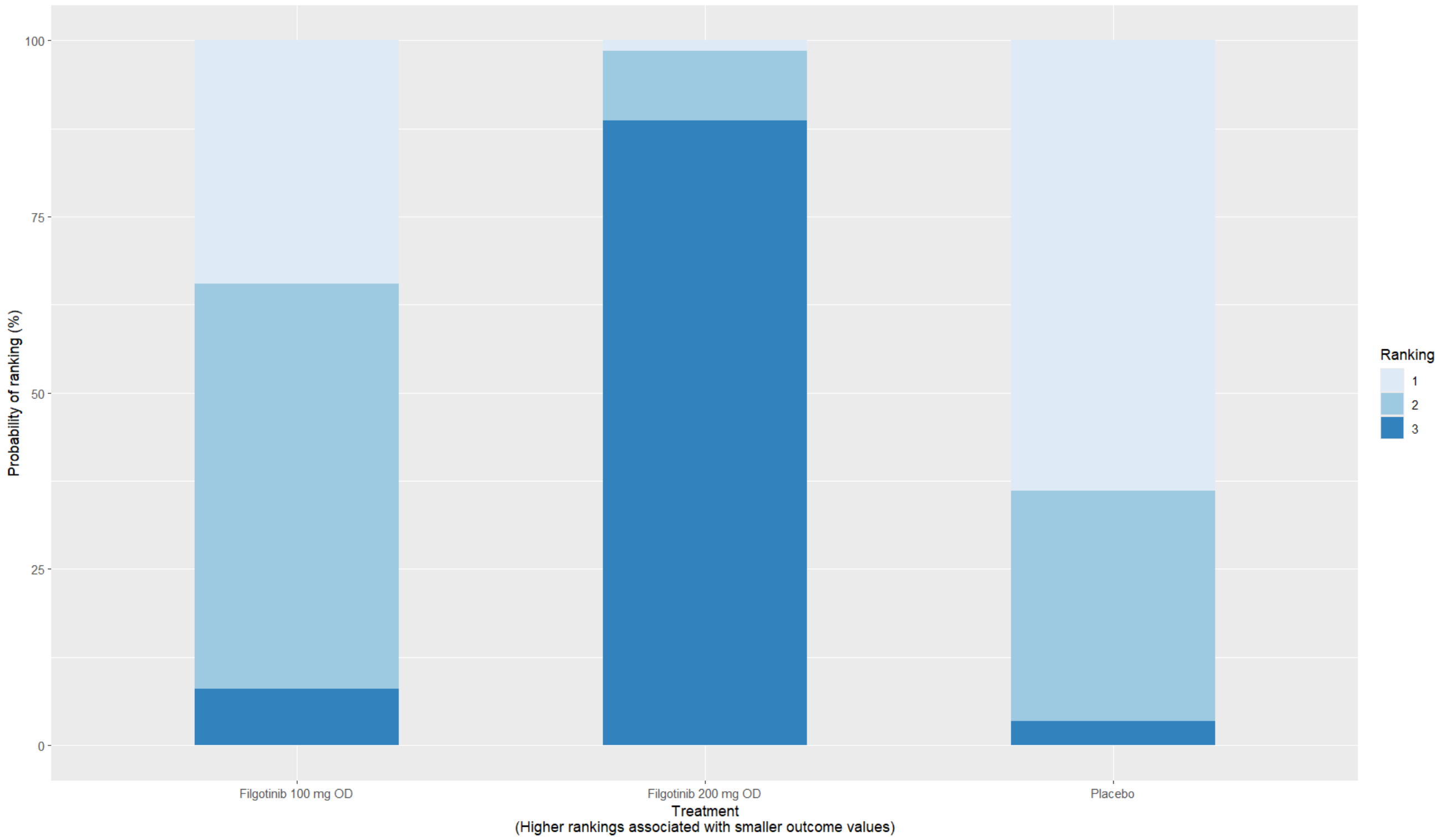

Supplementary Figure S8E: Rankogram for drug discontinuation due to adverse events

IBDQ AT WEEK 12:

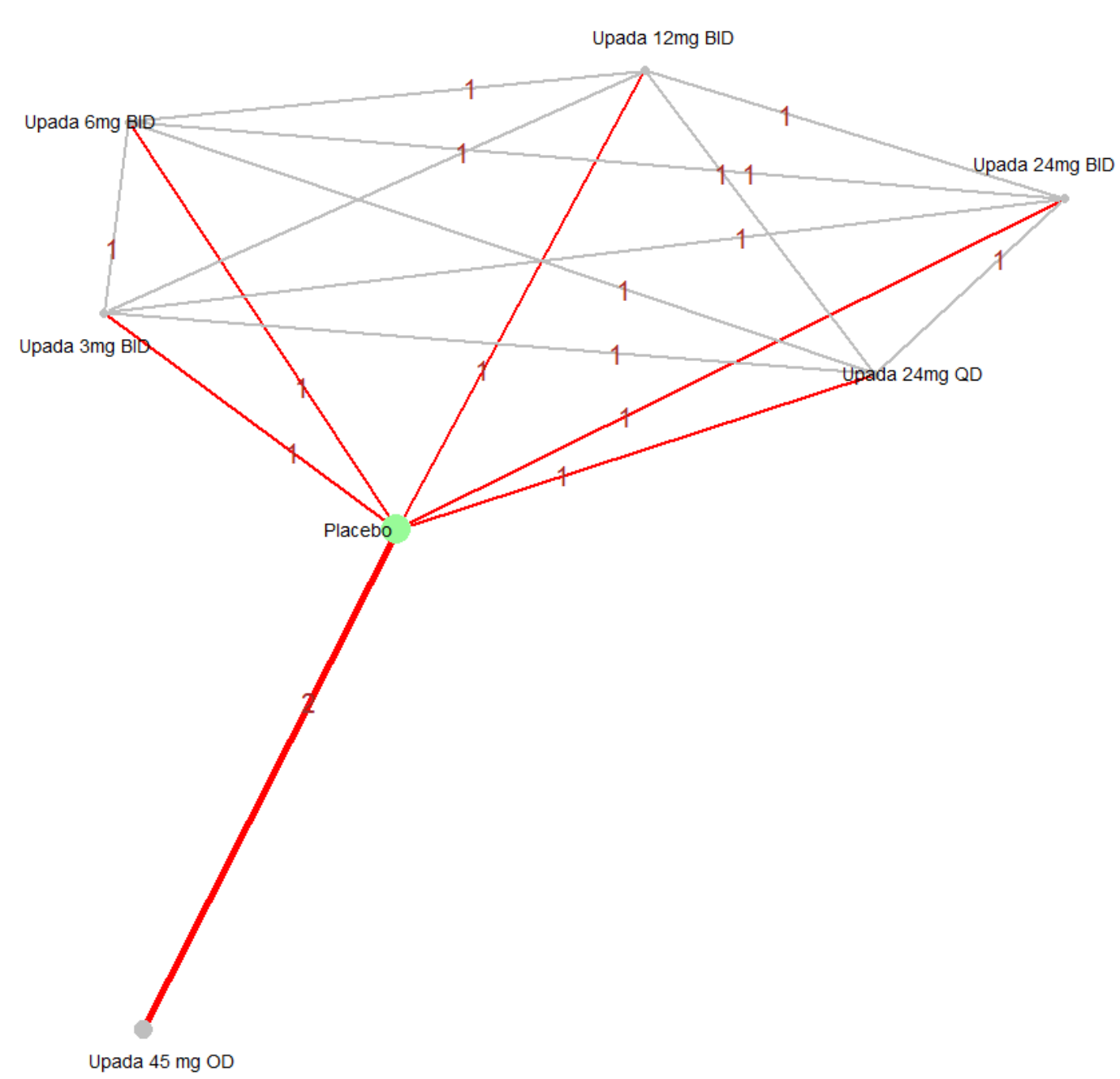

Supplementary Figure S9A: Network geometry plot for IBDQ at week 12

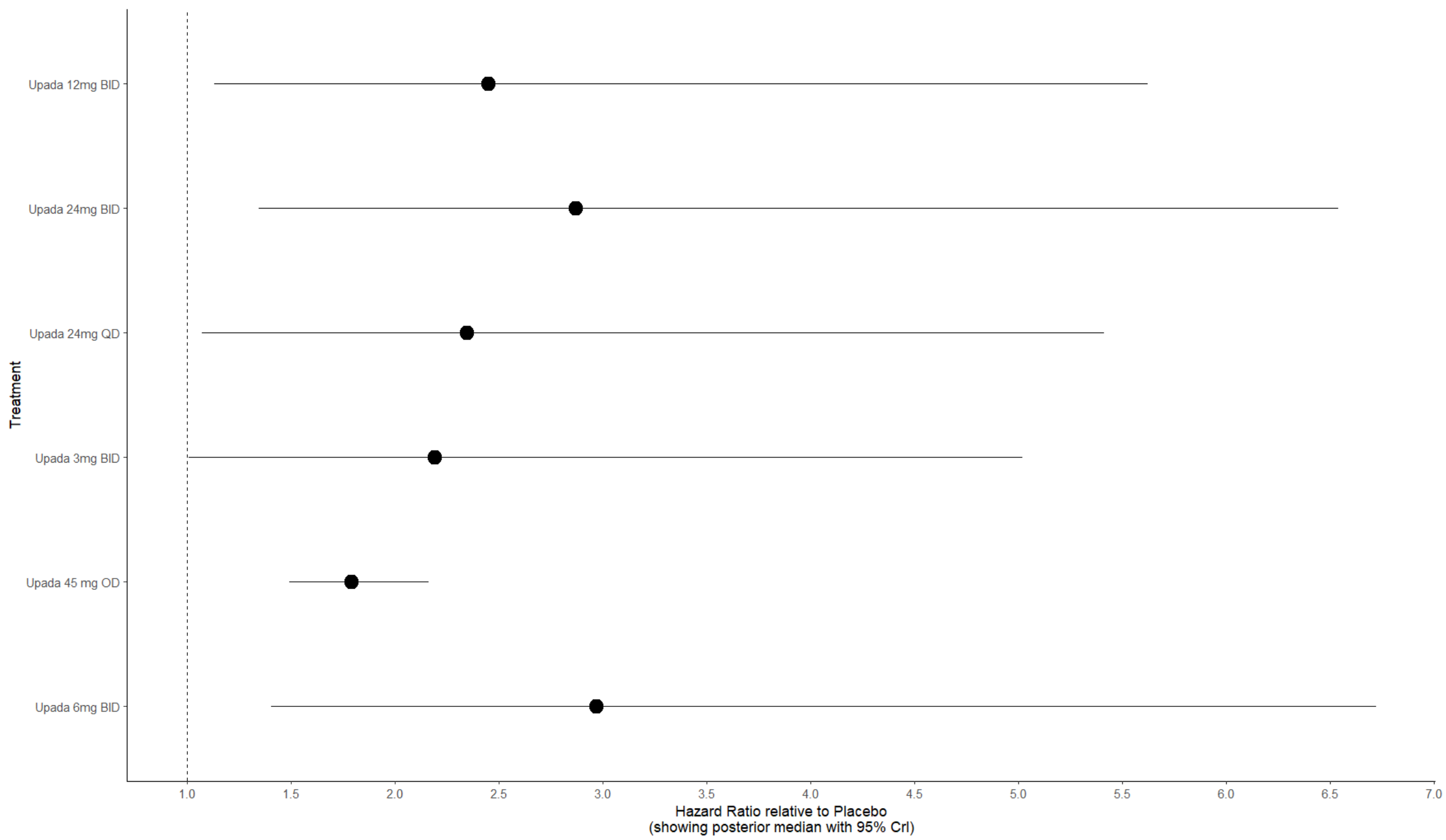

Supplementary Figure S9B: Forest plot for IBDQ at week 12

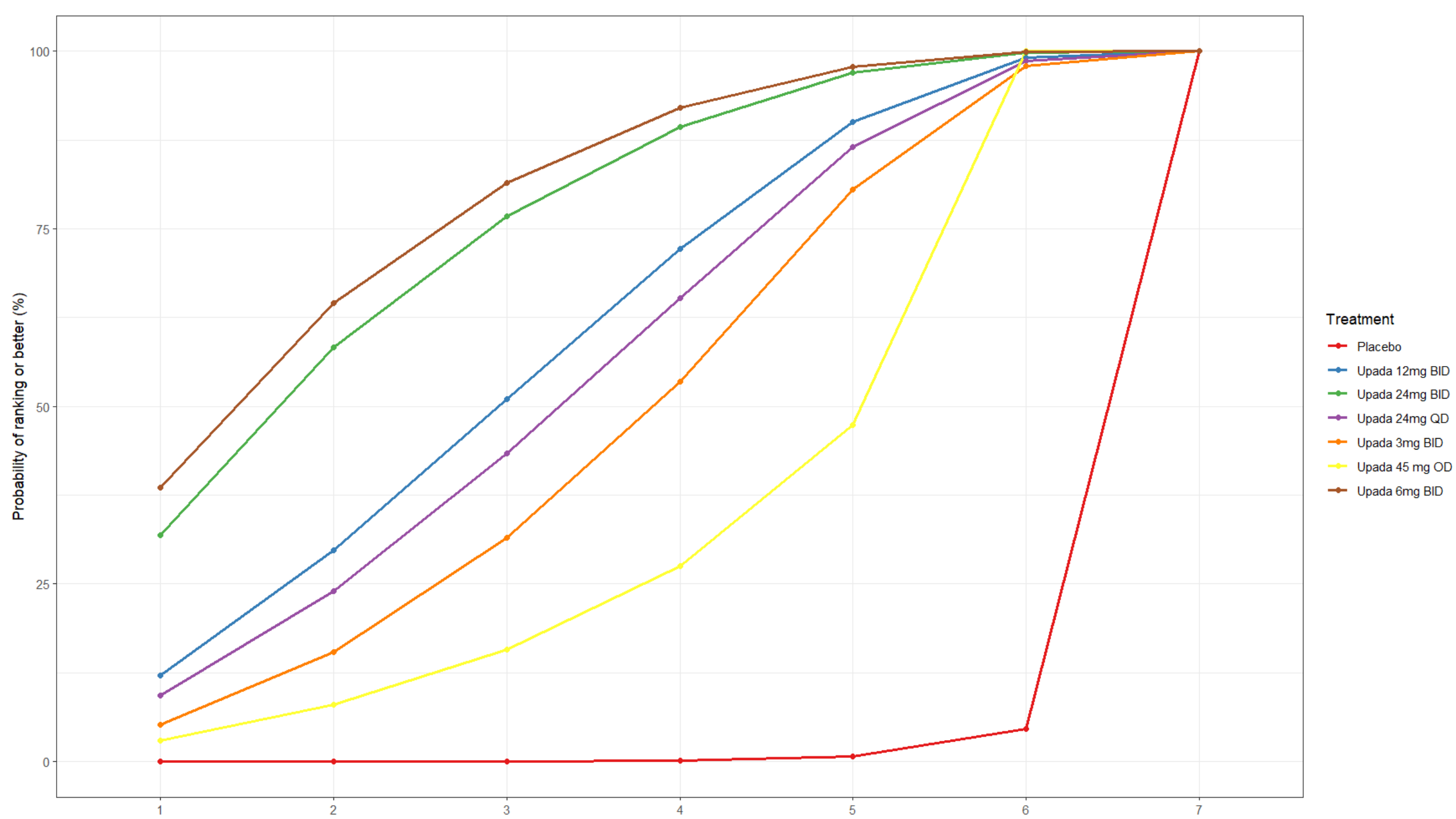

Supplementary Figure S9C: SUCRA plot for IBDQ at week 12

Treatment

Comparator	Upada 6mg BID	Upada 24mg BID	Upada 12mg BID	Upada 24mg QD	Upada 3mg BID	Upada 45 mg OD	Placebo
Upada 6mg BID		0.97<br>(0.51, 1.83)	0.82<br>(0.43, 1.58)	0.79<br>(0.40, 1.52)	0.74<br>(0.38, 1.41)	0.60<br>(0.26, 1.30)	**0.34**<br>(0.15, 0.71)
Upada 24mg BID	1.04<br>(0.55, 1.96)		0.85<br>(0.44, 1.65)	0.82<br>(0.42, 1.59)	0.76<br>(0.39, 1.47)	0.62<br>(0.27, 1.36)	**0.35**<br>(0.15, 0.74)
Upada 12mg BID	1.21<br>(0.63, 2.34)	1.17<br>(0.61, 2.28)		0.96<br>(0.48, 1.90)	0.89<br>(0.45, 1.76)	0.73<br>(0.31, 1.62)	**0.41**<br>(0.18, 0.88)
Upada 24mg QD	1.27<br>(0.66, 2.48)	1.22<br>(0.63, 2.41)	1.04<br>(0.53, 2.07)		0.93<br>(0.47, 1.85)	0.76<br>(0.33, 1.71)	**0.43**<br>(0.18, 0.93)
Upada 3mg BID	1.36<br>(0.71, 2.62)	1.31<br>(0.68, 2.55)	1.12<br>(0.57, 2.20)	1.07<br>(0.54, 2.12)		0.82<br>(0.35, 1.81)	**0.46**<br>(0.20, 0.99)
Upada 45 mg OD	1.66<br>(0.77, 3.83)	1.60<br>(0.74, 3.72)	1.36<br>(0.62, 3.20)	1.31<br>(0.58, 3.07)	1.22<br>(0.55, 2.86)		**0.56**<br>(0.46, 0.67)
Placebo	**2.97**<br>(1.41, 6.73)	**2.87**<br>(1.35, 6.54)	**2.45**<br>(1.13, 5.62)	**2.35**<br>(1.07, 5.41)	**2.19**<br>(1.01, 5.02)	**1.79**<br>(1.49, 2.16)	

Supplementary Figure S9D: League table for IBDQ at week 12

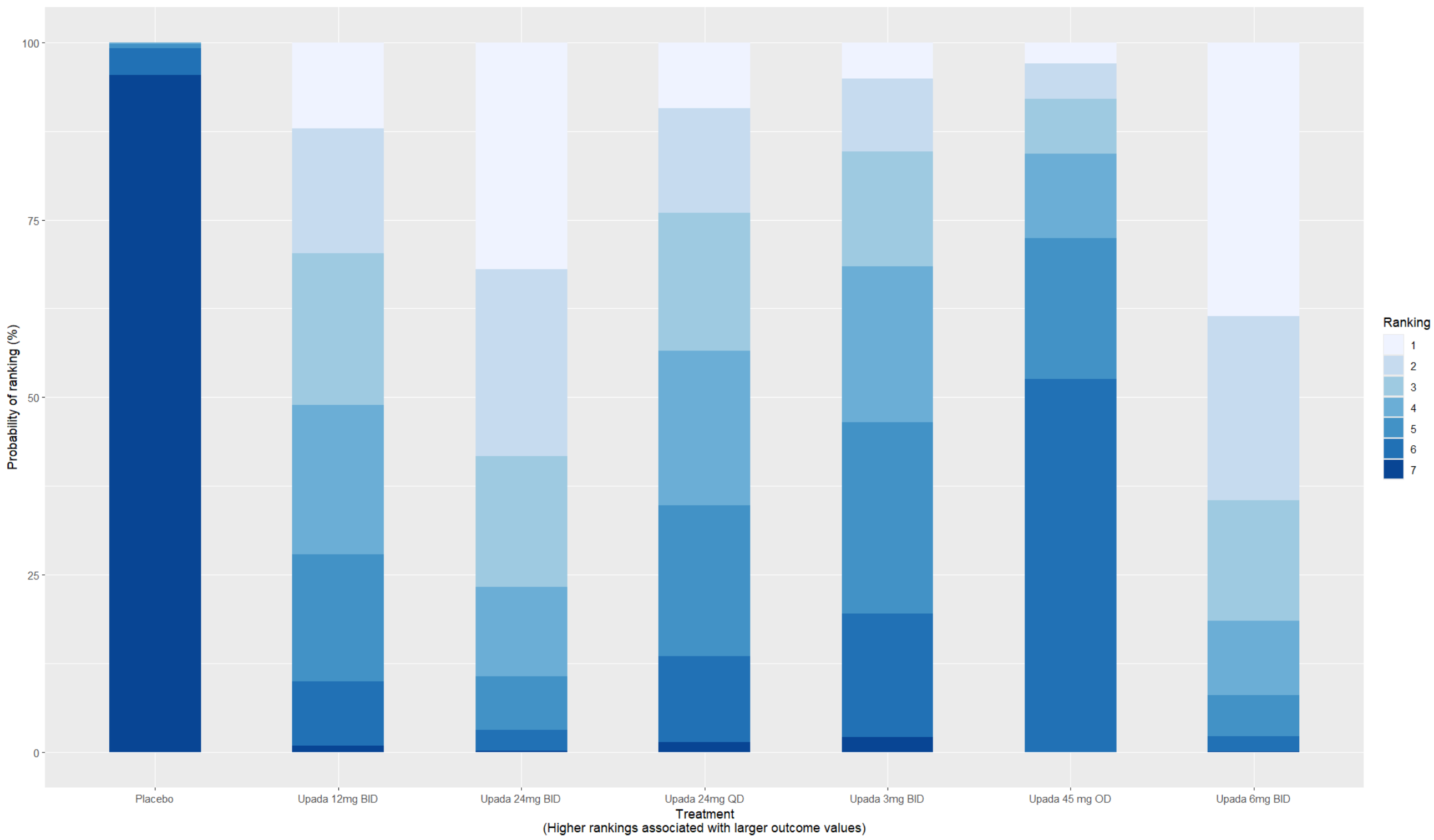

Supplementary Figure S9E: Rankogram for IBDQ at week 12
